# Immunogenicity and seroefficacy of 10-valent and 13-valent pneumococcal conjugate vaccines: a systematic review and network meta-analysis of individual participant data

**DOI:** 10.1016/j.eclinm.2023.102073

**Published:** 2023-07-01

**Authors:** Shuo Feng, Julie McLellan, Nicola Pidduck, Nia Roberts, Julian P.T. Higgins, Yoon Choi, Alane Izu, Mark Jit, Shabir A. Madhi, Kim Mulholland, Andrew J. Pollard, Beth Temple, Merryn Voysey

**Affiliations:** a Department of Paediatrics, Oxford Vaccine Group, University of Oxford, Oxford, UK; b Nuffield Department of Primary Care Health Sciences, University of Oxford, Oxford, UK; c Bodleian Health Care Libraries, University of Oxford, Oxford, UK; d Population Health Sciences, Bristol Medical School, University of Bristol, Bristol, UK; e Modelling and Economics Unit, UK Health Security Agency, London, UK; f South African Medical Research Council MRC Vaccines and Infectious Diseases Analytics Research Unit, Infectious Diseases and Oncology Research Institute, Faculty of Health Sciences, University of the Witwatersrand, Johannesburg, South Africa; g Department of Infectious Disease Epidemiology, London School of Hygiene & Tropical Medicine, London, UK; h Wits Infectious Diseases and Oncology Research Institute, Faculty of Health Sciences, University of the Witwatersrand, Johannesburg, South Africa; i Murdoch Children's Research Institute, Melbourne, VIC, Australia; j Global and Tropical Health Division, Menzies School of Health Research, Charles Darwin University, Darwin, NT, Australia; k NIHR Oxford Biomedical Research Centre, Oxford, UK

**Keywords:** Pneumococcal conjugate vaccines, Network meta-analysis, Individual participant data, Immunogenicity, Seroefficacy

## Abstract

**Background:**

Vaccination of infants with pneumococcal conjugate vaccines (PCV) is recommended by the World Health Organization. Evidence is mixed regarding the differences in immunogenicity and efficacy of the different pneumococcal vaccines.

**Methods:**

In this systematic-review and network meta-analysis, we searched the Cochrane Library, Embase, Global Health, Medline, clinicaltrials.gov and trialsearch.who.int up to February 17, 2023 with no language restrictions. Studies were eligible if they presented data comparing the immunogenicity of either PCV7, PCV10 or PCV13 in head-to-head randomised trials of young children under 2 years of age, and provided immunogenicity data for at least one time point after the primary vaccination series or the booster dose. Publication bias was assessed via Cochrane's Risk Of Bias due to Missing Evidence tool and comparison-adjusted funnel plots with Egger's test. Individual participant level data were requested from publication authors and/or relevant vaccine manufacturers. Outcomes included the geometric mean ratio (GMR) of serotype-specific IgG and the relative risk (RR) of seroinfection. Seroinfection was defined for each individual as a rise in antibody between the post-primary vaccination series time point and the booster dose, evidence of presumed subclinical infection. Seroefficacy was defined as the RR of seroinfection. We also estimated the relationship between the GMR of IgG one month after priming and the RR of seroinfection by the time of the booster dose. The protocol is registered with PROSPERO, ID CRD42019124580.

**Findings:**

47 studies were eligible from 38 countries across six continents. 28 and 12 studies with data available were included in immunogenicity and seroefficacy analyses, respectively. GMRs comparing PCV13 vs PCV10 favoured PCV13 for serotypes 4, 9V, and 23F at 1 month after primary vaccination series, with 1.14- to 1.54- fold significantly higher IgG responses with PCV13. Risk of seroinfection prior to the time of booster dose was lower for PCV13 for serotype 4, 6B, 9V, 18C and 23F than for PCV10. Significant heterogeneity and inconsistency were present for most serotypes and for both outcomes. Two-fold higher antibody after primary vaccination was associated with a 54% decrease in risk of seroinfection (RR 0.46, 95% CI 0.23–0.96).

**Interpretation:**

Serotype-specific differences were found in immunogenicity and seroefficacy between PCV13 and PCV10. Higher antibody response after vaccination was associated with a lower risk of subsequent infection. These findings could be used to compare PCVs and optimise vaccination strategies.

**Funding:**

The 10.13039/501100000664NIHR Health Technology Assessment Programme.

**Research in context**

**Evidence before this study**

The World Health Organization (WHO) recommends vaccination of all children worldwide with at least 3 doses of a licensed pneumococcal conjugate vaccine (PCV) in infancy and does not recommend one product over another. A 2017 systematic review of pneumococcal vaccines which reviewed all data on different pneumococcal vaccine products, included five head-to-head studies comparing PCV13 vs PCV10. This review identified differences in immunogenicity between PCV10 and PCV13 after the primary series and after the booster dose, showing that PCV13 induced higher antibody than PCV10 in some common serotypes at both time points, e.g. serotypes 1, 5, 7F and 23F, while evidence was mixed for other serotypes. The review did not contain a meta-analysis, or head-to-head comparisons of the protection provided by different PCVs.

**Added value of this study**

We estimated serotype-specific difference in antibody responses and seroinfection between PCV13 and PCV10 and showed that for some serotypes, PCV13 induces higher antibody responses. Higher antibody responses corresponded with higher levels of protection against seroinfection (a proxy for carriage) such that in our models comparing two vaccines, a two-fold higher antibody response with one vaccine resulted in a 54% reduction in seroinfection (Relative Risk (RR) 0.46, 95% CI 0.23–0.96). Additionally, we found that PCVs from different manufacturers that produce equivalent levels of antibody provide comparable levels of protection against subclinical infections.

**Implications of all the available evidence**

Our findings suggest that PCV13 provides better protection against subclinical infection for some, but not all serotypes. Evidence from this network meta-analysis could help to guide vaccination strategies, and we recommend considering these serotype-specific differences in efficacy in future PCV health-economic evaluation. These findings also emphasise the importance of higher antibody responses when considering the rollout of new PCVs especially for serotypes that have suboptimal protection with current vaccines.

## Introduction

*Streptococcus pneumoniae* (pneumococcus) causes severe disease including bacterial pneumonia, meningitis, and sepsis, leading to substantial morbidity and mortality worldwide, with the highest disease burden being in young children and older adults.^1^^,^^2^ There have been more than 100 serotypes of pneumococcus documented as of 2020, not all of which cause severe disease, and the distribution of these serotypes varies substantially between countries.^1^^,^^2^ Three pneumococcal conjugate vaccines (PCV)s, have been widely deployed in the past two decades: PCV7 (Prevnar, Pfizer), PCV10 (Synflorix, GSK) and PCV13 (Prevenar 13, Pfizer), resulting in substantial reduction in disease.^1^^,^^3^ New PCVs such as PCV15, PCV20 and PCV10–SII have been recently licensed but have yet to be widely implemented.

Currently, three PCVs are recommended by the World Health Organization (WHO) for infants worldwide: PCV13, PCV10, and a new 10-valent PCV manufactured by Serum Institute of India (PCV10-SII, PNEUMOSIL) which was prequalified by WHO in December 2019.^4^, ^5^, ^6^ PCV13 contains three additional serotypes (3, 6A and 19A) to the 10 serotypes included in PCV10 (serotype 1, 4, 5, 6B, 7F, 9V, 14, 18C, 19F, and 23F). PCV10-SII covers serotypes 1, 4, 5, 6B, 7F, 9V, 14, 18C, 19F and 23F. The licensure of PCVs is benchmarked against anti-capsular IgG antibody responses above a threshold of 0.35 mcg/mL for all vaccine serotypes, which was established using data from three randomised controlled efficacy trials.^7^

The WHO does not preferentially endorse one PCV over another. Both PCV13 and PCV10 have been shown to provide both direct and indirect protection against pneumococcal pneumonia, invasive pneumococcal disease and nasopharyngeal carriage.^3^^,^^6^^,^^8^ Although there are 10 common serotypes in these two vaccines the content of the vaccines differ, with different carrier proteins used in the conjugation process, as well as different amounts of polysaccharide, and these differences may contribute to differences in protection. In 2017 a systematic review of head-to-head studies comparing PCV10 vs PCV13 showed differences in anti-pneumococcal IgG responses between vaccines.^9^ However, no meta-analysis was included in this review and there remains uncertainty over whether one vaccine is consistently more immunogenic, and whether differences in immunogenicity result in clinically important differences in protection.^9^ Large head-to-head randomised controlled trials of PCVs with invasive pneumococcal disease as the primary outcome are not feasible. Studies that assessed the impact of different PCVs on nasopharyngeal carriage have reported very few or no differences.^10^^,^^11^ Episodes of nasopharyngeal carriage often last only a few days or weeks therefore cross-sectional swabbing studies may misclassify participants when swabs are not taken at the time of infection, resulting in underpowered comparisons. We previously used seroinfection as an outcome for estimating correlates of protection for PCVs against pneumococcal carriage,^12^ where seroinfection is defined as an increase in antibody levels between the primary vaccination series (typically at 5–7 months of age) and the booster dose (typically at 9–18 months of age). Seroinfection can be regarded as evidence of exposure to the pathogen and a resultant sub-clinical infection, given antibody responses wane rapidly during this period otherwise.^12^

In this study, we meta-analysed individual participant data from head-to-head studies of PCVs to compare the immunogenicity and relative risk of seroinfection (seroefficacy) of PCV10 with PCV13 for each serotype. We aimed to determine if serotype-specific immune responses were higher for either vaccine, and whether this resulted in greater protection again seroinfection for the same serotypes. In addition, we explored the overall relationship between the higher immune response and protection against seroinfection.

## Methods

Our systematic review is reported in line with the recommendations from the Preferred Reporting Items for Systematic Reviews and Meta-Analyses statement plus the extension statements for network and individual patient data systematic reviews.^13^, ^14^, ^15^

### Primary and secondary objectives

The primary objective was to compare the immunogenicity of PCV10 vs PCV13 for each serotype contained in the vaccines. The secondary objectives were: 1) to compare the seroefficacy of PCV10 vs PCV13 for each serotype contained in the vaccines, 2) for PCV10 and PCV13 separately, to estimate immunogenicity and seroefficacy in comparison to the older PCV7 vaccine, and 3) to determine how the comparisons of immunogenicity and efficacy of PCV10 to PCV13 are affected by the co-administration of different routine vaccines.

### Systematic review

We conducted a systematic review identifying studies that compared the immunogenicity of licensed PCVs for infants or children in head-to-head randomised trials. The PCVs included in the review were PCV13, PCV10 and PCV7. The last was included so that we could compare PCV13 and PCV10 indirectly through them each being compared with PCV7 for the same serotypes.

### Search strategy

The search strategy was devised and conducted by an information specialist (NR). Five databases and two trial registers were searched from database inception to 17th February 2023. No date or language limits were applied. Full search criteria are listed in the Supplemental material.

### Study selection

Two reviewers (JM, NP) independently reviewed the title and abstract of each reference and identified potentially relevant references. Two reviewers (JM, NP) independently selected studies to be included in the review from retrieved full-text papers using predetermined inclusion criteria. Disagreements about study inclusion were resolved by a third reviewer (MV).

Randomised controlled trials were included if they provided head-to-head comparisons of either PCV7, PCV10, or PCV13 among infants and children less than 2 years of age, and if they provided estimates on antibody responses (serotype-specific anti-pneumococcal IgG) to PCVs for at least one time point of 1) between 4 and 6 weeks after the primary vaccination series, and/or one-month after a booster vaccination. Trials were eligible only if they included at least one of the three vaccines of interest (PCV10, PCV13, PCV7. Trials were excluded that enrolled immuno-compromised (e.g. HIV) children.

### Data retrieval

For all eligible trials, the publication authors/data owners were approached for trial and individual participant level data. Baseline characteristics and potential effect modifiers were extracted for participants’ age, sex, country, immunogenicity assays, co-administered study vaccines and vaccine schedules.

Aggregate data from publications were extracted if individual participant data were not available. Data extraction of published results and individual participant level data were independently completed by SF and MV. Individual participant data completeness was examined, and baseline characteristics distribution were compared between studies with and without individual participant data.

### Assessment of risk of bias in included studies

Risk of bias in results of the included studies was assessed independently by two reviewers (JM, NP) using the Cochrane Risk of Bias Tool (RoB 2).^16^ This considers the risk of bias in five domains (randomisation process, deviations from the intended interventions, missing outcome data, measurement of the outcome, selection of the reported result) and generates an overall risk of bias. The possible risk of bias judgments for each domain, and overall, are ‘low risk of bias’, ‘some concerns’ and ‘high risk of bias’. Disagreements between reviewers were resolved by consensus. Results for the risk of bias assessment were presented using robvis (visualisation tool).^17^ Publication bias was assessed via Cochrane's Risk Of Bias due to Missing Evidence tool and comparison-adjusted funnel plots with Egger's test. Full details are given in the Supplemental material.

### Assessment of heterogeneity and inconsistency of network meta-analysis

To assess the statistical heterogeneity and inconsistency of NMA, we evaluated the transitivity assumption by visually comparing the distribution of the baseline characteristics and potential effect modifiers across the different pairwise comparisons. We assessed the presence of heterogeneity using estimated values of the heterogeneity variance parameters (τ) and the I-squared statistic and its 95% confidence interval that measures the percentage of variability in point estimates that cannot be attributed to random error, and estimated the Q statistic. We evaluated the inconsistency, i.e. coherence between direct and indirect evidence, using a Q statistic,^18^ which measures the deviation from consistency. The random-effects model was fitted following the graph-theoretical approach and using the GMR and RR as effect estimate with 95% CI.^19^

Some individual participant level data were missing due to laboratory errors, insufficient blood sample volume or participant withdrawal. Data were not imputed and missing data were considered missing-completely-at-random. Individual participant level data were analysed according to the vaccine received.

### Outcomes

The primary outcome was serotype-specific anti-capsular pneumococcal immunoglobulin G. Antibodies measured one-month after the primary series of 1–3 doses in infancy, prior to a booster dose, and one-month post-booster dose were included. The outcome for seroefficacy analyses was the difference between log_10_-transformed serotype-specific anti-pneumococcal IgG measured one-month after the primary series of doses and prior to administration of the booster dose.

### Statistical analysis

#### Immunogenicity

Each trial that had individual participant level data available was analysed to obtain the log of the ratio of geometric means (log-GMR) and its standard error, for each serotype and time point of interest. If individual participant data were unavailable, published GMR estimates and confidence intervals were used. The estimates combined from individual participant data and aggregate data formed the input data for data synthesis.

#### Seroefficacy

As a binary variable, seroinfection was equivalent to 1 if antibody levels increased by any amount, or 0 otherwise. To assess the seroefficacy of the PCVs, we calculated the proportion of participants with seroinfection in each vaccine group and calculated seroefficacy as the relative risk (RR) of seroinfection. When no seroinfection occurred in any group (numerator of absolute risk was 0), a small nonzero value (0.5) was added to both numerator and denominator to allow estimation of the RR. The log-RRs and their standard errors were then the input data for evidence synthesis. Only trials supplying individual participant data were included in seroefficacy analyses.

#### Data synthesis by network meta-analysis and meta-analysis

Serotypes 4, 6B, 9V, 14, 18C, 19F, and 23F were contained in all three vaccines, therefore evidence could be synthesized using a network meta-analysis of all comparisons between PCVs, including PCV7 (see Supplementary Fig. S1). Serotypes 1, 5, 7F, 3, 6A and 19A are only included in PCV10 and PCV13 vaccines therefore for these serotypes evidence was synthesized by meta-analysing studies that directly compared PCV13 vs PCV10.

For the analysis of immunogenicity, we synthesized evidence for all PCV13 serotypes, However, seroefficacy could only be assessed in situations where the serotypes of interest were included in both vaccines and therefore seroefficacy of serotypes 3, 6A, and 19A could not be assessed as these are only included in one vaccine (PCV13). Sensitivity analysis is described in Supplementary material.

#### Association between ratios of immunogenicity and seroefficacy

To estimate separate serotype-specific relationships between the GMRs and RRs, study level data were combined regressing the RR of seroinfection on the GMR using linear regression models weighted by the sample size of the study. Weighted Pearson correlation coefficients were calculated.

To estimate the overall association between antibody GMR and RR across all serotypes, we fitted a mixed-effect model regressing study-level RRs of seroinfection on GMRs across serotypes, weighted by the sample size of each study. Fixed-effects included GMR, serotype, and interactions between GMR and serotype (allowing serotype-specific association), while study was included as a random effect. As a sensitivity analysis, we reversed both RRs and GMRs estimated (i.e. PCV13 vs PCV7 was changed to PCV7 vs PCV13). By shifting comparators, we aimed to evaluate of the stability of the association estimates.

To evaluate if differences between products from two different manufacturers change the relationship between antibody levels and protection, we assessed the association between immunogenicity and seroefficacy restricting to studies that compared PCV13 vs PCV10 and PCV7 vs PCV10 only (comparisons between PCV13 and PCV7 were removed from analysis as these vaccines are from the same manufacturer). We examined whether PCVs of different manufacturers that produce equivalent levels of antibody (GMR = 1) also provide comparable seroefficacy (RR = 1).

All analyses were performed in R 4.2.2. NMA and meta-analysis were conducted using the netmeta and metafor packages.^18^^,^^19^

### Role of the funding source

The funder of the study had no role in study design, data collection, data analysis, data interpretation, or writing of the paper.

## Results

**Fig. 1:**
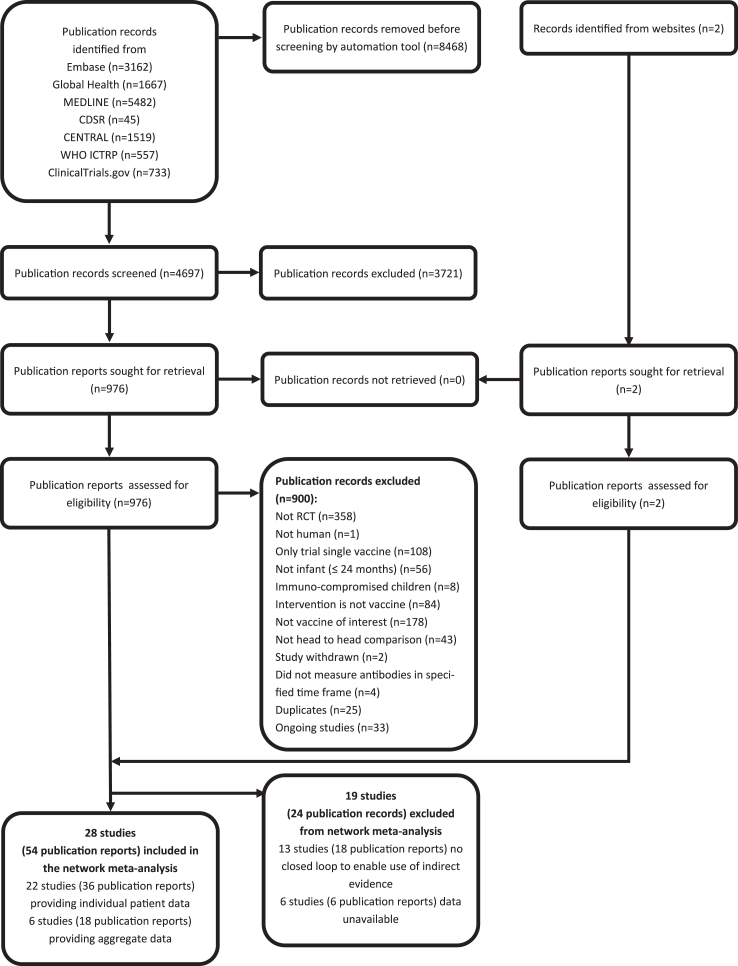
**PRISMA flow diagram to show study selection process**.

Database registry and hand searches identified 4699 publication records (Fig. 1), of which 47 studies (78 publication reports) satisfied our eligibility criteria.^10^^,^^11^^,^^20^, ^21^, ^22^, ^23^, ^24^, ^25^, ^26^, ^27^, ^28^, ^29^, ^30^, ^31^, ^32^, ^33^, ^34^, ^35^, ^36^, ^37^, ^38^, ^39^, ^40^, ^41^, ^42^, ^43^, ^44^, ^45^, ^46^, ^47^, ^48^, ^49^, ^50^, ^51^, ^52^, ^53^, ^54^, ^55^, ^56^, ^57^, ^58^, ^59^, ^60^, ^61^, ^62^, ^63^, ^64^, ^65^, ^66^, ^67^, ^68^, ^69^, ^70^, ^71^, ^72^, ^73^, ^74^, ^75^, ^76^, ^77^, ^78^, ^79^, ^80^, ^81^, ^82^, ^83^, ^84^, ^85^, ^86^, ^87^, ^88^, ^89^, ^90^, ^91^, ^92^, ^93^, ^94^, ^95^, ^96^ 19 studies (24 publication reports) were excluded from the analysis: 6 studies did not provide individual patient or aggregate data,^72^, ^73^, ^74^, ^75^ and 13 studies (18 publication reports) were head-to-head studies with the vaccines of interest, but it was not possible to form a loop within the network meta-analysis to provide indirect evidence (See Supplementary Fig. S1 and Supplementary Table S2).^76^, ^77^, ^78^, ^79^, ^80^, ^81^, ^82^, ^83^, ^84^, ^85^, ^86^, ^87^, ^88^, ^89^, ^90^, ^91^^,^^93^, ^94^, ^95^, ^96^ The remaining 28 studies (54 publication records) from 2009 to 2023 were included in the network meta-analyses.^10^^,^^11^^,^^20^, ^21^, ^22^, ^23^, ^24^, ^25^, ^26^, ^27^, ^28^, ^29^, ^30^, ^31^, ^32^, ^33^, ^34^, ^35^, ^36^, ^37^, ^38^, ^39^, ^40^, ^41^, ^42^, ^43^, ^44^, ^45^, ^46^, ^47^, ^48^, ^49^, ^50^, ^51^, ^52^, ^53^, ^54^, ^55^, ^56^, ^57^, ^58^, ^59^, ^60^, ^61^, ^62^, ^63^, ^64^, ^65^, ^66^, ^67^, ^68^, ^69^, ^70^, ^71^^,^^92^

The 28 included studies comprised 31 cohorts of children as one study conducted in two countries reported results separately,^23^^,^^24^ and one study included head-to-head comparisons of 3 vaccination schedules^21^^,^^50^ (Table 1). Studies with multiple NCT numbers or publications but the same population were counted as one cohort. These 31 cohorts were representative of 38 countries in six continents—Europe (n = 11 cohorts), Asia (n = 9 cohorts), North America (n = 3 cohorts), Africa (n = 3 cohort), Oceania (n = 4 cohort) and South America (n = 1 cohort).

**Table 1:** Summary of studies included in immunogenicity and seroefficacy analyses.

Cohort ID^a^	Author & Year^a^	NCT	Individual participant data available/Aggregate data	comparison	Country/Region	Continent	Schedule	Schedule primary series	Schedule booster	Co-administered Vaccine(s)	Assay
1^23^^,^^24^	Bermal et al. 2009^23^	NCT00344318 NCT00547248	Individual	pcv10 vs pcv7	Philippines	Asia	3 + 1	6-10-24 weeks	12–18 months	DTPw-HBV-Hib-TT + OPV	22F-ELISA
1^23^^,^^24^	Bermal et al. 2009^23^	NCT00344318 NCT00547248	Individual	pcv10 vs pcv7	Poland	Europe	3 + 1	2-4-6 months	12–18 months	DTPw-HBV-Hib-TT + IPV	22F-ELISA
2^39^	Kim et al. 2011^39^	NCT00680914	Individual	pcv10 vs pcv7	Korea	Asia	3 + 1	2-4-6 months	12–18 months	Hib-TT	22F-ELISA
3^41^	Knuf et al. 2012^41^	NCT00307541 NCT00333450	Individual	pcv10 vs pcv7	Germany	Europe	3 + 0	2-3-4 months	NA	DTPa-HBV-Hib-TT/IPV	22F-ELISA
4^55^, ^56^, ^57^	Prymula et al. 2017^57^	NCT01204658	Individual	pcv13 vs pcv10	Czech Republic, Germany, Poland, Sweden	Europe	3 + 1	2-3-4 months	12–15 months	DTPa-HBV-Hib-TT/IPV	22F-ELISA
5^26^	Carmona Martinez et al. 2019^26^	NCT01616459	Individual	pcv13 vs pcv10	Czech Republic, Germany, Poland, Spain	Europe	3 + 1	2-3-4 months	12–15 months	DTPa-HBV-Hib-TT/IPV + MenC-TT (SP)	22F-ELISA
6^10^^,^^48^^,^^49^^,^^59^^,^^60^	Temple et al. 2019^59^	NCT01953510	Individual	pcv13 vs pcv10	Vietnam	Asia	2 + 1	2–4 months	9.5 months	DTPa-HBV-Hib-TT/IPV	modified thirdgeneration standardised ELISA
7^62^, ^63^, ^64^	van den Bergh et al. 2011^62^	NCT00652951	Individual	pcv10 vs pcv7	Netherland	Europe	3 + 1	2-3-4 months	11–13 months	DTPa-(HBV)-Hib-TT/IPV	22F-ELISA
8^66^	Vesikari et al. 2009^66^	NCT00307554 NCT00370396	Individual	pcv10 vs pcv7	Finland, France, and Poland	Europe	3 + 1	2-3-4 months	12–18 months	DTPa-(HBV)-Hib-TT/IPV	22F-ELISA
9^68^	Wysocki et al. 2009^68^	NCT00334334 NCT00463437	Individual	pcv10 vs pcv7	Germany, Poland, and Spain	Europe	3 + 1	2-4-6 months	11–18 months	DTPa-(HBV)-Hib-TT/IPV + Hib MenC-TT	22F-ELISA
10^20^	Amdekar et al. 2013^20^	NCT00452790	Individual	pcv13 vs pcv7	India	Asia	3 + 1	6-10-14 weeks	12 months	DTwP-Hib-HBV + OPV	Standardized ELISA
11^28^, ^29^, ^30^, ^31^, ^32^^,^^37^	Dagan et al. 2013^32^	NCT00508742	Aggregate	pcv13 vs pcv7	Israel	Asia	3 + 1	2-4-6 months	12 months	NA	Standardized ELISA
12^33^	Esposito et al. 2010^33^	NCT00366899	Individual	pcv13 vs pcv7	Italy	Europe	2 + 1	3–5 months	11 months	DTPa-HBV-Hib-TT/IPV	Standardized ELISA
13^35^	Grimprel et al. 2011^35^	NCT00366678	Individual	pcv13 vs pcv7	France	Europe	3 + 1	2-3-4 months	12 months	DTPa-Hib-TT/IPV	Standardized ELISA
14^36^	Huang et al. 2012^36^	NCT00688870	Individual	pcv13 vs pcv7	Taiwan	Asia	3 + 1	2-4-6 months	15 months	DTPa-(HBV)-Hib-TT/IPV	Standardized ELISA
15^27^^,^^34^^,^^38^	Kieninger et al. 2010^38^	NCT00366340	Individual	pcv13 vs pcv7	Germany	Europe	3 + 1	2-3-4 months	11–12 months	DTPa-HBV-Hib-TT/IPV	Standardized ELISA
16^40^^,^^46^	Kim et al. 2013^40^	NCT00689351	Individual	pcv13 vs pcv7	Korea	Asia	3 + 1	2-4-6 months	12 months	DTPa-HBV-Hib-TT/IPV	Standardized ELISA
17^53^	Payton et al. 2013^53^	NCT00444457	Individual	pcv13 vs pcv7	United States	North America	3 + 1	2-4-6 months	12 months	DTPa-HBV-Hib-TT/IPV	Standardized ELISA
18^11^^,^^47^^,^^54^^,^^65^	Pomat et al. 2018^54^	NCT01619462	Aggregate	pcv13 vs pcv10	Papua New Guinea	Oceania	3 + 1	1-2-3 months	9 months	DTPw-HBV-Hib-TT + OPV	WHO standardized ELISA
19^58^	Snape et al. 2010^58^	NCT00384059	Individual	pcv13 vs pcv7	United Kingdom	Europe	2 + 1	2–4 months	12–13 months	DTPa-Hib-TT/IPV/MenC + Hib-MenC-TT	Standardized ELISA
20^61^	Togashi et al. 2015^61^	NCT01200368	Individual	pcv13 vs pcv7	Japan	Asia	3 + 1	enr 3–6 m, 4–8 w int	12–15 months	DTPa	Standardized ELISA
21^67^	Weckx et al. 2012^67^	NCT00676091	Individual	pcv13 vs pcv7	Brazil	South America	3 + 1	2-4-6 months	12 months	HBV-DTwP-Hib/OPV/Rotavirus	Standardized ELISA
22^69^	Yeh et al. 2010^69^	NCT00373958	Individual	pcv13 vs pcv7	United States	North America	3 + 1	2-4-6 months	12–15 months	DTPa-HBV-Hib-TT/IPV	Standardized ELISA
23^70^^,^^71^	Zhu et al. 2016^71^	NCT01692886	Individual	pcv13 vs pcv7	China	Asia	3 + 1	3-4-5 months	12 months	NA	Standardized ELISA
24^25^	*Bryant* et al. *2010*^25^	NCT00205803	Aggregate	pcv13 vs pcv7	United States	North America	3 + 0	2-4-6 months	NA	DTPa-HBV-Hib-TT/IPV	Standardized ELISA
25^51^^,^^52^	Odutola et al. 2017^51^	NCT01262872	Aggregate	pcv13 vs pcv10	Gambia	Africa	3 + 0	2-3-4 months	NA	DTPw-HBV-Hib-TT + OPV	GSK in-house ELISA
26^22^^,^^42^, ^43^, ^44^, ^45^	Leach et al. 2021^44^	NCT01174849	Aggregate	pcv13 vs pcv10	Australia	Oceania	3 + 1	2-4-6 months	12 months	DTPa-HBV-Hib-TT/IPV/Rotavirus	modified 3rd generation ELISA
27^21^^,^^50^	Madhi et al. 2020^50^	NCT02943902	Individual	pcv13 vs pcv10	South Africa	Africa	1 + 1	6 weeks	40 weeks	DTPa-HBV-Hib-TT/IPV/Rotavirus/Measles	in-house ELISA according to the standarised WHO protocol
27^21^^,^^50^	Madhi et al. 2020^50^	NCT02943902	Individual	pcv13 vs pcv10	South Africa	Africa	1 + 1	14 weeks	40 weeks	DTPa-HBV-Hib-TT/IPV/Rotavirus/Measles	in-house ELISA according to the standarised WHO protocol
27^21^^,^^50^	Madhi et al. 2020^50^	NCT02943902	Individual	pcv13 vs pcv10	South Africa	Africa	1 + 1	6–14 weeks	40 weeks	DTPa-HBV-Hib-TT/IPV/Rotavirus/Measles	in-house ELISA according to the standarised WHO protocol
28^92^	Adigweme et al., 2023	NCT03896477	Aggregate	pcv13 vs pcv10	Gambia	Africa	2 + 1	6–8 and 14–16 weeks	9–18 months	DTwP-Hib- HBV/bOPV/Rotavirus	validated ELISA by the WHO Pneumococcal Serology Reference Laboratory

PCV, Pneumococcal conjugate vaccine; DTaP, diphtheria and tetanus toxoids, and acellular pertussis vaccine; DTwP, diphtheria and tetanus toxoids, and whole-cell pertussis vaccine; Hib, *Haemophilus influenzae* type b vaccine; HBV, Hepatitis B vaccine; IPV, Inactivated oral polio vaccine; OPV, Oral polio vaccine; bOPV, Bivalent oral polio vaccine; MenC, Meningococcal C vaccine; NCT, National Clinical Trial; TT, Tetanus toxoid conjugate; NA, Not applicable/not reported; WHO, World Health Organisation; ELISA, Enzyme-linked immunosorbent assay.

a In “Cohort ID” column all relevant publication records are cited; in “Author & Year” column the main study relevant to the analysis are cited.

There were 7 studies comparing PCV10 vs PCV7, 14 studies comparing PCV13 vs PCV7, and 8 studies comparing PCV13 vs PCV10 (Supplementary Fig. S1b). Two cohorts used a 1 + 1 schedule with the first dose administered at either 6- or 14- weeks of age to South African infants and compared PCV13 with PCV10.^50^ Five cohorts used a 2 + 1 prime-boost schedule, while three cohorts used a 3 + 0 schedule. The remaining 20 cohorts tested a 3 + 1 schedule, with most cohorts receiving a primary series at 2-4-6 months (n = 9) and a booster at around 12 months (n = 18). Most cohorts reported or cited types of co-administered vaccines (n = 25) (Table 1). Serotype-specific IgG antibody responses were defined as primary outcomes in all studies. Geometric mean concentrations (GMC) were reported at 28 days post-primary series (n = 29 cohorts), prior to a booster (n = 18 cohorts) and 28 days post-booster (n = 26 cohorts). Individual participant data were available from 25 of 31 (80.6%) cohorts.

Risk of bias assessments for the 28 included studies are summarised in Supplementary Fig. S2. Results of ten studies^33^^,^^35^^,^^38^^,^^40^^,^^53^^,^^57^^,^^58^^,^^67^^,^^69^^,^^71^ were assessed to be at ‘low risk of bias’ across all domains and overall. Two studies^25^^,^^68^ had results judged to be at ‘high risk of bias’ due to problems identified in one domain each: Wysocki 2009^68^ only analysed immunogenicity for a subset of participants and Bryant 2010^25^ did not report whether participants or staff delivering the intervention were blinded to the vaccine received. Lack of information was reported in Bryant 2010^25^ for the analysis, raising concerns on appropriateness of the analysis for the aggregate data obtained from this study. The remaining 16 studies^20^^,^^23^^,^^26^^,^^32^^,^^36^^,^^39^^,^^41^^,^^44^^,^^50^^,^^51^^,^^54^^,^^59^^,^^61^^,^^62^^,^^66^^,^^92^ were judged to have ‘some concerns’ over risk of bias. These concerns predominantly arose because the randomisation process was not described, and/or the study did not report if the participants or staff delivering the vaccines were blinded to which vaccines were given. We did not find any evidence of publication bias by two assessment tools (all p values for Egger's test are >0.05). Full details of our publication bias findings are given in Supplementary material.

**Fig. 2:**
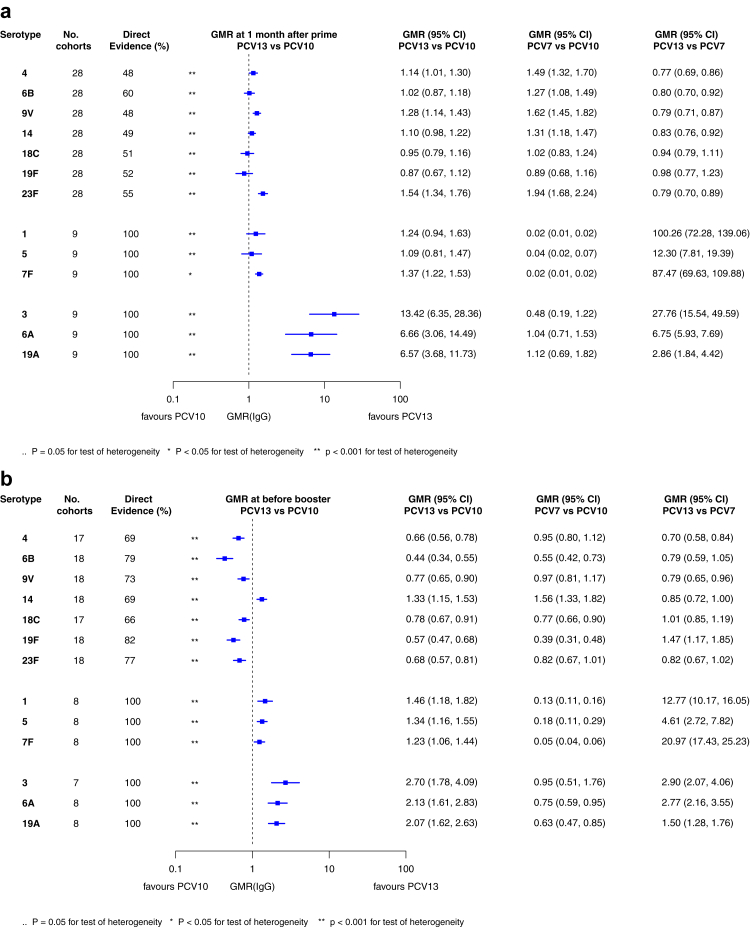

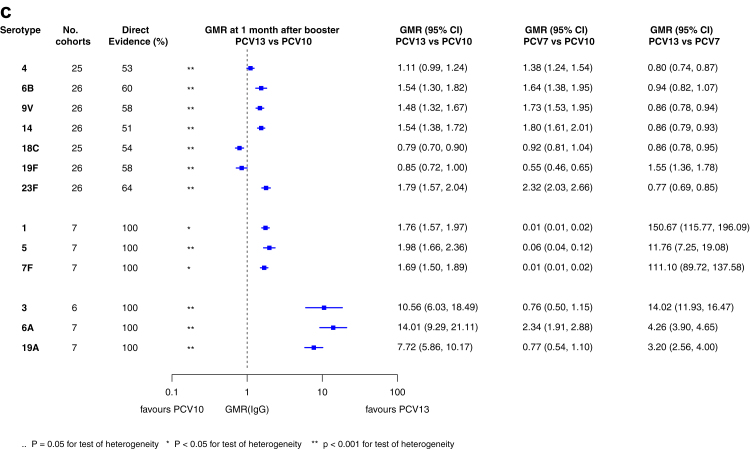
**Geometric mean ratios from meta-analyses of head-to-head studies at a) 28 days post-primary vaccination series, b) pre-booster, and c) 28 days post-booster**. GMR: Geometric mean ratio; PCV: Pneumococcal conjugate vaccine. Each line in the figure shows the output from a network meta-analyses (PCV7 serotypes) or direct meta-analyses (PCV13 but non-PCV7 serotypes). Blue boxes and blue lines show the point estimates and confidence intervals for geometric mean ratios comparing PCV13 vs PCV10. Points to the right of the vertical line are those with higher antibody responses in the PCV13 arm of the study, and points to the left are those with higher antibody responses in the PCV10 arm. The direct evidence column shows the percentage of evidence from studies directly comparing PCV13 vs PCV10 that contributes to the estimates presented in the figure in blue (PCV13 vs PCV10). GMR of PCV13 vs PCV10 for PCV10 and PCV13 serotypes are from a meta-analysis of only head-to-head studies of PCV13 vs PCV10.

Fig. 2 shows the number of study cohorts included in each analysis and the estimated GMR for each serotype and time point, and Supplementary Table S3 summarises the heterogeneity statistics and inconsistency of the network. Substantial heterogeneity and network inconsistency were present for most serotypes at all three time points.

Direct (comparisons between PCV10 and PCV13) and indirect (comparisons of PCV13 vs PCV7 and PCV10 vs PCV7) evidence from 28 cohorts were available for immunogenicity analysis at 28 days post-primary vaccination (Supplementary Fig. S3a). GMRs comparing PCV13 vs PCV10 for any primary series schedule were higher in PCV13 for serotypes 4, 7F, 9V, and 23F at 1 month after primary vaccination series, with 1.14- to 1.54- fold significantly higher IgG responses in PCV13. Additional serotypes contained only in the PCV13 vaccine (3, 6A and 19A) also favoured PCV13 as expected. GMRs were similar for the remaining serotypes (1, 5, 6B, 14, 18C, 19F, Fig. 2a). Within the network meta-analyses comparisons with PCV7, GMRs favoured PCV7 over either PCV13 or PCV10 for serotypes 4, 6B, 9V, 14, and 23F. There was no difference in GMRs for Serotypes 18C and 19F across three vaccines (Fig. 2a). Heterogeneity was observed for all serotypes at the post-primary visit (p-value for heterogeneity <0.05). There were inconsistencies between direct and indirect evidence from the network meta-analysis (p-value for inconsistency <0.05) for serotype 6B, 14, 18C and 19F (Supplementary Table S3).

At the pre-booster time point data were available from 18 cohorts. IgG responses were higher with PCV10 compared with PCV13 for all PCV7 serotypes except for serotype 14, with the point estimates of GMRs comparing PCV13 vs PCV10 ranging from 0.44 to 0.78. IgG responses were higher for PCV13 for serotypes 1, 5 and 7F. GMRs comparing PCV13 vs PCV7 showed higher IgG with PCV7 for serotypes 4, 6B, 9V, 14 and 23F, and higher IgG with PCV13 for serotype 19F (Fig. 2b). Heterogeneity was present for all serotypes (p-value for heterogeneity <0.05) and inconsistencies were present for serotype 18C and 19F at the pre-booster time point (p-value for inconsistency <0.05).

At 28 days post booster, data were available from 26 cohorts. GMRs favoured PCV13 over PCV10 for serotype 6B, 9V, 14 and 23F, and favoured PCV10 over PCV13 for serotype 18C (Fig. 2c). For serotype 1, 5 and 7F, antibody responses were higher in PCV13 compared with PCV10. PCV7 recipients had higher GMCs compared with PCV13 for all PCV7 serotypes except 6B for which there was no difference, and19F, which favoured PCV13. For PCV13-only serotypes (3, 6A and 19A), GMRs favour PCV13 at all three time points. Heterogeneity was significant for all serotypes and there was network inconsistency for serotype 4 (Supplementary Table S3, Supplementary Fig. S3).

To explore potential reasons for the observed heterogeneity, we summarised cohort-level GMRs for each vaccine comparison and present these with concomitant vaccines and vaccine schedules at all three time points in Supplementary Fig. S4–S42. These descriptive analyses revealed a lack of consistency in the direction of study-level estimates within each vaccine comparison, resulting in the significant heterogenicity. There was also no observable pattern in any trial level variable (region, co-administered vaccines, vaccine schedule), from which one might propose a mechanism that would adequately explain this variation in GMRs, although studies which compared vaccines with the same carrier protein seemed to have more consistent estimates. In sensitivity analysis, we restricted to 11 cohorts providing IgG results for all the three time points, and observed similar results (Supplementary Fig. S43). The sensitivity analysis was unable to be stratified by co-administered vaccines due to the limited number of cohorts (less than five) reporting the same co-administered vaccines. Sensitivity analysis stratified by region and vaccine schedule demonstrated reduced heterogeneity for some serotypes and similar patterns compared with main analysis (Supplementary Fig. S44–S46). Additional sensitivity analyses excluding two studies with ‘high risk of bias’ and separately, excluding the study which used a 1 + 1 schedule did not affect the results.

There were 12 studies (15 cohorts) with available individual participant antibody data at both post-primary and prior to the booster dose, allowing serotype-specific estimation of seroefficacy from a total of 5152 participants. Of these 15 cohorts, 6 compared PCV10 vs PCV7, 3 compared PCV13 vs PCV7 and 6 compared PCV13 vs PCV10 (Supplementary Fig. S2b). No issue was found regarding to the integrity of individual participant data.

**Fig. 3:**
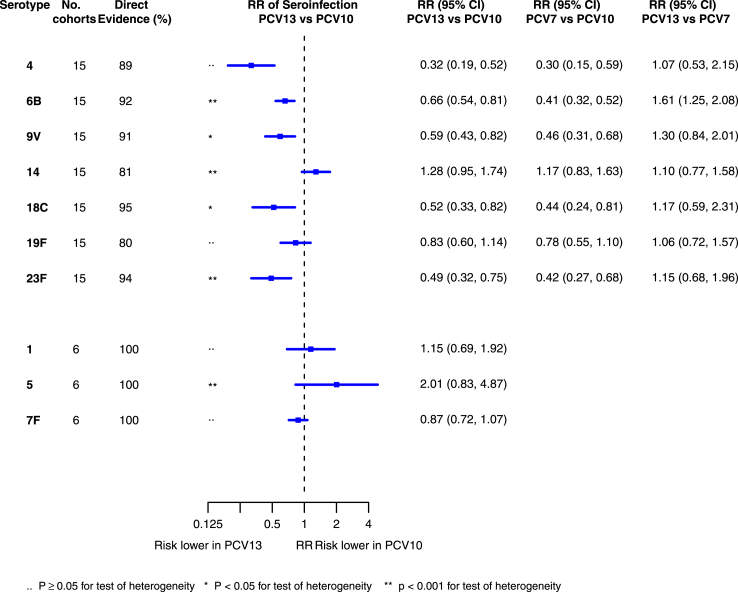
**Relative risk of seroinfection from meta-analyses of head-to-head studies**. RR: Relative risk; PCV: Pneumococcal conjugate vaccine. Each line in the figure shows the output from a network meta-analyses (PCV7 serotypes) or direct meta-analyses (PCV10 serotypes). Blue boxes and blue lines show the point estimates and confidence intervals of relative risk of seroinfection comparing PCV13 vs PCV10. The direct evidence column shows the percentage of evidence from studies directly comparing PCV13 vs PCV10. Results for PCV10 serotypes are from a meta-analysis of only head-to-head studies of PCV13 vs PCV10, therefore estimates of PCV7 vs PCV10 and PCV13 vs PCV7 were not available.

The relative risk of seroinfection from the network meta-analysis for each serotype is summarised in Fig. 3 and a summary of direct and indirect evidence is given in Supplementary Fig. S47. The I^2^ and p value indicate some heterogeneity for all PCV7 serotypes except for serotype 4 and 19F (Supplementary Table S4).

Among PCV7 serotypes, the risk of seroinfection was lower with PCV13 than PCV10 for serotypes 4, 6B, 9V, 18C and 23F, while no difference was seen for serotype 14 and 19F (Fig. 2). The RRs of seroinfection (PCV13 vs PCV10) for PCV7 serotypes ranged from 0.32 (95% CI 0.19, 0.52) for serotype 4 to 1.28 (95% CI 0.95, 1.74) for serotype 14. The direct evidence contributed to around 80%–95% of total evidence, and we found no inconsistency between direct and indirect evidence for all but serotype 19F (p values > 0.05, Supplementary Table S4, Supplementary Fig. S48–S57).

For serotypes 1, 5, and 7F, evidence was summarised from 6 studies directly comparing PCV13 with PCV10. Heterogeneity was observed for serotype 5 and all confidence intervals overlapped 1.0. Comparisons between PCV13 and PCV7 favoured neither vaccine over the other, whereas comparisons between PCV7 and PCV10 favoured PCV7 for serotypes 5, 6B, 9V, 18C, and 23F. Sensitivity analyses of studies conducted in Europe and using 3 + 1 schedule showed similar RRs as estimated from the main analysis (Supplementary Fig. S58 and S59). The seroefficacy analysis results remained consistent after removing one “high risk of bias” study from the analysis.

Supplementary Fig. S60 shows the serotype-specific relationships between immunogenicity (GMRs) and seroefficacy (RRs). Log-GMRs and log-RRs were highly or moderately correlated for all PCV7 serotypes (with weighted Pearson correlation coefficients (*r*) ranging from −0.76 to −0.60, all p < 0.05) except for serotype 14 (*r* = −0.30, p = 0.26).

**Fig. 4:**
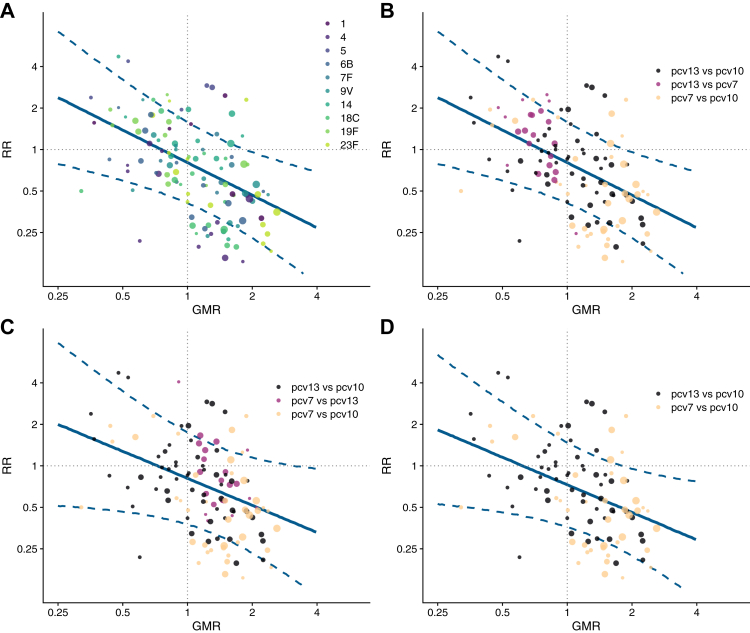
**Overall association between geometric mean ratio and relative risk across all serotypes in PCV10**. RR: Relative risk; GMR: Geometric mean ratio; PCV: Pneumococcal conjugate vaccine. Each point shows results of a serotype specific head-to-head comparison between two vaccines from one study. Solid line shows the relationship between relative risk predicted from the model and geometric mean ratio. Dashed line shows the confidence intervals of predicted relative risk. Reference lines show geometric mean ratio equivalent to one (vertical) and relative risk equivalent to one (horizontal) which represent values associated with no difference between vaccines. Points sizes represent sample size of the trial. Panel A) shows the relationship by 13 serotypes covered by PCV13, B) shows the same data as panel A classified by vaccine comparison groups, C) shows the same data as panel B, however, studies comparing PCV13 vs PCV7 are analysed and displayed as PCV7 vs PCV13 as a sensitivity analysis D) shows a further sensitivity analysis that excludes studies of PCV13 vs PCV7 and only shows studies that compared vaccines from two different manufacturers.

In the combined analysis across all serotypes vaccines that produced the same amount of antibody (GMR = 1) had very similar protection (adjusted RR: 0.80, 95%: CI 0.41–1.58, Fig. 4). The model estimate indicates that for each two-fold increase in antibody response, the risk of seroinfection was halved (GMR of 2.0; RR 0.46, 95% CI 0.23–0.96, Fig. 4a and b). The estimates were stable when estimates of PCV13 vs PCV7 were analysed in reverse as PCV7 vs PCV13 (GMR of 2.0; RR: 0.51, 95% CI 0.23–1.15, Fig. 4c).

When analyses were restricted to comparison between products from different manufacturers the relationship between immunogenicity and seroefficacy remained similar to the main analysis with a confidence interval that incorporates 1.0 (GMR 1.0; RR: 0.73, 95% CI 0.36–1.47) (Fig. 4d).

## Discussion

In our study we used a novel methodology to define seroinfection from immunogenicity data to compare the seroefficacy of pneumococcal conjugate vaccines. The results from our global meta-analysis, provide the first estimates of the comparative protection afforded by different pneumococcal vaccines, and shows that for many serotypes, seroinfection is less common after PCV13 than PCV10, in line with the higher antibody response to PCV13. In addition, we quantify the relationship between the immune response to vaccination and protection against seroinfection, and show that a higher antibody response to vaccination is associated with greater protection from subsequent infection.

The heterogenicity we observed was unexpected. We assumed that if one vaccine is able to induce more antibody than another, then it would do so with some degree of consistency. However, comparisons of the same vaccines in different studies gave widely varying estimates and although we have reported the summary estimates in our meta-analyses, the large degree of between-study heterogeneity in these models means overall estimates are difficult to interpret. In some settings PCV13 performed better yet in others PCV10 was the more immunogenic vaccine. No study-level factor was identified that might explain the variation in estimate. However, only three candidate factors could be considered (location, schedule, and co-administered vaccines) and data on co-administered vaccines was not comprehensive. It is unlikely that differences in assays used would cause this variation as these assays are WHO standardised and only head-to-head comparisons were included.

Of note, comparisons between vaccines from the same manufacturer (PCV13 vs PCV7) were more consistent than comparisons between vaccines from different manufacturers. Immune interference (“bystander effects”) can occur when vaccines with similar components are co-administered,^97^ and this may have different effects for vaccines from different manufacturers. An additional potential confounder that is unmeasured in these studies, is the exposure to circulating serotypes of pneumococcus in each setting, which may also influence the immune response to vaccines. Further investigation into the predictors of vaccine-specific immune responses may be warranted to determine which product is best in diverse settings.

Licensure of new vaccines is based on non-inferiority comparisons with current vaccines and the proportion of antibody responses above the agreed threshold as a minimum requirement. Once a vaccine meets this “at-least-as-good-as” immunogenicity criteria, it has previously not been clear whether exceeding it is of benefit, and the WHO position paper states “*It is unknown whether a lower serotype-specific GMC of antibody indicates less efficacy*”.^6^ We modelled the relationship between seroefficacy (RRs) and immunogenicity (GMRs), to determine whether higher antibody responses were associated with better protection. This regression did not require a meta-analysis to be performed, but instead used individual trial estimates, thereby capitalising on the observed between-study heterogenicity rather than being hindered by it. Our results show that lower protection against subclinical infection does indeed follow from lower antibody production, and that two vaccines that produce a similar level of antibody will provide similar levels of protection, even if they are from different manufacturers.

The implications of these findings are of greatest importance when a new vaccine rollout is being considered. Lower antibody production or lower seroefficacy for one vaccine product does not necessarily imply poor effectiveness against invasive pneumococcal diseases when considering vaccines such as PCV10 and PCV13 which have been shown to be highly effective vaccines in many settings. Instead, lower antibody production would lead to less rapidly observed indirect protection after implementation into a national programme as a smaller proportion of transmission events would be blocked. For serotypes where protective impact has not been observed (serotype 3), new vaccines with substantially higher antibody responses may be needed. A phase II clinical trial of PCV15 compared with PCV13 reported almost twice the antibody level for serotype 3 at 28 days post-primary series for PCV15 (GMR 1.93, 95% CI 1.71, 2.18).^85^ Based on our modelled association between GMR and RR, the relative risk of seroinfection with PCV15 versus PCV13 was estimated to be 0.48. The previously reported vaccine effectiveness for serotype 3 was −27% (95% CI −180, 44) against nasopharyngeal carriage,^98^ and this translates to a point estimate of 40% vaccine effectiveness against carriage of this serotype with PCV15 (VE_(pcv15)_=(1-RR_(pcv15 vs pcv13)_∗(1-VE_(pcv13)_/100%))∗100%).

This evidence of differences in serotype-specific protection can be incorporated into cost-effectiveness models used to compare vaccine products.^99^ Cost-effectiveness studies have highlighted the lack of head-to-head evidence of efficacy for different PCVs, resulting in cost-effectiveness models that ignore serotype-specific differences and assume equivalent efficacy for different PCVs.^100^, ^101^, ^102^ Our study fills this evidence gap and allows researchers and policy-makers to use more accurate vaccine-specific models in decision-making.

There is substantial evidence from pneumococcal challenge studies that participants exposed (“challenged”) with pneumococcus who go on to develop an established carriage infection experience significant increases in antibody post-exposure, whereas those who remain carriage negative do not.^103^, ^104^, ^105^ Cross-sectional carriage studies using nasopharyngeal swabs are susceptible to misclassification bias when the time of sampling is not at the exact time of peak infection resulting in a negative swab. Using seroinfection as an outcome reduces this type of bias as the antibody response to carriage persists for a longer period of time than the carriage event. Nevertheless, misclassification bias can exist if antibody wanes quickly following infection, which may bias the RR estimates to the null.

Seroefficacy analyses need to be restricted to serotypes contained in both vaccines. Comparing a vaccinated cohort to a cohort that is unvaccinated, or who received a vaccine that does not contain the serotype of interest, will result in biased estimates as the immune response after exposure to a pathogen will differ in children whose immune system is primed for that pathogen, when compared with a naïve population. For this reason, we restricted our seroefficacy analysis to shared serotypes between vaccines. Whilst seroinfection is most likely an indicator of nasopharyngeal carriage, it may also represent cases of asymptomatic bacteremia.

In conclusion, we estimated serotype-specific difference in both seroefficacy and immunogenicity between PCV10 and PCV13. Higher IgG antibody levels confer better protection against seroinfection. We recommend incorporating serotype-specific vaccine seroefficacy estimates when modelling cost-effectiveness of future vaccine introductions. In addition, we recommend that the impact of lower geometric mean antibody responses for new vaccines be considered in light of the likely reduced effect on transmission.

## Contributors

MV conceived and designed the study and obtained funding. SF, JM, NR, JH and MV contributed to the methods of the study. NR devised and conducted the search strategy. JM and NP conducted the study selection and assessment of bias. SF and MV obtained the individual participant data and extracted the aggregated data. SF and MV accessed and verified the underlying data. SF wrote the first draft of the manuscript. All authors interpreted the data and contributed to the writing of the final version of the manuscript. The corresponding author had full access to all the data in the study and had final responsibility for the decision to submit for publication.

## Data sharing statement

This publication is based on research using data from data contributor Pfizer that was made available through Vivli, Inc, and data contributor GSK that was made available through clinicalstudydatarequest.com. Vivli and other third parties have not contributed to or approved, and are not in any way responsible for, the contents of this publication. Data were made available for a limited period of time to conduct these analyses. The authors do not have continuing access to the datasets.

## Declaration of interests

AJP is Chair of the UK DHSC Joint on Vaccination & Immunisation and was a member of the Strategic Advisory Group of Experts on Immunization to the WHO (World Health Organization) until 2022. AJP, MV and SF are contributors to COVID-19 vaccine intellectual property licensed by Oxford University Innovation to AstraZeneca. SM reports grants to his institution from the Bill and Melinda Gates Foundation, Pfizer and GSK. KM is an investigator on grants from MSD and Pfizer and reports grants to his institution from the Bill and Melinda Gates Foundation and the World Health Organization. MJ reports grants to his institution from Bill & Melinda Gates Foundation, Gavi the Vaccine Alliance, NIHR, RCUK, European Commission, and Wellcome Trust. All other authors declare no competing interests. The views expressed in this article do not necessarily represent the views of the DHSC, JCVI, NIHR, or WHO.
