# Supplementary material for: Immunogenicity and seroefficacy of 10-valent and 13-valent pneumococcal conjugate vaccines: a systematic review and network meta-analysis of individual participant data
Source: eClinicalMedicine. 2023 Jul 1;61:102073. doi: 10.1016/j.eclinm.2023.102073 (PMC10328810; doi:10.1016/j.eclinm.2023.102073)
Supplement: Supplementary Figs. S1–S60 and Tables S1–S4 [file mmc1.docx]

**Supplementary file**

Table of Contents

[Search strategy - Databases 4](#_Toc136541393)

[PCVs in systematic review 4](#_Toc136541395)

[Data retrieval 4](#_Toc136541396)

[Assessment of Publication Bias 5](#_Toc136541397)

[Sensitivity Analysis 8](#_Toc136541398)

[References 8](#_Toc136541399)

[Supplementary Table 1. Full search terms for databases. 10](#_Toc132288854)

[Supplementary Table 2. Summary of eligible studies excluded from network meta-analysis due to either no closed loop or data unavailable. 16](#_Toc132288855)

[Supplementary Table 3. Summary of the statistical heterogeneity and incoherence for immunogenicity analyses shown in Figure 2 17](#_Toc132288856)

[Supplementary Table 4. Summary on assessment of statistical heterogeneity and incoherence for seroefficacy analysis shown in Figure 3 21](#_Toc132288857)

[Supplementary Figure 1. Network of studies included for a) all eligible cohorts, b) immunogenicity analysis cohorts and c) seroefficacy analysis cohorts 23](#_Toc132288858)

[Supplementary Figure 2. Assessment of risk of bias for included studies 24](#_Toc132288859)

[Supplementary Figure 3. Direct and indirect evidence on geometric mean ratios comparing PCV13 vs PCV10 for serotypes in PCV7 at a) 28 days post-primary vaccination series, b) pre-booster, and c) 28 days post-booster 25](#_Toc132288860)

[Supplementary Figure 4. Trial level geometric mean ratios for serotype 4 post-primary vaccination series. 28](#_Toc132288861)

[Supplementary Figure 5. Trial level geometric mean ratios for serotype 6B post-primary vaccination series. 29](#_Toc132288862)

[Supplementary Figure 6. Trial level geometric mean ratios for serotype 9V post-primary vaccination series. 30](#_Toc132288863)

[Supplementary Figure 7. Trial level geometric mean ratios for serotype 14 post-primary vaccination series. 31](#_Toc132288864)

[Supplementary Figure 8. Trial level geometric mean ratios for serotype 18C post-primary vaccination series. 32](#_Toc132288865)

[Supplementary Figure 9. Trial level geometric mean ratios for serotype 19F post-primary vaccination series. 33](#_Toc132288866)

[Supplementary Figure 10. Trial level geometric mean ratios for serotype 23F post-primary vaccination series. 34](#_Toc132288867)

[Supplementary Figure 11. Trial level geometric mean ratios for serotype 1 post-primary vaccination series. 35](#_Toc132288868)

[Supplementary Figure 12. Trial level geometric mean ratios for serotype 5 post-primary vaccination series. 36](#_Toc132288869)

[Supplementary Figure 13. Trial level geometric mean ratios for serotype 7F post-primary vaccination series. 37](#_Toc132288870)

[Supplementary Figure 14. Trial level geometric mean ratios for serotype 3 post-primary vaccination series. 38](#_Toc132288871)

[Supplementary Figure 15. Trial level geometric mean ratios for serotype 6A post-primary vaccination series. 39](#_Toc132288872)

[Supplementary Figure 16. Trial level geometric mean ratios for serotype 19A post-primary vaccination series. 40](#_Toc132288873)

[Supplementary Figure 17. Trial level geometric mean ratios for serotype 4 pre-booster. 41](#_Toc132288874)

[Supplementary Figure 18. Trial level geometric mean ratios for serotype 6B pre-booster. 42](#_Toc132288875)

[Supplementary Figure 19. Trial level geometric mean ratios for serotype 9V pre-booster. 43](#_Toc132288876)

[Supplementary Figure 20. Trial level geometric mean ratios for serotype 14 pre-booster. 44](#_Toc132288877)

[Supplementary Figure 21. Trial level geometric mean ratios for serotype 18C pre-booster. 45](#_Toc132288878)

[Supplementary Figure 22. Trial level geometric mean ratios for serotype 19F pre-booster. 46](#_Toc132288879)

[Supplementary Figure 23. Trial level geometric mean ratios for serotype 23F pre-booster. 47](#_Toc132288880)

[Supplementary Figure 24. Trial level geometric mean ratios for serotype 1 pre-booster. 48](#_Toc132288881)

[Supplementary Figure 25. Trial level geometric mean ratios for serotype 5 pre-booster. 49](#_Toc132288882)

[Supplementary Figure 26. Trial level geometric mean ratios for serotype 7F pre-booster. 50](#_Toc132288883)

[Supplementary Figure 27. Trial level geometric mean ratios for serotype 3 pre-booster. 51](#_Toc132288884)

[Supplementary Figure 28. Trial level geometric mean ratios for serotype 6A pre-booster. 52](#_Toc132288885)

[Supplementary Figure 29. Trial level geometric mean ratios for serotype 19A pre-booster. 53](#_Toc132288886)

[Supplementary Figure 30. Trial level geometric mean ratios for serotype 4 post-booster. 54](#_Toc132288887)

[Supplementary Figure 31. Trial level geometric mean ratios for serotype 6B post-booster. 55](#_Toc132288888)

[Supplementary Figure 32. Trial level geometric mean ratios for serotype 9V post-booster. 56](#_Toc132288889)

[Supplementary Figure 33. Trial level geometric mean ratios for serotype 14 post-booster. 57](#_Toc132288890)

[Supplementary Figure 34. Trial level geometric mean ratios for serotype 18C post-booster. 58](#_Toc132288891)

[Supplementary Figure 35. Trial level geometric mean ratios for serotype 19F post-booster. 59](#_Toc132288892)

[Supplementary Figure 36. Trial level geometric mean ratios for serotype 23F post-booster. 60](#_Toc132288893)

[Supplementary Figure 37. Trial level geometric mean ratios for serotype 1 post-booster. 61](#_Toc132288894)

[Supplementary Figure 38. Trial level geometric mean ratios for serotype 5 post-booster. 62](#_Toc132288895)

[Supplementary Figure 39. Trial level geometric mean ratios for serotype 7F post-booster. 63](#_Toc132288896)

[Supplementary Figure 40. Trial level geometric mean ratios for serotype 3 post-booster. 64](#_Toc132288897)

[Supplementary Figure 41. Trial level geometric mean ratios for serotype 6A post-booster. 65](#_Toc132288898)

[Supplementary Figure 42. Trial level geometric mean ratios for serotype 19A post-booster. 66](#_Toc132288899)

[Supplementary Figure 43. Geometric mean ratios from sensitivity analyses restricted to studies providing data for all three time points a) post-primary vaccination series, b) pre-boost, and c) post-boost 67](#_Toc132288900)

[Supplementary Figure 44. Geometric mean ratios from sensitivity analysis of studies conducted in Europe at a) post-primary vaccination series, b) pre-boost, and c) post-boost 70](#_Toc132288901)

[Supplementary Figure 45. Geometric mean ratios from sensitivity analyses of studies conducted in Asia: a) post-primary vaccination series and b) post-boost 73](#_Toc132288902)

[Supplementary Figure 46. Geometric mean ratios from sensitivity analyses of studies that used a 3+1 schedule at a) post-primary vaccination series, b) pre-boost, and c) post-boost 75](#_Toc132288903)

[Supplementary Figure 47. Direct and indirect evidence on relative risk comparing PCV13 vs PCV10 for serotypes in PCV7 (4, 6B, 9V, 14, 18C, 19F and 23F) 78](#_Toc132288904)

[Supplementary Figure 48. Trial level relative risk for serotype 4. 79](#_Toc132288905)

[Supplementary Figure 49. Trial level relative risk for serotype 6B. 80](#_Toc132288906)

[Supplementary Figure 50. Trial level relative risk for serotype 9V. 81](#_Toc132288907)

[Supplementary Figure 51. Trial level relative risk for serotype 14. 82](#_Toc132288908)

[Supplementary Figure 52. Trial level relative risk for serotype 18C. 83](#_Toc132288909)

[Supplementary Figure 53. Trial level relative risk for serotype 4. 84](#_Toc132288910)

[Supplementary Figure 54. Trial level relative risk for serotype 23F. 85](#_Toc132288911)

[Supplementary Figure 55. Trial level relative risk for serotype 1. 86](#_Toc132288912)

[Supplementary Figure 56. Trial level relative risk for serotype 5. 87](#_Toc132288913)

[Supplementary Figure 57. Trial level relative risk for serotype 7F. 88](#_Toc132288914)

[Supplementary Figure 58. Sensitivity analysis on relative risk of seroinfection for studies conducted in Europe. 89](#_Toc132288915)

[Supplementary Figure 59. Sensitivity analysis of relative risk of seroinfection for studies using a 3+1 schedule. 90](#_Toc132288916)

[Supplementary Figure 60. Study level association between geometric mean ratio and relative risk for each serotype in PCV7 92](#_Toc132288917)

# Search strategy - Databases

The databases searched were Cochrane Database of Systematic Reviews & Cochrane Central Register of Controlled Trials (Cochrane Library, Wiley)[Issue 2 of 12, July 2023 and issue 1 of 12 January 2023 respectively], Embase (OvidSP)[1974-present], Global Health (OvidSP)[1973 to 2022 Week 29] and Medline (OvidSP)[1946-present]. The trial registers searched were ClinicalTrials.gov (https://clinicaltrials.gov/) and WHO International Clinical Trials Registry Platform (https://trialsearch.who.int/). The search comprised of title/abstract keywords and subject headings for pneumococcal vaccines and children. A methodological search filter for randomised controlled trials taken from the Cochrane Handbook was used to limit to RCTs.^1,2^ Pharmaceutical company websites (GSK and Pfizer) were also hand searched for relevant studies. A full list of search terms for each database is summarised in Supplementary Table 1. References were exported to Endnote 20 for de-duplication.

# PCVs in systematic review

1. 7-valent pneumococcal conjugate vaccine (PCV7: Prevnar, Pfizer), containing serotypes 4, 6B, 9V, 14, 18C, 19F, and 23F, each conjugated to diphtheria cross-reacting material (CRM).
2. 13-valent pneumococcal conjugate vaccine (PCV13: Prevenar 13, Pfizer), containing serotypes 1, 3, 4, 5, 6A, 6B, 7F, 9V, 14, 18C, 19A, 19F, and 23F, each conjugated to diphtheria cross-reacting material (CRM).
3. 10-valent pneumococcal conjugate vaccine (PCV10: Synflorix, GlaxoSmithKline), containing serotypes 1, 4, 5, 6B, 7F, 9V, 14, 18C, 19F, and 23F, conjugated to non-typeable Haemophilus influenzae protein D, for 8 serotypes, or tetanus or diphtheria protein (serotypes 18C and 19F respectively).

# Data retrieval

The following study-level data were extracted from trial registries/published studies:

- trial registration number/study identifier
- study country
- PCV vaccination schedule (e.g. 2+1, 3+1, 3+0)

Individual participant level data were retrieved if available for variables below.

- vaccines administered (both study vaccines and vaccines administered concomitantly as part of the routine immunisation schedule)
- vaccination dates
- details of laboratory assays conducted, including where assays were run, units of measurement, and the lower limit of quantification
- participants’ age at enrolment
- participants’ sex
- serotype-specific anti-pneumococcal IgG measured by ELISA at all time-points

# Assessment of Publication Bias

We evaluated the risk of publication bias utilising two approach. Firstly, we used Tool for assessing Risk Of Bias due to Missing Evidence in a synthesis (ROB-ME, <https://www.riskofbias.info/welcome/rob-me-tool>), which provides a systematic approach to evaluating missing evidence across the review and in a synthesis. Secondly, we constructed “comparison-adjusted” funnel plots, which has been extended from funnel plot into network meta-analysis considering effects for different comparison.^3^ Egger’s test was carried out to evaluate the funnel plot symmetry.

The risk of bias judgement was considered low are by ROB-ME. For 7 serotypes included in all PCVs, p-values testing (Egger test) for funnel plot asymmetry are above 0.05 (Figures shown as below). Overall, we did not find any evidence of publication bias by two approach.


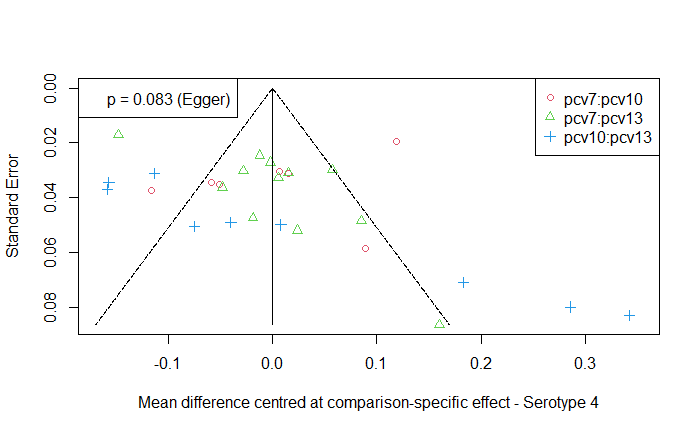


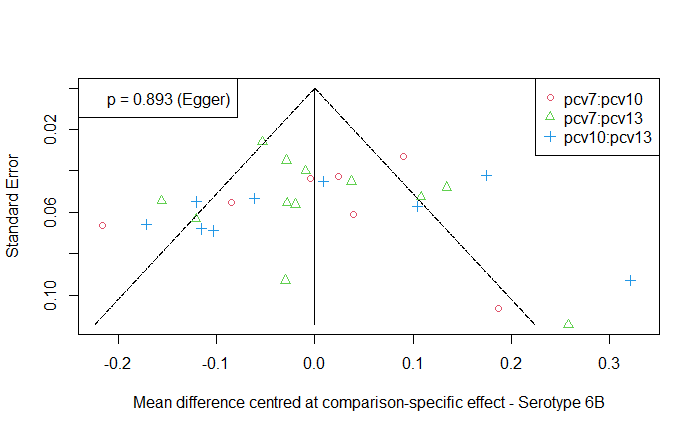

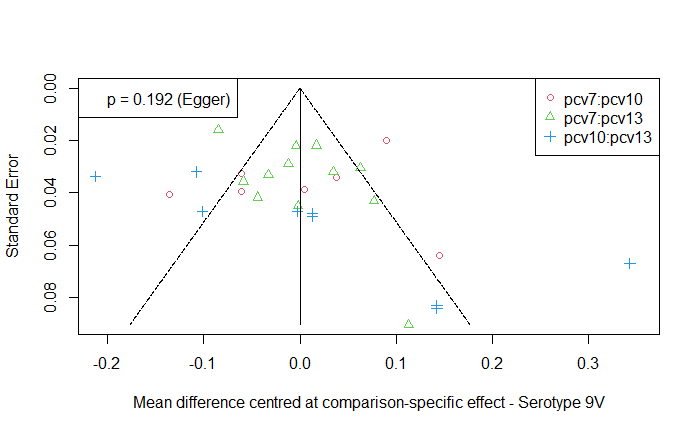

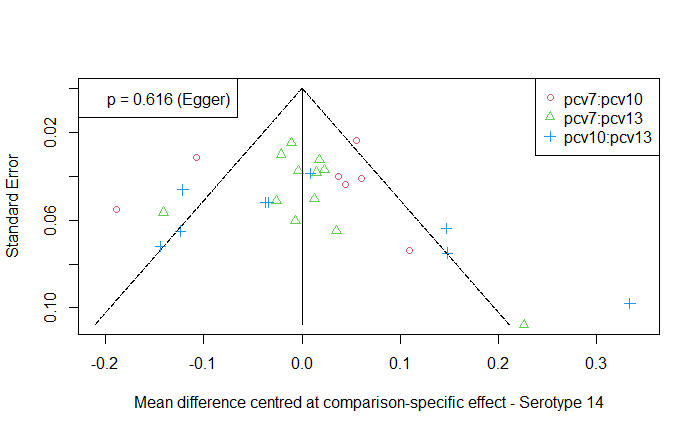


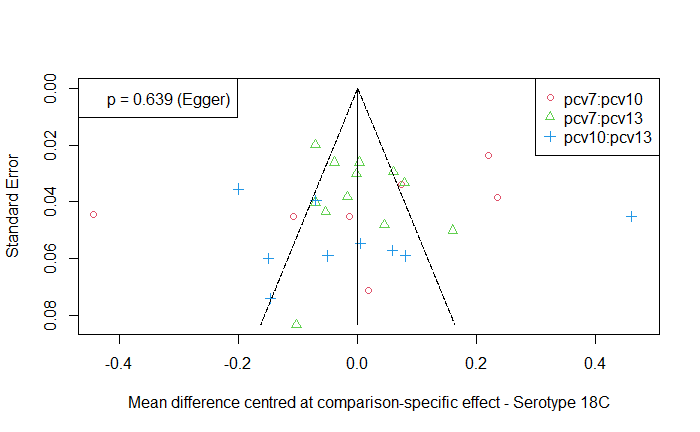


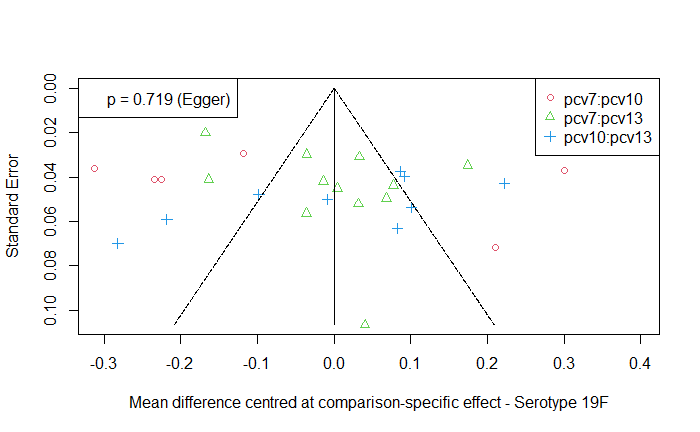

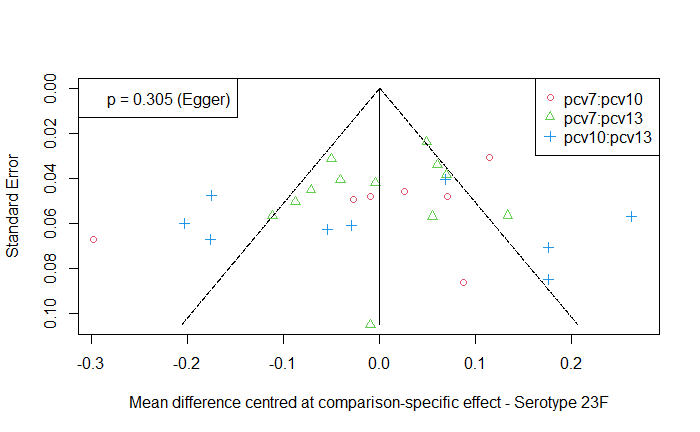


# Sensitivity Analysis

Sensitivity analyses for immunogenicity results were conducted by restricting analyses to only those studies providing data for all three time points of interest. We conducted sensitivity analyses on immunogenicity and seroefficacy. Sensitivity analyses were conducted stratified by study regions and dosing schedules as at least five cohorts were eligible to be included in the network meta-analysis for these factors, and a network could be formed with closed loops to enable use of indirect evidence.

# References

1. Lefebvre C, Glanville J, Briscoe S, Littlewood A, Marshall C, Metzendorf M-I, Noel-Storr A, Rader T, Shokraneh F, Thomas J, Wieland LS. Technical Supplement to Chapter 4: Searching for and selecting studies.

2. Higgins JPT, Thomas J, Chandler J, Cumpston MS, Li T, Page MJ, Welch VA (eds). Cochrane Handbook for Systematic Reviews of Interventions Version 6.2 (updated February 2021). Cochrane, 2021. Available from: [www.training.cochrane.org/handbook](../eClinicalMedicine_R2/www.training.cochrane.org/handbook).

3. Chaimani A, Higgins JP, Mavridis D, Spyridonos P, Salanti G. Graphical tools for network meta-analysis in STATA. *PLoS One* 2013; **8**(10): e76654.

4. A Study in Children With Different Formulations of GSK Biologicals' 11 Valent Pneumococcal Conjugate Vaccine. Available from: <https://clinicaltrials.gov/ct2/show/NCT00169481>. Last accessed on 09 Nov 2022.

5. Dagan R, Melamed R, Muallem M, et al. Reduction of nasopharyngeal carriage of pneumococci during the second year of life by a heptavalent conjugate pneumococcal vaccine. *Journal of Infectious Diseases* 1996; **174**(6): 1271-8.

6. Greenberg D, Hoover PA, Vesikari T, et al. Safety and immunogenicity of 15-valent pneumococcal conjugate vaccine (PCV15) in healthy infants. *Vaccine* 2018; **36**(45): 6883-91.

7. Rupp R, Hurley D, Grayson S, et al. A dose ranging study of 2 different formulations of 15-valent pneumococcal conjugate vaccine (PCV15) in healthy infants. *Human vaccines & Immunotherapeutics* 2019; **15**(3): 549-59.

8. Thisyakorn U, Chokephaibulkit K, Kosalaraksa P, Benjaponpitak S, Pancharoen C, Chuenkitmongkol S. Immunogenicity and safety of 23-valent pneumococcal polysaccharide vaccine as a booster dose in 12- to 18-month-old children primed with 3 doses of 7-valent pneumococcal conjugate vaccine. *Human vaccines & Immunotherapeutics* 2014; **10**(7): 1859-65.

9. Martinez CPD, Linares-Perez N, Toledo-Romani ME, et al. Safety and immunogenicity of the Cuban heptavalent pneumococcal conjugate vaccine in healthy infants. Results from a double-blind randomized control trial Phase I. *Vaccine* 2018; **36**(32 Pt B): 4944-51.

10. Platt HL, Greenberg D, Tapiero B, et al. A Phase II Trial of Safety, Tolerability and Immunogenicity of V114, a 15-Valent Pneumococcal Conjugate Vaccine, Compared With 13-Valent Pneumococcal Conjugate Vaccine in Healthy Infants. *Pediatr Infect Dis J* 2020; **39**(8): 763-70.

11. Bili A, Dobson S, Quinones J, et al. A Phase 3, Multicenter, Randomized, Double-blind Study to Evaluate the Interchangeability of V114 and Prevnar 13TM with Respect to Safety, Tolerability, and Immunogenicity in Healthy Infants (PNEU-DIRECTION). *Open Forum Infectious Diseases* 2021; **8**(SUPPL 1): S684.

12. Bili A, Dobson S, Quinones J, et al. A phase 3, multicenter, randomized, double-blind study to evaluate the interchangeability of V114, a 15-valent pneumococcal conjugate vaccine, and PCV13 with respect to safety, tolerability, and immunogenicity in healthy infants (PNEU-DIRECTION). *Vaccine* 2023; **41**(3): 657-65.

13. Shin J, Teeratakulpisarn J, Puthanakit T, et al. Immunogenicity and safety of a 12-valent pneumococcal conjugate vaccine in infants aged 6–10 weeks: A randomized double-blind active-controlled trial. *Korean Journal of Pediatrics* 2020; **63**(7): 265‐71.

14. Chen JJ, Yuan L, Huang Z, et al. Safety and immunogenicity of a new 13-valent pneumococcal conjugate vaccine versus a licensed 7-valent pneumococcal conjugate vaccine: a study protocol of a randomised non-inferiority trial in China. *BMJ Open* 2016; **6**(10): e012488.

15. Zhao Y, Li G, Xia S, et al. Immunogenicity and Safety of a Novel 13-Valent Pneumococcal Vaccine in Healthy Chinese Infants and Toddlers. *Front Microbiol* 2022; **13**: 870973.

16. Senders S, Klein NP, Lamberth E, et al. Safety and Immunogenicity of a 20-valent Pneumococcal Conjugate Vaccine in Healthy Infants in the United States. *Pediatr Infect Dis J* 2021; **40**(10): 944-51.

17. Senders S, Klein NP, Lamberth E, et al. Safety and Immunogenicity of a 20-Valent Pneumococcal Conjugate Vaccine (PCV20) in Healthy Infants in the United States. *Open Forum Infectious Diseases* 2020; **7**(SUPPL 1): S637.

18. Banniettis N, Wysocki J, Szenborn L, et al. Phase 3 Study to Evaluate the Safety, Tolerability, and Immunogenicity of Catch-up Vaccination Regimens of V114 in Healthy Infants, Children, and Adolescents (PNEU-PLAN). *Open Forum Infectious Diseases* 2021; **8**(SUPPL 1): S678.

19. Banniettis N, Wysocki J, Szenborn L, et al. A phase III, multicenter, randomized, double-blind, active comparator-controlled study to evaluate the safety, tolerability, and immunogenicity of catch-up vaccination regimens of V114, a 15-valent pneumococcal conjugate vaccine, in healthy infants, children, and adolescents (PNEU-PLAN). *Vaccine* 2022; **40**(44): 6315-25.

20. Lupinacci R, Rupp R, Wittawatmongkol O, et al. A PHASE 3, MULTICENTER, RANDOMISED, DOUBLEBLIND, ACTIVE-COMPARATOR-CONTROLLED STUDY TO EVALUATE THE SAFETY, TOLERABILITY, AND IMMUNOGENICITY OF A 4-DOSE REGIMEN OF V114 IN HEALTHY INFANTS (PNEU-PED). *Archives of Disease in Childhood* 2022; **107**(Supplement 2): A204.

21. Lupinacci R, Rupp R, Wittawatmongkol O, et al. A phase 3, multicenter, randomized, double-blind, active-comparator-controlled study to evaluate the safety, tolerability, and immunogenicity of a 4-dose regimen of V114, a 15-valent pneumococcal conjugate vaccine, in healthy infants (PNEU-PED). *Vaccine* 2023; **41**(5): 1142-52.

22. Martinon-Torres F, Gimenez-Sanchez F, Gurtman A, et al. 13-valent pneumococcal conjugate vaccine given with meningococcal C-tetanus toxoid conjugate and other routine pediatric vaccinations: immunogenicity and safety. *Pediatric Infectious Disease Journal* 2012; **31**(4): 392-9.

23. Vanderkooi OG, Scheifele DW, Girgenti D, et al. Safety and immunogenicity of a 13-valent pneumococcal conjugate vaccine in healthy infants and toddlers given with routine pediatric vaccinations in Canada. *Pediatric Infectious Disease Journal* 2012; **31**(1): 72-7.

24. De Los Santos AM, Rodriguez-Weber MA, Sanchez-Marquez P, et al. Immunogenicity of a 2 + 1 infant vaccination series with 13-valent pneumococcal conjugate vaccine (PCV13) followed by pneumococcal non- typeable haemophilus influenzae protein d conjugate vaccine (PHID-CV): A randomized trial exploring interchangeability of PCVS. *Open Forum Infectious Diseases* 2017; **4 (Supplement 1)**: S538-S9.

25. Diez-Domingo J, Gurtman A, Bernaola E, et al. Evaluation of 13-valent pneumococcal conjugate vaccine and concomitant meningococcal group C conjugate vaccine in healthy infants and toddlers in Spain. *Vaccine* 2013; **31**(46): 5486-94.

26. Clarke E, Bashorun AO, Okoye M, et al. Safety and immunogenicity of a novel 10-valent pneumococcal conjugate vaccine candidate in adults, toddlers, and infants in The Gambia-Results of a phase 1/2 randomized, double-blinded, controlled trial. *Vaccine* 2020; **38**(2): 399-410.

27. Clarke E, Bashorun A, Adigweme I, et al. Immunogenicity and safety of a novel ten-valent pneumococcal conjugate vaccine in healthy infants in The Gambia: a phase 3, randomised, double-blind, non-inferiority trial. *Lancet Infect Dis* 2021; **21**(6): 834-46.

Supplementary Table 1. Full search terms for databases.

| **Medline (Ovid MEDLINE® Epub Ahead of Print, In-Process & Other Non-Indexed Citations, Ovid MEDLINE® Daily and Ovid MEDLINE®) 1946 to present** | |
| --- | --- |
| Streptococcus pneumoniae/ | |
| (pneumococc* or s pneumoniae or strep* pneumoniae or strep* p).ti,ab. | |
| 1 or 2 | |
| vaccines/ or bacterial vaccines/ or streptococcal vaccines/ or Vaccines, Conjugate/ | |
| (vaccin* or immuni?ation? or immuni?e? or inoculat* or conjugate).ti. | |
| (conjugate adj2 vaccin*).ti,ab. | |
| (7 valent or 7valent or seven valent or heptavalent or hepta-valent).ti,ab. | |
| (9 valent or 9valent or nine valent or nonavalent or nona-valent).ti,ab. | |
| (10 valent or 10valent or ten valent or decavalent or deca-valent).ti,ab. | |
| (13 valent or 13valent or thirteen valent).ti,ab. | |
| 4 or 5 or 6 or 7 or 8 or 9 or 10 | |
| 3 and 11 | |
| exp Pneumococcal Vaccines/ | |
| ((pneumococc* or s pneumoniae or strep* pneumoniae or strep* p) adj5 (vaccin* or immuni?ation? or immuni?e? or inoculat* or conjugate)).ti,ab. | |
| (pcv7 or pcv 7 or pncrm* or pnccrm* or 7vpnc or 7vcrm).ti,ab. | |
| (pcv9 or pcv 9).ti,ab. | |
| (pcv10 or pcv 10 or phidcv or phid cv).ti,ab. | |
| (pcv13 or pcv 13 or 13vcrm).ti,ab. | |
| (pneumovax or pneumopur or streptopur or streptorix or prevnar or prevenar or synflorix or gsk 1024850a or gsk1024850a).ti,ab. | |
| 12 or 13 or 14 or 15 or 16 or 17 or 18 or 19 | |
| exp child/ or infant/ | |
| (child* or infan* or baby or babies or toddler? or preschool* or pre-school* or p?ediatric?).ti,ab. | |
| 21 or 22 | |
| 20 and 23 | |
| randomized controlled trial.pt. | |
| controlled clinical trial.pt. | |
| randomized.ab. | |
| placebo.ab. | |
| drug therapy.fs. | |
| randomly.ab. | |
| trial.ab. | |
| groups.ab. | |
| 25 or 26 or 27 or 28 or 29 or 30 or 31 or 32 | |
| exp animals/ not humans.sh. | |
| 33 not 34 | |
| 24 and 35 | |
| limit 24 to ("reviews (maximizes specificity)" or "systematic review") | |
| 36 or 37 | |
| (2019* or 2020* or 2021* or 2022*).ed,ez,yr. | |
| 38 and 39 | |
| **Embase 1974 to present** | |
| Streptococcus pneumoniae/ | |
| (pneumococc* or s pneumoniae or strep* pneumoniae or strep* p).ti,ab. | |
| 1 or 2 | |
| vaccine/ or bacterial vaccine/ or streptococcus vaccine/ | |
| (vaccin* or immuni?ation? or immuni?e? or inoculat* or conjugate).ti. | |
| (conjugate adj2 vaccin*).ti,ab. | |
| (7 valent or 7valent or seven valent or heptavalent or hepta-valent).ti,ab. | |
| (9 valent or 9valent or nine valent or nonavalent or nona-valent).ti,ab. | |
| (10 valent or 10valent or ten valent or decavalent or deca-valent).ti,ab. | |
| (13 valent or 13valent or thirteen valent).ti,ab. | |
| 4 or 5 or 6 or 7 or 8 or 9 or 10 | |
| 3 and 11 | |
| pneumococcus vaccine/ | |
| ((pneumococc* or s pneumoniae or strep* pneumoniae or strep* p) adj5 (vaccin* or immuni?ation? or immuni?e? or inoculat* or conjugate)).ti,ab. | |
| (pcv7 or pcv 7 or pncrm* or pnccrm* or 7vpnc or 7vcrm).ti,ab. | |
| (pcv9 or pcv 9).ti,ab. | |
| (pcv10 or pcv 10 or phidcv or phid cv).ti,ab. | |
| (pcv13 or pcv 13 or 13vcrm).ti,ab. | |
| (pneumovax or pneumopur or streptopur or streptorix or prevnar or prevenar or synflorix or gsk 1024850a or gsk1024850a).ti,ab. | |
| 12 or 13 or 14 or 15 or 16 or 17 or 18 or 19 | |
| exp child/ | |
| (child* or infan* or baby or babies or toddler? or preschool* or pre-school* or p?ediatric?).ti,ab. | |
| 21 or 22 | |
| 20 and 23 | |
| randomized controlled trial/ | |
| single blind procedure/ or double blind procedure/ | |
| crossover procedure/ | |
| random*.tw. | |
| (((singl* or doubl*) adj (blind* or mask*)) or crossover or cross over or factorial* or latin square or assign* or allocat* or volunteer*).ti,ab. | |
| 25 or 26 or 27 or 28 or 29 | |
| (exp animals/ or nonhuman/) not human/ | |
| 30 not 31 | |
| 24 and 32 | |
| limit 24 to ("systematic review" or "reviews (maximizes specificity)") | |
| 33 or 34 | |
| (2019* or 2020* or 2021* or 2022*).yr,dd,dc. | |
| 35 and 36 | |
| **Global Health <1973 to 2022 Week 29>** | |
| streptococcus pneumoniae/ | |
| (pneumococc* or s pneumoniae or strep* pneumoniae or strep* p).ti,ab. | |
| 1 or 2 | |
| vaccines/ or conjugate vaccines/ | |
| (vaccin* or immuni?ation? or immuni?e? or inoculat* or conjugate).ti. | |
| (conjugate adj2 vaccin*).ti,ab. | |
| (7 valent or 7valent or seven valent or heptavalent or hepta-valent).ti,ab. | |
| (9 valent or 9valent or nine valent or nonavalent or nona-valent).ti,ab. | |
| (10 valent or 10valent or ten valent or decavalent or deca-valent).ti,ab. | |
| (13 valent or 13valent or thirteen valent).ti,ab. | |
| 4 or 5 or 6 or 7 or 8 or 9 or 10 | |
| 3 and 11 | |
| ((pneumococc* or s pneumoniae or strep* pneumoniae or strep* p) adj5 (vaccin* or immuni?ation? or immuni?e? or inoculat* or conjugate)).ti,ab. | |
| (pcv7 or pcv 7 or pncrm* or pnccrm* or 7vpnc or 7vcrm).ti,ab. | |
| (pcv9 or pcv 9).ti,ab. | |
| (pcv10 or pcv 10 or phidcv or phid cv).ti,ab. | |
| (pcv13 or pcv 13 or 13vcrm).ti,ab. | |
| (pneumovax or pneumopur or streptopur or streptorix or prevnar or prevenar or synflorix or gsk 1024850a or gsk1024850a).ti,ab. | |
| 12 or 13 or 14 or 15 or 16 or 17 or 18 | |
| exp children/ or infants/ | |
| (child* or infan* or baby or babies or toddler? or preschool* or pre-school* or p?ediatric?).ti,ab. | |
| 20 or 21 | |
| 19 and 22 | |
| (random* or blind* or allocat* or assign* or trial* or placebo* or crossover* or cross-over*).mp. | |
| 23 and 24 | |
| (2019* or 2020* or 2021* or 2022*).yr. | |
| 25 and 26 | |
| **ClinicalTrials.gov – 1/6/2019** | |
| (pneumococcal OR pneumococcus OR "streptococcus pneumoniae" OR "streptococcal pneumoniae" OR "streptococcus p" OR "streptococcal p") AND (vaccine OR vaccines OR vaccination OR immunisation OR immunization OR immunise OR immunisation OR inoculate) \| Child | |
| (pneumococcal OR pneumococcus OR "streptococcus pneumoniae" OR "streptococcal pneumoniae" OR "streptococcus p" OR "sterptococcal p") AND (conjugate OR valent) \| Child | |
| pcv7 or pcv 7 or pncrm or pnccrm or 7vpnc or 7vcrm or pcv9 or pcv 9 or pcv10 or pcv 10 or phidcv or phid cv or pcv13 or pcv 13 or 13vcrm | |
| pneumovax or pneumopur or streptopur or streptorix or prevnar or prevenar or synflorix or gsk 1024850a or gsk1024850a \| Child | |
| **ClinicalTrials.gov - 2022 - Added since 01/06/2019** | |
| Other terms=(pneumococcal OR pneumococcus OR "streptococcus pneumoniae" OR "streptococcal pneumoniae" OR "streptococcus p" OR "streptococcal p") AND (vaccine OR vaccines OR vaccination OR immunisation OR immunization OR immunise OR immunisation OR inoculate) \| Child \| First posted from 06/01/2019 to 01/01/2024 | |
| Title=(pneumococcal OR pneumococcus OR "streptococcus pneumoniae" OR "streptococcal pneumoniae" OR "streptococcus p" OR "streptococcal p") AND (vaccine OR vaccines OR vaccination OR immunisation OR immunization OR immunise OR immunisation OR inoculate) \| Child \| First posted from 06/01/2019 to 01/01/2024 | |
| Condition=(pneumococcal OR pneumococcus OR "streptococcus pneumoniae" OR "streptococcal pneumoniae" OR "streptococcus p" OR "streptococcal p") AND Intervention=(vaccine OR vaccines OR vaccination OR immunisation OR immunization OR immunise OR immunisation OR inoculate) \| Child \| First posted from 06/01/2019 to 01/01/2024 | |
| Other terms=(pneumococcal OR pneumococcus OR "streptococcus pneumoniae" OR "streptococcal pneumoniae" OR "streptococcus p" OR "streptococcal p") AND (conjugate OR valent) \| Child | |
| Title=(pneumococcal OR pneumococcus OR "streptococcus pneumoniae" OR "streptococcal pneumoniae" OR "streptococcus p" OR "streptococcal p") AND (conjugate OR valent) \| Child | |
| Condition=(pneumococcal OR pneumococcus OR "streptococcus pneumoniae" OR "streptococcal pneumoniae" OR "streptococcus p" OR "streptococcal p") AND Intervention=(conjugate OR valent) \| Child | |
| Other terms=pcv7 or pcv 7 or pncrm or pnccrm or 7vpnc or 7vcrm or pcv9 or pcv 9 or pcv10 or pcv 10 or phidcv or phid cv or pcv13 or pcv 13 or 13vcrm \| Child | |
| Title=pcv7 or pcv 7 or pncrm or pnccrm or 7vpnc or 7vcrm or pcv9 or pcv 9 or pcv10 or pcv 10 or phidcv or phid cv or pcv13 or pcv 13 or 13vcrm \| Child | |
| Intervention=pcv7 or pcv 7 or pncrm or pnccrm or 7vpnc or 7vcrm or pcv9 or pcv 9 or pcv10 or pcv 10 or phidcv or phid cv or pcv13 or pcv 13 or 13vcrm \| Child | |
| Other terms=pneumovax or pneumopur or streptopur or streptorix or prevnar or prevenar or synflorix or gsk 1024850a or gsk1024850a \| Child | |
| Title=pneumovax or pneumopur or streptopur or streptorix or prevnar or prevenar or synflorix or gsk 1024850a or gsk1024850a \| Child | |
| Intervention=pneumovax or pneumopur or streptopur or streptorix or prevnar or prevenar or synflorix or gsk 1024850a or \| Child | |
| **WHO ICTRP** | |
| pneumococcal vaccine OR pneumococcus vaccine OR pneumococcal conjugate OR pneumococcus conjugate OR pcv OR pneumovax OR pneumopur OR streptopur OR streptorix OR prevnar OR prevenar OR synflorix OR gsk 1024850a OR gsk1024850a - TRIALS IN CHILDREN | |
| **Cochrane (CDSR - limited to Publication date: 01/06/2019-27/07/2022, CENTRAL - limited to Added to database date: 01/06/2019-27/07/2022)** | |
| **ID** | **Search** |
| #1 | MeSH descriptor: [Streptococcus pneumoniae] explode all trees |
| #2 | (pneumococc* or "s pneumoniae" or "streptococcus pneumoniae" or "streptococcus p" or "streptococcal pneumoniae" or "streptococcal p") |
| #3 | #1 or #2 |
| #4 | MeSH descriptor: [Vaccines] this term only |
| #5 | MeSH descriptor: [Bacterial Vaccines] this term only |
| #6 | ((vaccin* or immunization* or immunize* or immunisation* or immunise* or inoculat* or conjugate or valent)):ti,ab,kw |
| #7 | #4 or #5 or #6 |
| #8 | #3 and #7 |
| #9 | MeSH descriptor: [Pneumococcal Vaccines] explode all trees |
| #10 | ((pcv7 or "pcv 7" or pncrm* or pnccrm* or 7vpnc or 7vcrm)):ti,ab,kw |
| #11 | ((pcv9 or "pcv 9")):ti,ab,kw |
| #12 | (pcv10 or" pcv 10" or phidcv or "phid cv"):ti,ab,kw |
| #13 | (pcv13 or "pcv 13" or 13vcrm):ti,ab,kw |
| #14 | pneumovax or pneumopur or streptopur or streptorix or prevnar or prevenar or synflorix or gsk 1024850a or gsk1024850a |
| #15 | #8 or #9 or #10 or #11 or #13 or #14 |
| #16 | MeSH descriptor: [Child] explode all trees |
| #17 | MeSH descriptor: [Infant] this term only |
| #18 | (child* or infan* or baby or babies or toddler* or preschool* or pre-school* or pediatric* or paediatric*):ti,ab,kw |
| #19 | #16 or #17 or #18 |
| #20 | #15 and #19 |

Supplementary Table 2. Summary of eligible studies excluded from network meta-analysis due to either no closed loop or data unavailable.

|  | Author & Year* | NCT | Comparison |
| --- | --- | --- | --- |
| Studies with no closed loop | GlaxoSmithKline^4^ | NCT00169481 | pcv7 vs pcv11 |
|  | Dagan et al. 1996^5^ | Not found | pcv7 vs ppv23 |
|  | Greenberg et al. 2018^6^ | NCT01215188 | pcv13 vs pcv15 |
|  | Rupp et al. 2019^7^ | NCT0251373  NCT02037984 | pcv13 vs pcv15 |
|  | Thisyakorn et al. 2014^8^ | NCT00594347 | pcv7 vs ppv23 |
|  | Martinez et al. 2018^9^ | RPCE00000173 | Cuban pcv7 vs pcv10 |
|  | Platt et al. 2020^10^ | NCT02987972 | pcv15 vs pcv13 |
|  | Bili et al. 2021^11^;  Bili et al. 2023^12^ | NCT03620162 | pcv15 vs pcv13 |
|  | Shin et al. 2020^13^ | NCTR20170109002 | pcv12 vs pcv13 |
|  | Chen et al. 2016^14^;  Zhao et al. 2022^15^ | NCT02736240 | pcv7 vs Chinese pcv13 |
|  | Senders et al. 2021^16^; Senders et al. 2020^17^ | NCT03512288 | pcv13 vs pcv20 |
|  | Banniettis et al. 2021^18^;  Banniettis et la. 2022^19^ | NCT03885934 | pcv15 vs pcv13 |
|  | Lupinacci et al. 2022^20^ ;  Lupinacci et al. 2023^21^ | NCT03893448 | pcv15 vs pcv13 |
|  |  |  |  |
|  |  |  |  |
| Studies with data unavailable | Martinon-Torres et al. 2012^22^ | NCT00474539 | pcv7 vs pcv13 |
|  | Vanderkooi et al. 2012^23^ | NCT00475033 | pcv7 vs pcv13 |
|  | De Los Santos et al. 2017^24^ | NCT01641133 | pcv10 vs pcv13 |
|  | Diez-Domingo et al. 2013^25^ | NCT00368966 | pcv7 vs pcv13 |
|  | Clarke et al. 2020^26^* | NCT02308540 | pcv10-SII vs pcv13 |
|  | Clarke et al. 2021^27^* | NCT03197376 | pcv10 vs pcv10-SII |

* Individual participant data unavailable

PCV – Pneumococcal conjugate vaccine; PPSV - Pneumococcal polysaccharide vaccine; PCV10-SII – 10-valent Pneumococcal conjugate vaccine by Serum Institute of India

Supplementary Table 3. Summary of the statistical heterogeneity and incoherence for immunogenicity analyses shown in Figure 2

| Serotype | Time point | No. of study | No. and proportion of study providing direct evidence | | No. of participants providing direct evidence | No. of participants providing indirect evidence | Tau (τ) | I^2^ (%) | p-value (Q of heterogeneity and inconsistency; df) | p-value (Q of heterogeneity; df) | p-value (Q of inconsistency; df) |
| --- | --- | --- | --- | --- | --- | --- | --- | --- | --- | --- | --- |
| **4** | post prime | 28 | 9 | 47.9% | 2,356 | 9,258 | 0.089 | 86.8  (82.0-90.4) | 0.000 (197.440;26) | 0.000 (196.733;25) | 0.400 (0.707;1) |
| **6B** | post prime | 28 | 9 | 59.7% | 2,356 | 9,194 | 0.109 | 82.2  (75.0-87.3) | 0.000 (145.913;26) | 0.000 (108.043;25) | 0.000 (37.870;1) |
| **9V** | post prime | 28 | 9 | 48.0% | 2,360 | 9,241 | 0.078 | 83.8  (77.5-88.4) | 0.000 (160.708;26) | 0.000 (158.678;25) | 0.154 (2.030;1) |
| **14** | post prime | 28 | 9 | 48.6% | 2,358 | 9,186 | 0.069 | 71.1  (57.4-80.4) | 0.000 (89.983;26) | 0.000 (83.435;25) | 0.010 (6.548;1) |
| **18C** | post prime | 28 | 9 | 50.9% | 2,358 | 9,258 | 0.147 | 93.7  (91.9-95.1) | 0.000 (413.924;26) | 0.000 (405.782;25) | 0.004 (8.142;1) |
| **19F** | post prime | 28 | 9 | 51.8% | 2,358 | 9,235 | 0.199 | 96.2  (95.3-96.9) | 0.000 (679.122;26) | 0.000 (675.147;25) | 0.046 (3.975;1) |
| **23F** | post prime | 28 | 9 | 55.1% | 2,358 | 9,211 | 0.095 | 80.6  (72.5-86.3) | 0.000 (133.979;26) | 0.000 (131.123;25) | 0.091 (2.856;1) |
| **1** | post prime | 9 | 9 | 100% | 2,357 |  | 0.177 | 93.8  (90.4-96.1) | 0.000 (130.029;8) | 0.000 (130.029;8) |  |
| **5** | post prime | 9 | 9 | 100% | 2,356 |  | 0.193 | 95.1  (92.5-96.7) | 0.000 (161.837;8) | 0.000 (161.837;8) |  |
| **7F** | post prime | 9 | 9 | 100% | 2,358 |  | 0.056 | 63.6  (25.3-82.3) | 0.005 (21.980;8) | 0.005 (21.980;8) |  |
| **3** | post prime | 9 | 9 | 100% | 2,354 |  | 0.495 | 99.2  (99.0-99.4) | 0.000 (1006.362;8) | 0.000 (1006.362;8) |  |
| **6A** | post prime | 9 | 9 | 100% | 2,354 |  | 0.514 | 99.0  (98.8-99.2) | 0.000 (825.778;8) | 0.000 (825.778;8) |  |
| **19A** | post prime | 9 | 9 | 100% | 2,356 |  | 0.381 | 98.1  (97.5-98.6) | 0.000 (428.259;8) | 0.000 (428.259;8) |  |
| **4** | prior booster | 17 | 7 | 68.9% | 1,816 | 4,174 | 0.098 | 83.7  (74.8-89.4) | 0.000 (92.071;15) | 0.000 (90.267;14) | 0.179 (1.805;1) |
| **6B** | prior booster | 18 | 8 | 78.7% | 2,220 | 4,140 | 0.161 | 90.8  (86.8-93.6) | 0.000 (173.775;16) | 0.000 (171.746;15) | 0.154 (2.029;1) |
| **9V** | prior booster | 18 | 8 | 72.7% | 2,218 | 4,175 | 0.108 | 87.9  (82.2-91.8) | 0.000 (132.395;16) | 0.000 (131.952;15) | 0.506 (0.443;1) |
| **14** | prior booster | 18 | 8 | 69.3% | 2,224 | 4,178 | 0.082 | 70.1  (50.9-81.8) | 0.000 (53.432;16) | 0.000 (53.136;15) | 0.586 (0.296;1) |
| **18C** | prior booster | 17 | 7 | 65.6% | 1,806 | 4,177 | 0.089 | 82.0  (71.8-88.5) | 0.000 (83.259;15) | 0.000 (68.689;14) | 0.000 (14.571;1) |
| **19F** | prior booster | 18 | 8 | 82.0% | 2,215 | 4,151 | 0.12 | 83.2  (74.3-89.0) | 0.000 (95.125;16) | 0.000 (69.247;15) | 0.000 (25.878;1) |
| **23F** | prior booster | 18 | 8 | 76.9% | 2,216 | 4,170 | 0.115 | 82.8  (73.5-88.8) | 0.000 (92.759;16) | 0.000 (92.450;15) | 0.578 (0.309;1) |
| **1** | prior booster | 8 | 8 | 100.0% | 2,228 |  | 0.127 | 90.1  (83.0-94.3) | 0.000 (71.032;7) | 0.000 (71.032) |  |
| **5** | prior booster | 8 | 8 | 100.0% | 2,221 |  | 0.077 | 77.2  (54.7-88.5) | 0.000 (30.664;7) | 0.000 (30.664) |  |
| **7F** | prior booster | 8 | 8 | 100.0% | 2,220 |  | 0.083 | 79.4  (59.8-89.4) | 0.000 (33.968;7) | 0.000 (33.968) |  |
| **3** | prior booster | 7 | 7 | 100.0% | 1,813 |  | 0.236 | 95.0  (91.8-96.9) | 0.000 (119.248;6) | 0.000 (119.248) |  |
| **6A** | prior booster | 8 | 8 | 100.0% | 2,213 |  | 0.169 | 92.2  (87.0-95.3) | 0.000 (89.441;7) | 0.000 (89.441) |  |
| **19A** | prior booster | 8 | 8 | 100.0% | 2,219 |  | 0.132 | 84.8  (71.9-91.8) | 0.000 (46.063;7) | 0.000 (46.063) |  |
| **4** | post booster | 25 | 6 | 53.1% | 1,706 | 8,457 | 0.063 | 72.9  (59.3-81.9) | 0.000 (84.813;23) | 0.000 (75.091;22) | 0.002 (9.722;1) |
| **6B** | post booster | 26 | 7 | 60.0% | 2,102 | 8,434 | 0.11 | 86.5  (81.3-90.3) | 0.000 (178.002;24) | 0.000 (176.999;23) | 0.317 (1.003;1) |
| **9V** | post booster | 26 | 7 | 57.9% | 2,101 | 8,457 | 0.076 | 82.5  (75.1-87.7) | 0.000 (136.818;24) | 0.000 (135.005;23) | 0.178 (1.812;1) |
| **14** | post booster | 26 | 7 | 51.0% | 2,100 | 8,446 | 0.063 | 71.7  (57.8-81.1) | 0.000 (84.914;24) | 0.000 (84.381;23) | 0.466 (0.532;1) |
| **18C** | post booster | 25 | 6 | 54.0% | 1,705 | 8,453 | 0.074 | 79.6  (70.3-86.0) | 0.000 (112.643;23) | 0.000 (112.118;22) | 0.469 (0.525;1) |
| **19F** | post booster | 26 | 7 | 58.1% | 2,102 | 8,443 | 0.111 | 87.4  (82.6-90.8) | 0.000 (190.038;24) | 0.000 (186.332;23) | 0.054 (3.706;1) |
| **23F** | post booster | 26 | 7 | 64.3% | 2,100 | 8,437 | 0.08 | 76.4  (65.5-83.9) | 0.000 (101.839;24) | 0.000 (101.839;23) | 1.000 (0.000;1) |
| **1** | post booster | 7 | 7 | 100.0% | 2,100 |  | 0.048 | 54.6  (0.0-80.6) | 0.040 (13.216;6) | 0.040 (13.216) |  |
| **5** | post booster | 7 | 7 | 100.0% | 2,101 |  | 0.092 | 83.4  (67.2-91.6) | 0.000 (36.074;6) | 0.000 (36.074) |  |
| **7F** | post booster | 7 | 7 | 100.0% | 2,100 |  | 0.055 | 70.0  (34.2-86.3) | 0.003 (19.981;6) | 0.003 (19.981) |  |
| **3** | post booster | 6 | 6 | 100.0% | 1,701 |  | 0.299 | 96.9  (95.2-98.0) | 0.000 (162.413;5) | 0.000 (162.413) |  |
| **6A** | post booster | 7 | 7 | 100.0% | 2,099 |  | 0.232 | 94.2  (90.4-96.5) | 0.000 (103.568;6) | 0.000 (103.568) |  |
| **19A** | post booster | 7 | 7 | 100.0% | 2,100 |  | 0.147 | 86.4  (74.0-92.8) | 0.000 (43.957;6) | 0.000 (43.957) |  |

df: degree of freedom; tau: square-root of between-study variance; I^2^: heterogeneity statistic; Q of heterogeneity and inconsistency: overall heterogeneity/inconsistency statistic. Q of heterogeneity: overall heterogeneity statistic. Q of inconsistency: overall inconsistency statistic.

Supplementary Table 4. Summary on assessment of statistical heterogeneity and incoherence for seroefficacy analysis shown in Figure 3

| Serotype | No. of study | No. and proportion of study providing direct evidence | | No. of participants providing direct evidence | No. of participants providing indirect evidence | tau | I^2^ (%) | p-value (Q of heterogeneity and inconsistency; df) | | p-value (Q of heterogeneity; df) | | p-value (Q of inconsistency; df) | |  |
| --- | --- | --- | --- | --- | --- | --- | --- | --- | --- | --- | --- | --- | --- | --- |
| **4** | 15 | 6 | 89.0% | 1,577 | 3,531 | 0.383 | 23.7  (0-59.5) | | 0.198 (17.036;13) | | 0.178 (16.286;12) | | 0.386 (0.750;1) | |
| **6B** | 15 | 6 | 91.6% | 1,573 | 3,466 | 0.249 | 74.4  (56.7-84.9) | | 0.000 (50.756;13) | | 0.000 (48.136;12) | | 0.105 (2.621;1) | |
| **9V** | 15 | 6 | 90.7% | 1,574 | 3,518 | 0.309 | 43.0  (0-69.6) | | 0.044 (22.801;13) | | 0.049 (21.065;12) | | 0.188 (1.736;1) | |
| **14** | 15 | 6 | 81.3% | 1,578 | 3,524 | 0.361 | 69.6  (47.4-82.5) | | 0.000 (42.834;13) | | 0.000 (40.642;12) | | 0.139 (2.192;1) | |
| **18C** | 15 | 6 | 95.1% | 1,568 | 3,537 | 0.474 | 49.9  (7.4-72.9) | | 0.017 (25.961;13) | | 0.017 (24.657;12) | | 0.254 (1.304;1) | |
| **19F** | 15 | 6 | 80.2% | 1,569 | 3,497 | 0.327 | 49.1  (5.7-72.5) | | 0.020 (25.541;13) | | 0.104 (18.415;12) | | 0.008 (7.126;1) | |
| **23F** | 15 | 6 | 93.6% | 1,570 | 3,498 | 0.557 | 83.8  (74.3-89.9) | | 0.000 (80.480;13) | | 0.000 (78.982;12) | | 0.221 (1.498;1) | |
| **1** | 6 | 6 | 100% | 1,581 |  | 0.403 | 42.7  (0-77.3) | | 0.121 (8.724;5) | | 0.121 (8.724;5) | |  | |
| **5** | 6 | 6 | 100% | 1,573 |  | 0.975 | 82.2  (62.1-91.6) | | 0.000 (28.031;5) | | 0.000 (28.031;5) | |  | |
| **7F** | 6 | 6 | 100% | 1,575 |  | 0.000 | 0  (0-74.6) | | 0.537 (4.084;5) | | 0.537 (4.084;5) | |  | |
|  |  |  |  |  |  |  |  | |  | |  | |  | |
|  |  |  |  |  |  |  |  | |  | |  | |  | |
|  |  |  |  |  |  |  |  | |  | |  | |  | |

df: degree of freedom; tau: square-root of between-study variance; I^2^: heterogeneity statistic; Q of heterogeneity and inconsistency: overall heterogeneity/inconsistency statistic. Q of heterogeneity: overall heterogeneity statistic. Q of inconsistency: overall inconsistency statistic.

Supplementary Figure 1. Network of studies included for a) all eligible cohorts, b) immunogenicity analysis cohorts and c) seroefficacy analysis cohorts

a）
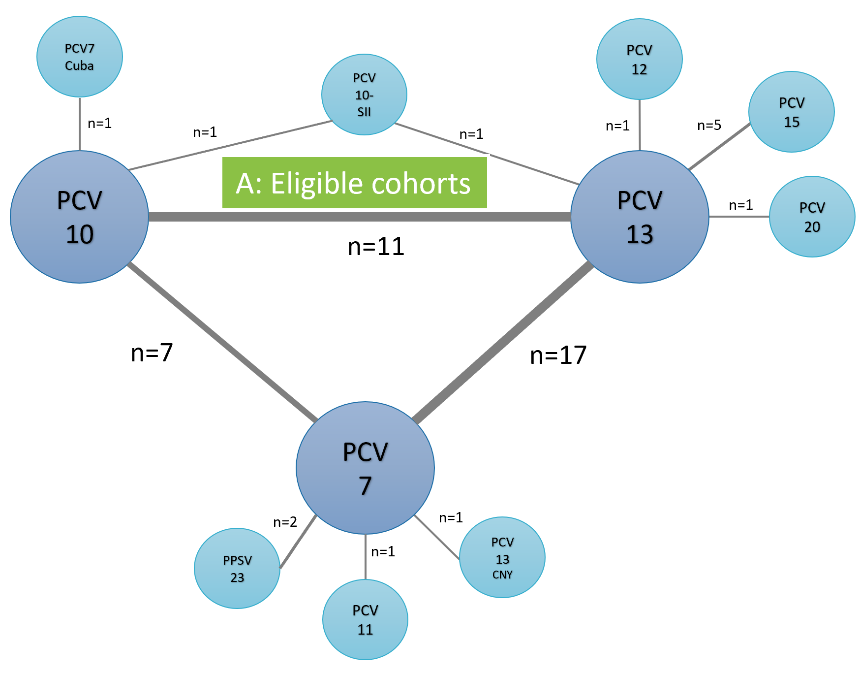


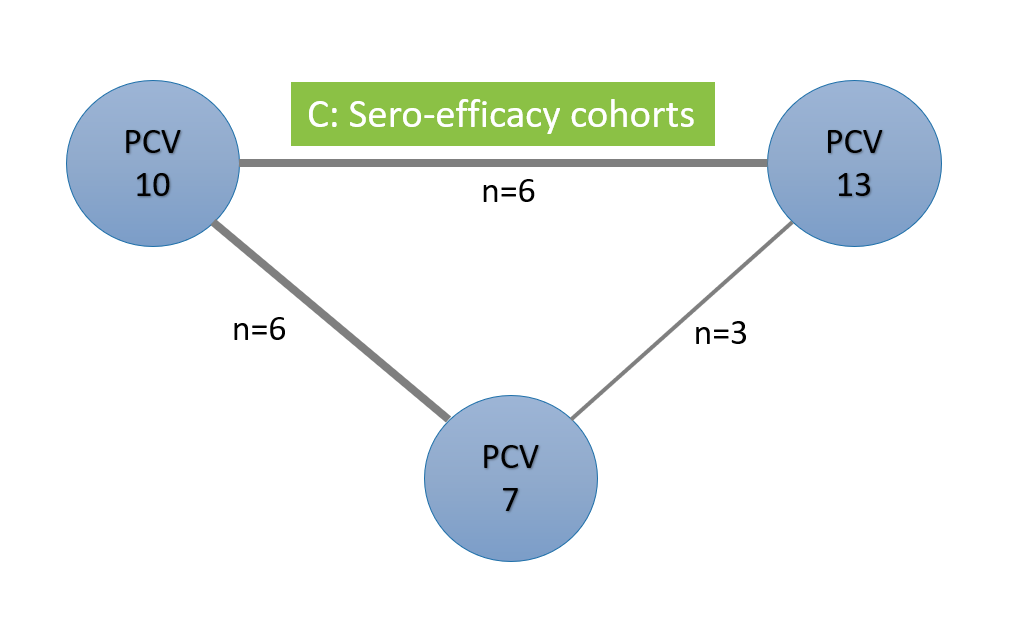
b)
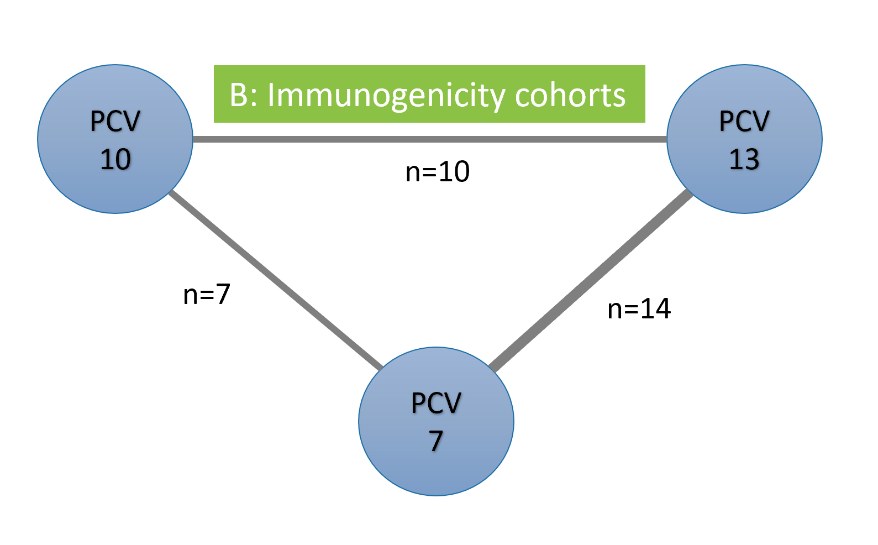
 c)

PCV – Pneumococcal conjugate vaccine; PPSV - Pneumococcal polysaccharide vaccine; PCV10-SII – 10-valent Pneumococcal conjugate vaccine by Serum Institute of India; PCV7 Cuba – a Cuban PCV7; PCV13 CNY – a Chinese PCV13

Supplementary Figure 2. Assessment of risk of bias for included studies


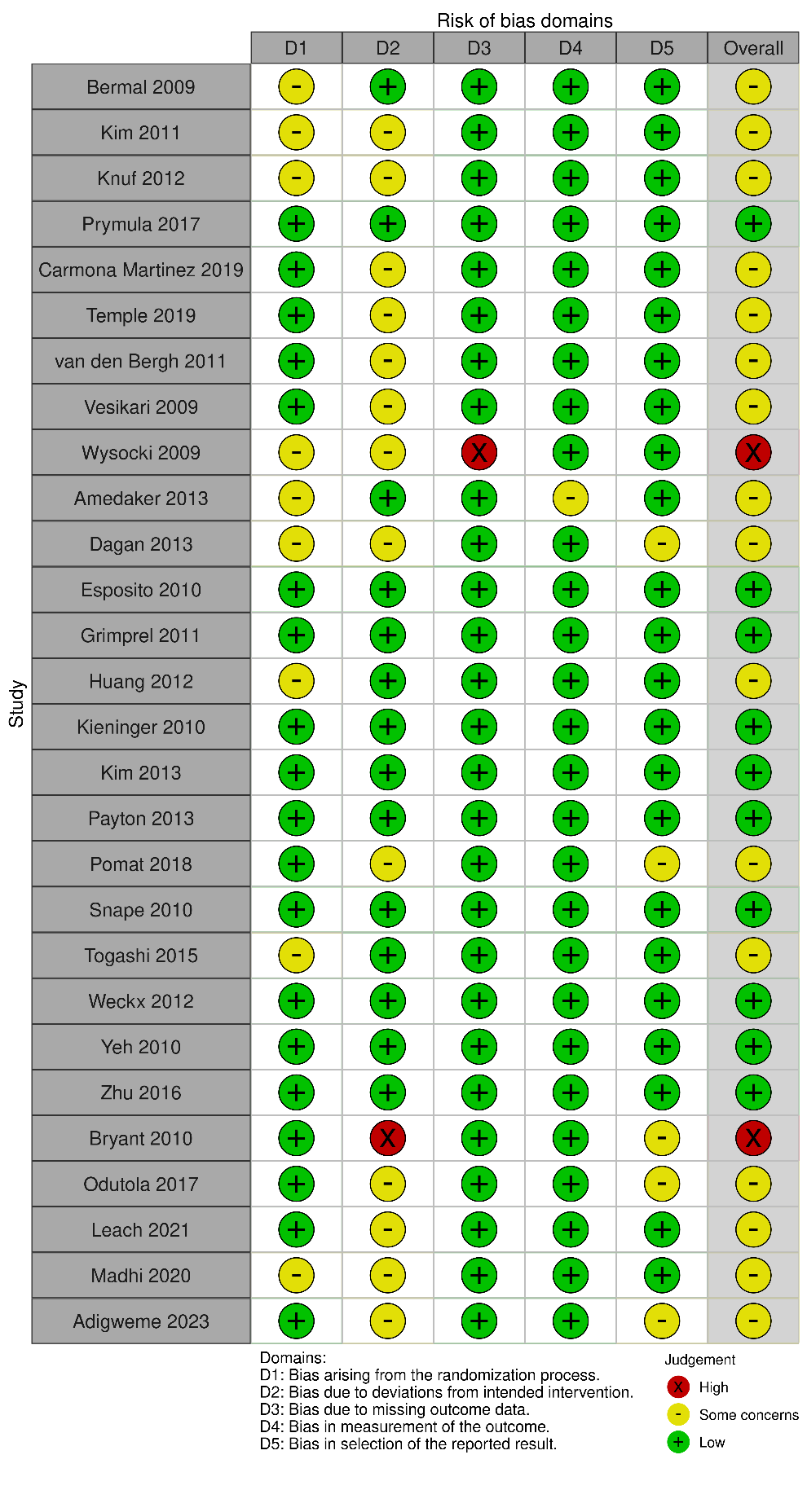


Supplementary Figure 3. Direct and indirect evidence on geometric mean ratios comparing PCV13 vs PCV10 for serotypes in PCV7 at a) 28 days post-primary vaccination series, b) pre-booster, and c) 28 days post-booster

a)
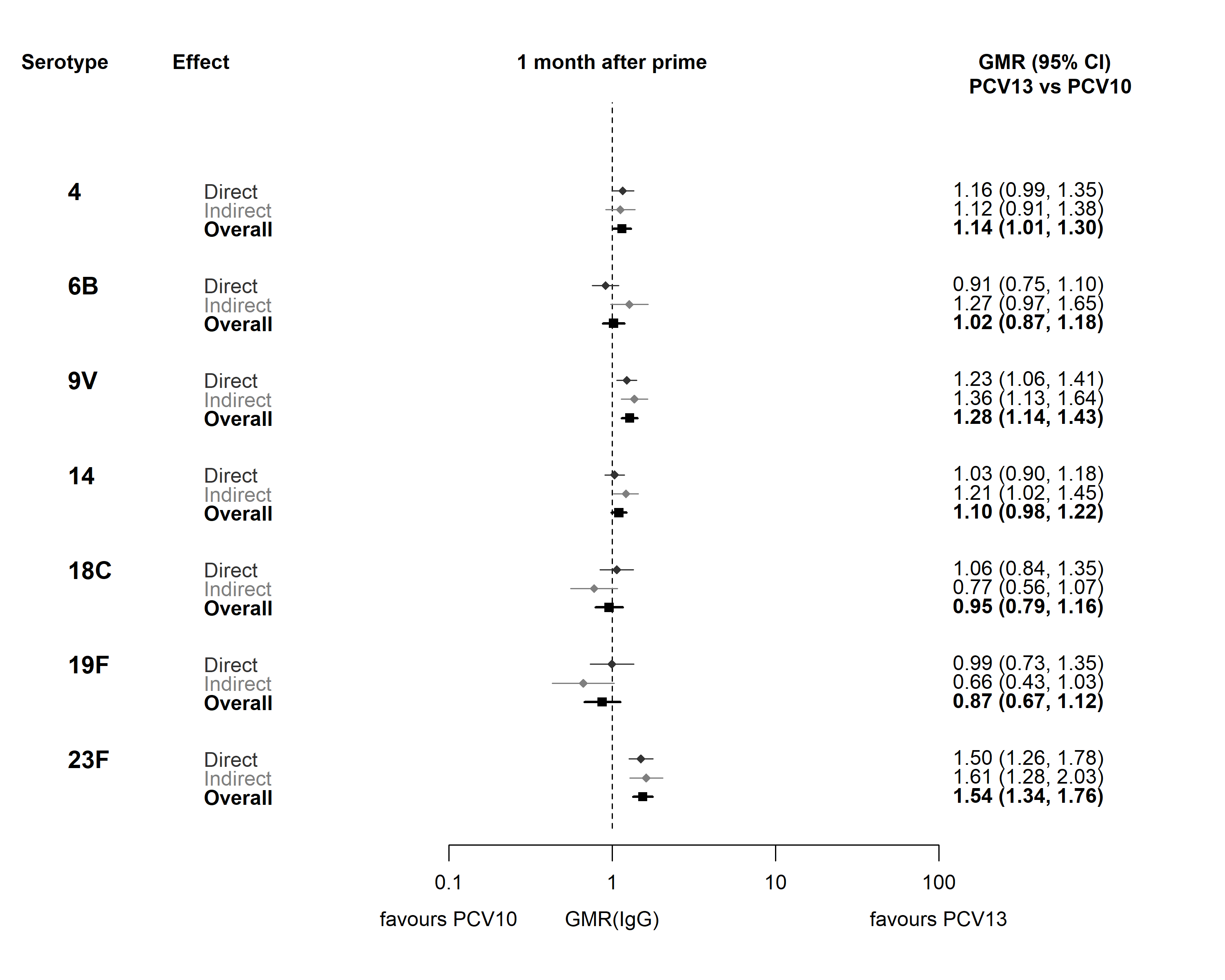


b)
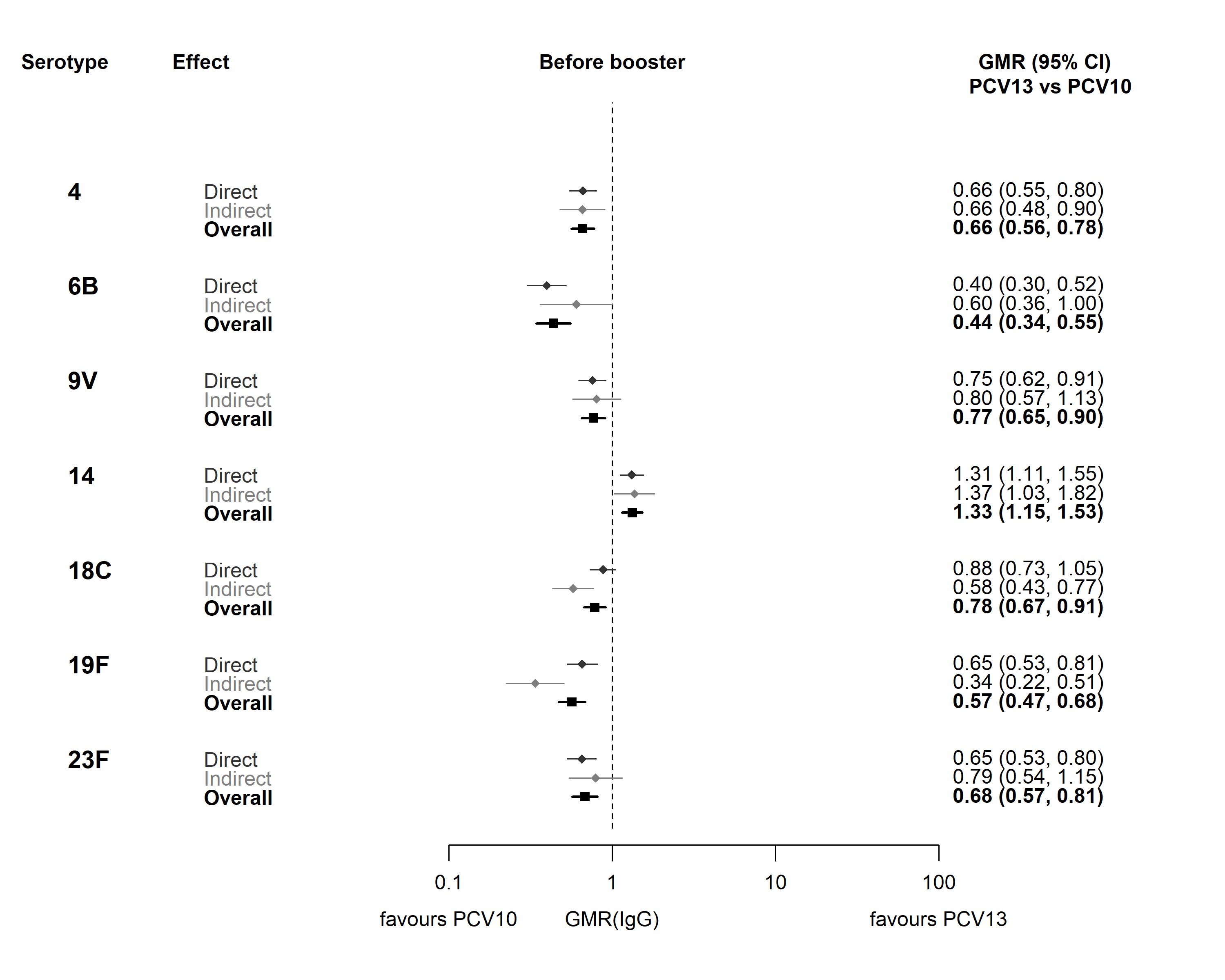


c)
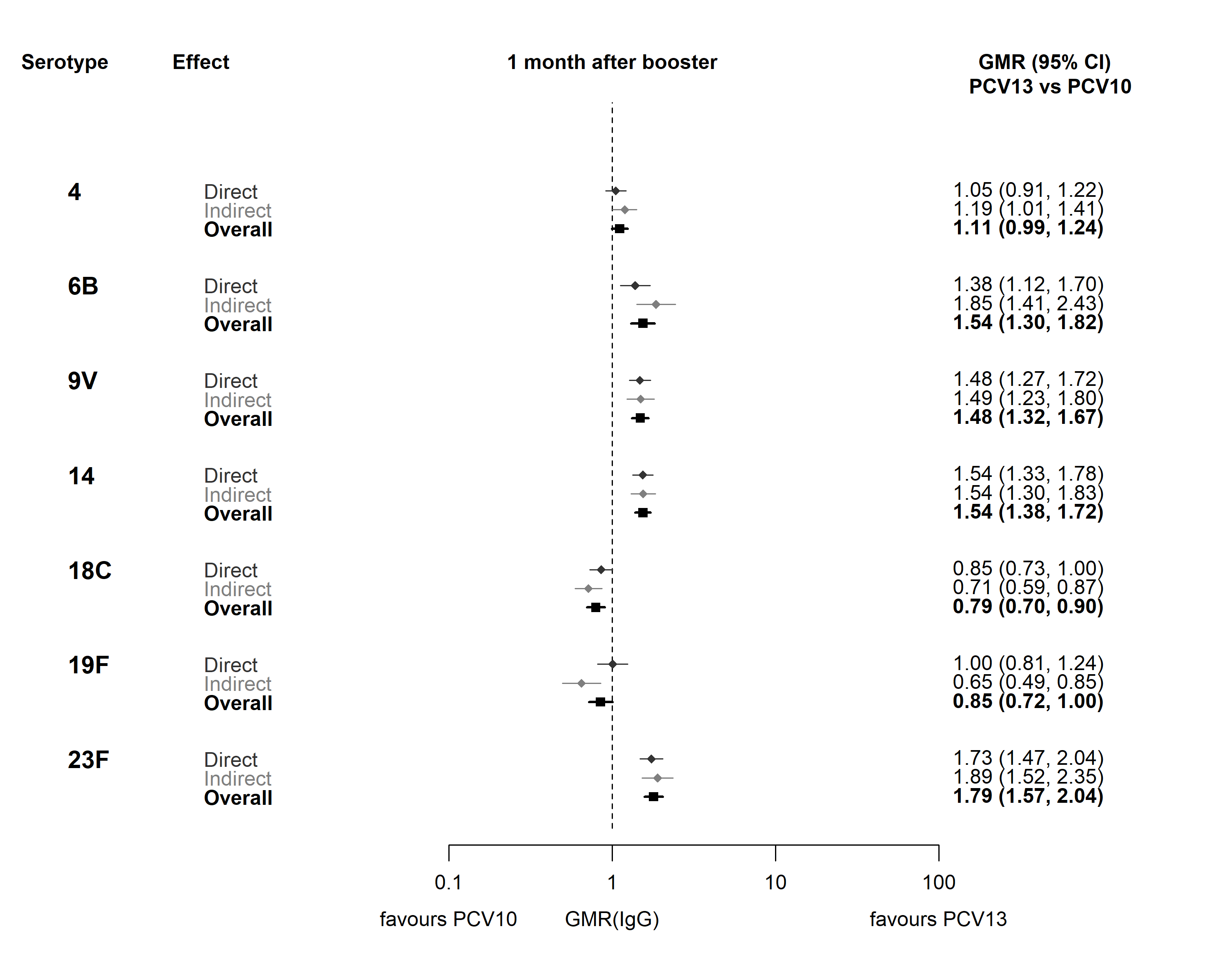


GMR: Geometric mean ratio; PCV: Pneumococcal conjugate vaccine. Each line in the figure shows the output from a network meta-analyses. Dark grey diamonds and lines show the point estimates and confidence intervals for geometric mean ratios from studies directly comparing PCV13 vs PCV10. Light grey diamonds and lines show the point estimates and confidence intervals for geometric mean ratios from studies comparing PCV13 vs PCV10 through PCV7. Black boxes and lines show the point estimates and confidence intervals incorporating both direct and indirect evidence.

Supplementary Figure 4. Trial level geometric mean ratios for serotype 4 post-primary vaccination series.


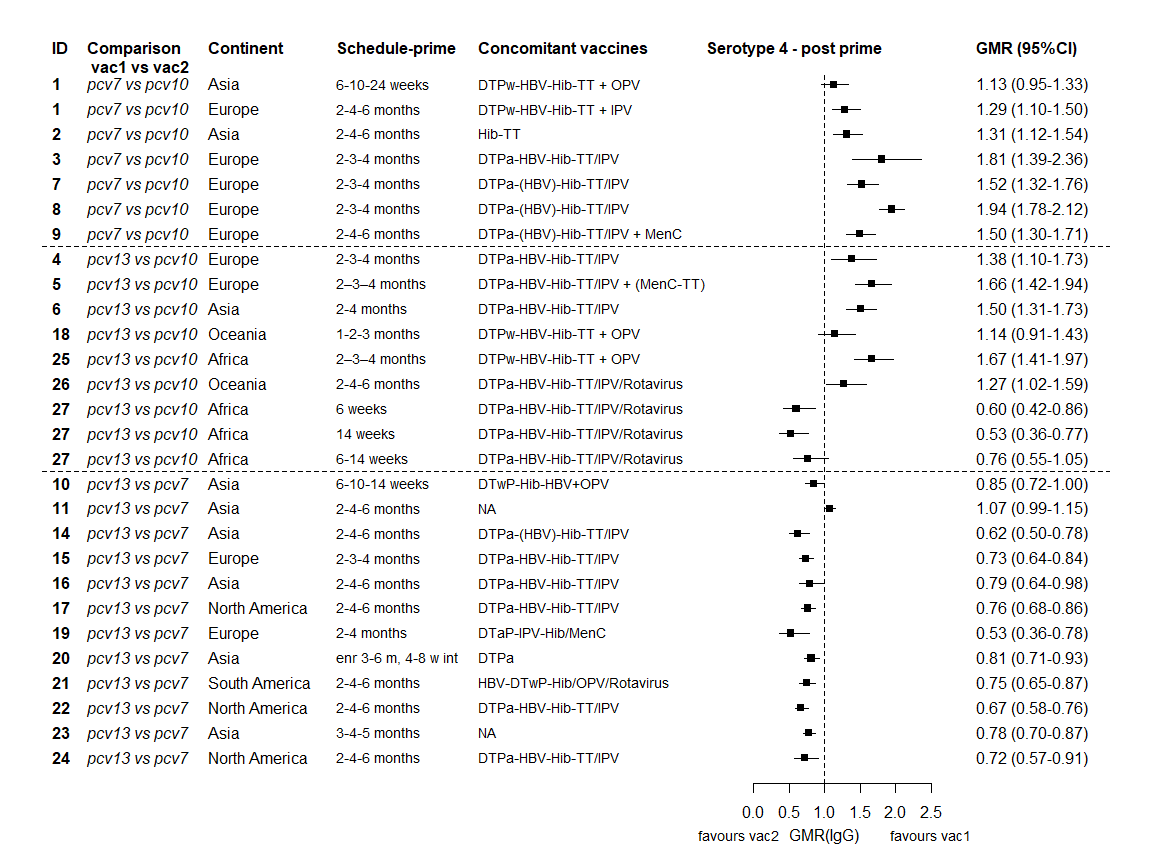


GMR: Geometric mean ratio; pcv: Pneumococcal conjugate vaccine; DTaP – diphtheria and tetanus toxoids, and acellular pertussis vaccine; DTwP – diphtheria and tetanus toxoids, and whole-cell pertussis vaccine; Hib-TT – Haemophilus influenzae type b vaccine (tetanus toxoid conjugate); HB – Hepatitis B vaccine; IPV – Inactivated polio vaccine; OPV – Oral polio vaccine; MenC – Meningococcal C vaccine; TT – tetanus toxoid conjugate; NA: not applicable; enr 3-6 m, 4-8 w int: enrolment at 3-6 months of age, and at 4-8 weeks interval of a 3 doses primary vaccines in total.

Each solid line in the figure shows the GMR from each trial. Black boxes and lines show the point estimates and confidence intervals for geometric mean ratios comparing vac1 vs vac2. Concomitant vaccines are vaccines co-administered with PCV primary vaccine series. Information on co-administered vaccine is not always available (e.g. study ID 11 and 23). Concomitant vaccines in the bracket are those administered in some but not all of the study sites.

Supplementary Figure 5. Trial level geometric mean ratios for serotype 6B post-primary vaccination series.


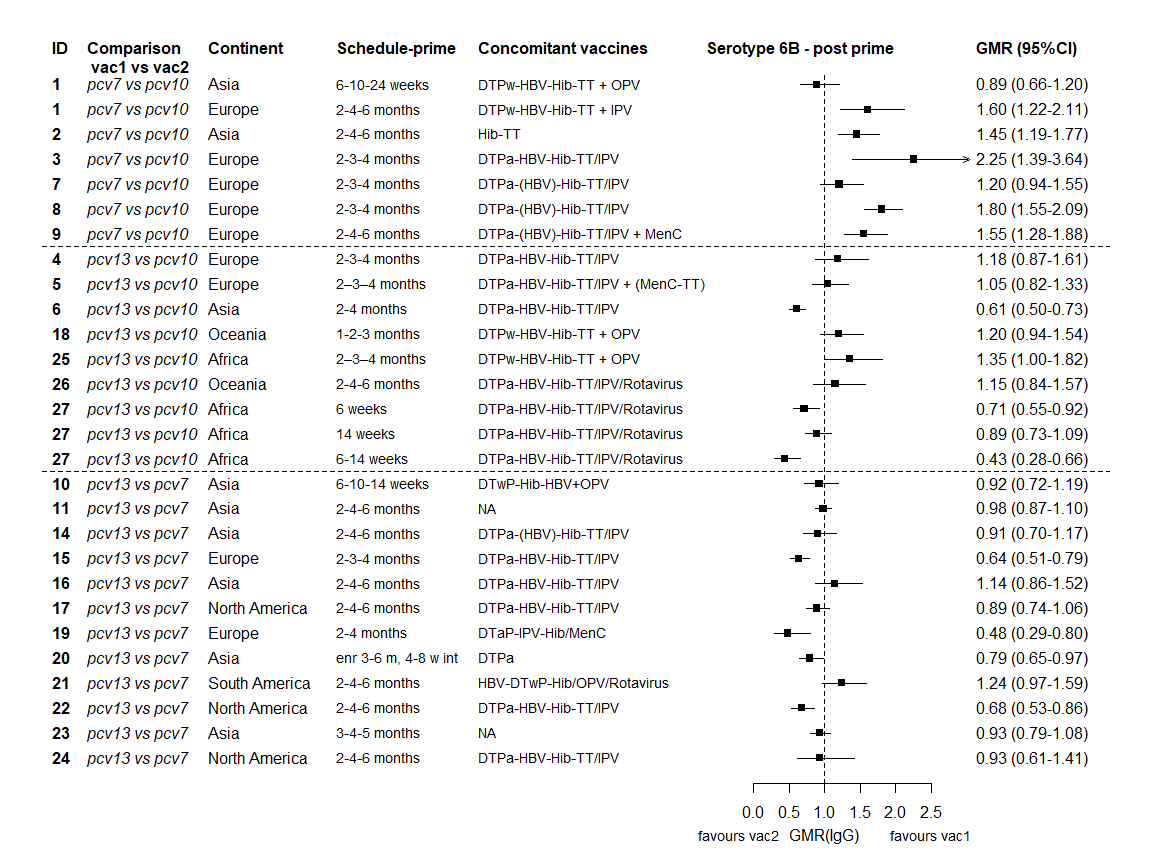


GMR: Geometric mean ratio; pcv: Pneumococcal conjugate vaccine; DTaP – diphtheria and tetanus toxoids, and acellular pertussis vaccine; DTwP – diphtheria and tetanus toxoids, and whole-cell pertussis vaccine; Hib-TT – Haemophilus influenzae type b vaccine (tetanus toxoid conjugate); HB – Hepatitis B vaccine; IPV – Inactivated polio vaccine; OPV – Oral polio vaccine; MenC – Meningococcal C vaccine; TT – tetanus toxoid conjugate; NA: not applicable; enr 3-6 m, 4-8 w int: enrolment at 3-6 months of age, and at 4-8 weeks interval of a 3 doses primary vaccines in total.

Each solid line in the figure shows the GMR from each trial. Black boxes and lines show the point estimates and confidence intervals for geometric mean ratios comparing vac1 vs vac2. Concomitant vaccines are vaccines co-administered with PCV primary vaccine series. Information on co-administered vaccine is not always available (e.g. study ID 11 and 23). Concomitant vaccines in the bracket are those administered in some but not all of the study sites.

Supplementary Figure 6. Trial level geometric mean ratios for serotype 9V post-primary vaccination series.


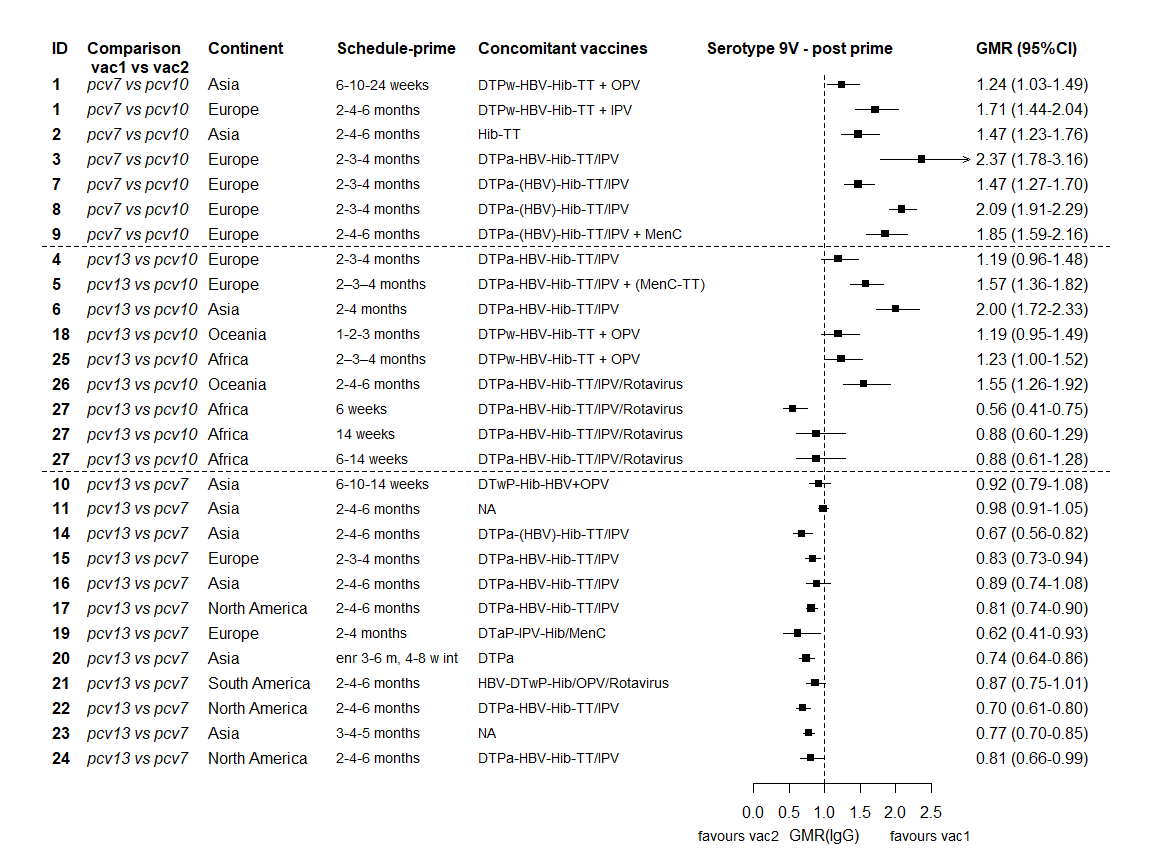


GMR: Geometric mean ratio; pcv: Pneumococcal conjugate vaccine; DTaP – diphtheria and tetanus toxoids, and acellular pertussis vaccine; DTwP – diphtheria and tetanus toxoids, and whole-cell pertussis vaccine; Hib-TT – Haemophilus influenzae type b vaccine (tetanus toxoid conjugate); HB – Hepatitis B vaccine; IPV – Inactivated polio vaccine; OPV – Oral polio vaccine; MenC – Meningococcal C vaccine; TT – tetanus toxoid conjugate; NA: not applicable; enr 3-6 m, 4-8 w int: enrolment at 3-6 months of age, and at 4-8 weeks interval of a 3 doses primary vaccines in total.

Each solid line in the figure shows the GMR from each trial. Black boxes and lines show the point estimates and confidence intervals for geometric mean ratios comparing vac1 vs vac2. Concomitant vaccines are vaccines co-administered with PCV primary vaccine series. Information on co-administered vaccine is not always available (e.g. study ID 11 and 23). Concomitant vaccines in the bracket are those administered in some but not all of the study sites.

Supplementary Figure 7. Trial level geometric mean ratios for serotype 14 post-primary vaccination series.


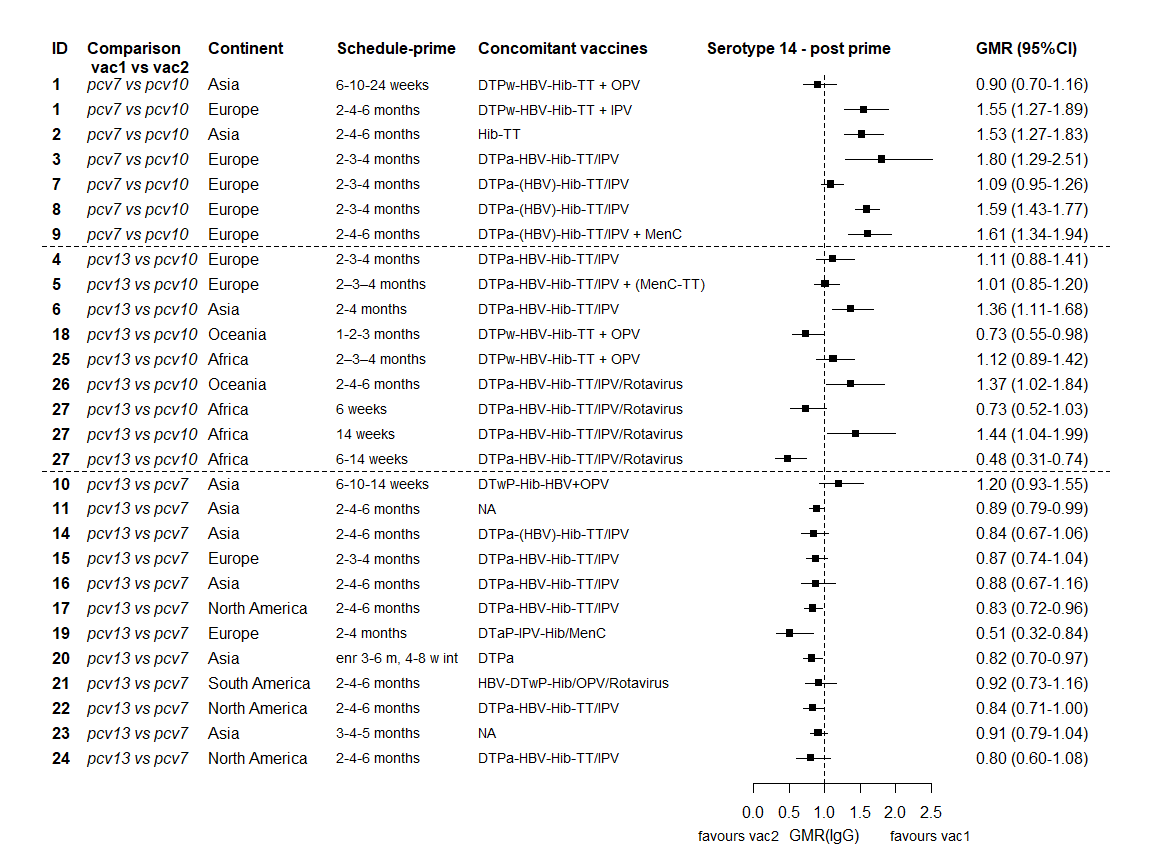


GMR: Geometric mean ratio; pcv: Pneumococcal conjugate vaccine; DTaP – diphtheria and tetanus toxoids, and acellular pertussis vaccine; DTwP – diphtheria and tetanus toxoids, and whole-cell pertussis vaccine; Hib-TT – Haemophilus influenzae type b vaccine (tetanus toxoid conjugate); HB – Hepatitis B vaccine; IPV – Inactivated polio vaccine; OPV – Oral polio vaccine; MenC – Meningococcal C vaccine; TT – tetanus toxoid conjugate; NA: not applicable; enr 3-6 m, 4-8 w int: enrolment at 3-6 months of age, and at 4-8 weeks interval of a 3 doses primary vaccines in total.

Each solid line in the figure shows the GMR from each trial. Black boxes and lines show the point estimates and confidence intervals for geometric mean ratios comparing vac1 vs vac2. Concomitant vaccines are vaccines co-administered with PCV primary vaccine series. Information on co-administered vaccine is not always available (e.g. study ID 11 and 23). Concomitant vaccines in the bracket are those administered in some but not all of the study sites.

Supplementary Figure 8. Trial level geometric mean ratios for serotype 18C post-primary vaccination series.


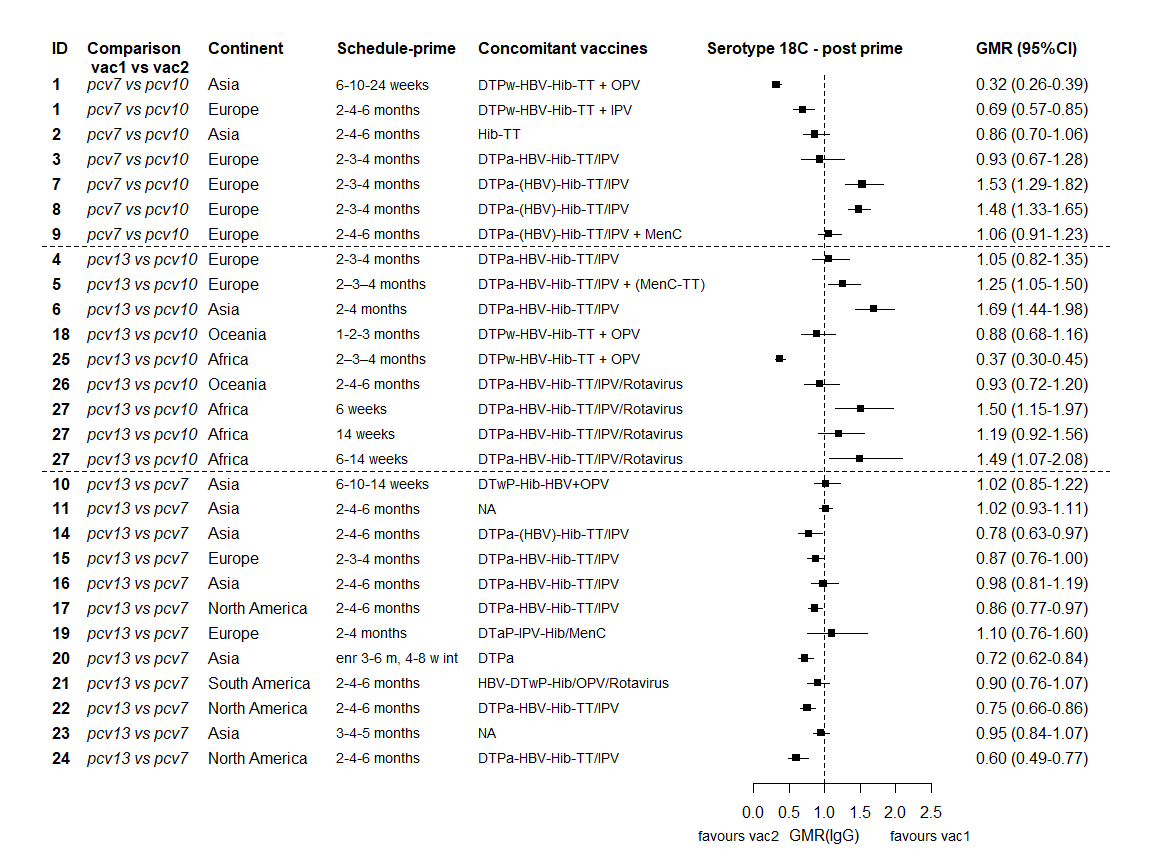


GMR: Geometric mean ratio; pcv: Pneumococcal conjugate vaccine; DTaP – diphtheria and tetanus toxoids, and acellular pertussis vaccine; DTwP – diphtheria and tetanus toxoids, and whole-cell pertussis vaccine; Hib-TT – Haemophilus influenzae type b vaccine (tetanus toxoid conjugate); HB – Hepatitis B vaccine; IPV – Inactivated polio vaccine; OPV – Oral polio vaccine; MenC – Meningococcal C vaccine; TT – tetanus toxoid conjugate; NA: not applicable; enr 3-6 m, 4-8 w int: enrolment at 3-6 months of age, and at 4-8 weeks interval of a 3 doses primary vaccines in total.

Each solid line in the figure shows the GMR from each trial. Black boxes and lines show the point estimates and confidence intervals for geometric mean ratios comparing vac1 vs vac2. Concomitant vaccines are vaccines co-administered with PCV primary vaccine series. Information on co-administered vaccine is not always available (e.g. study ID 11 and 23). Concomitant vaccines in the bracket are those administered in some but not all of the study sites.

Supplementary Figure 9. Trial level geometric mean ratios for serotype 19F post-primary vaccination series.


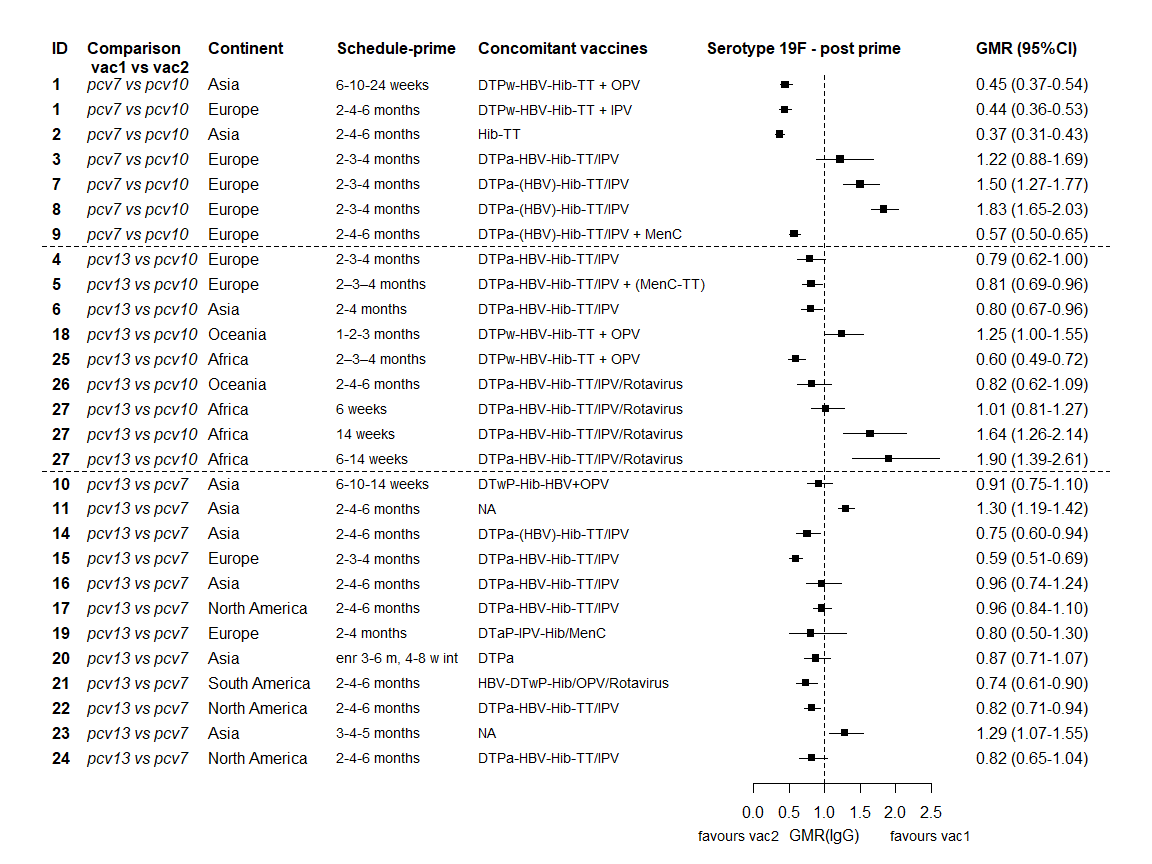


GMR: Geometric mean ratio; pcv: Pneumococcal conjugate vaccine; DTaP – diphtheria and tetanus toxoids, and acellular pertussis vaccine; DTwP – diphtheria and tetanus toxoids, and whole-cell pertussis vaccine; Hib-TT – Haemophilus influenzae type b vaccine (tetanus toxoid conjugate); HB – Hepatitis B vaccine; IPV – Inactivated polio vaccine; OPV – Oral polio vaccine; MenC – Meningococcal C vaccine; TT – tetanus toxoid conjugate; NA: not applicable; enr 3-6 m, 4-8 w int: enrolment at 3-6 months of age, and at 4-8 weeks interval of a 3 doses primary vaccines in total.

Each solid line in the figure shows the GMR from each trial. Black boxes and lines show the point estimates and confidence intervals for geometric mean ratios comparing vac1 vs vac2. Concomitant vaccines are vaccines co-administered with PCV primary vaccine series. Information on co-administered vaccine is not always available (e.g. study ID 11 and 23). Concomitant vaccines in the bracket are those administered in some but not all of the study sites.

Supplementary Figure 10. Trial level geometric mean ratios for serotype 23F post-primary vaccination series.


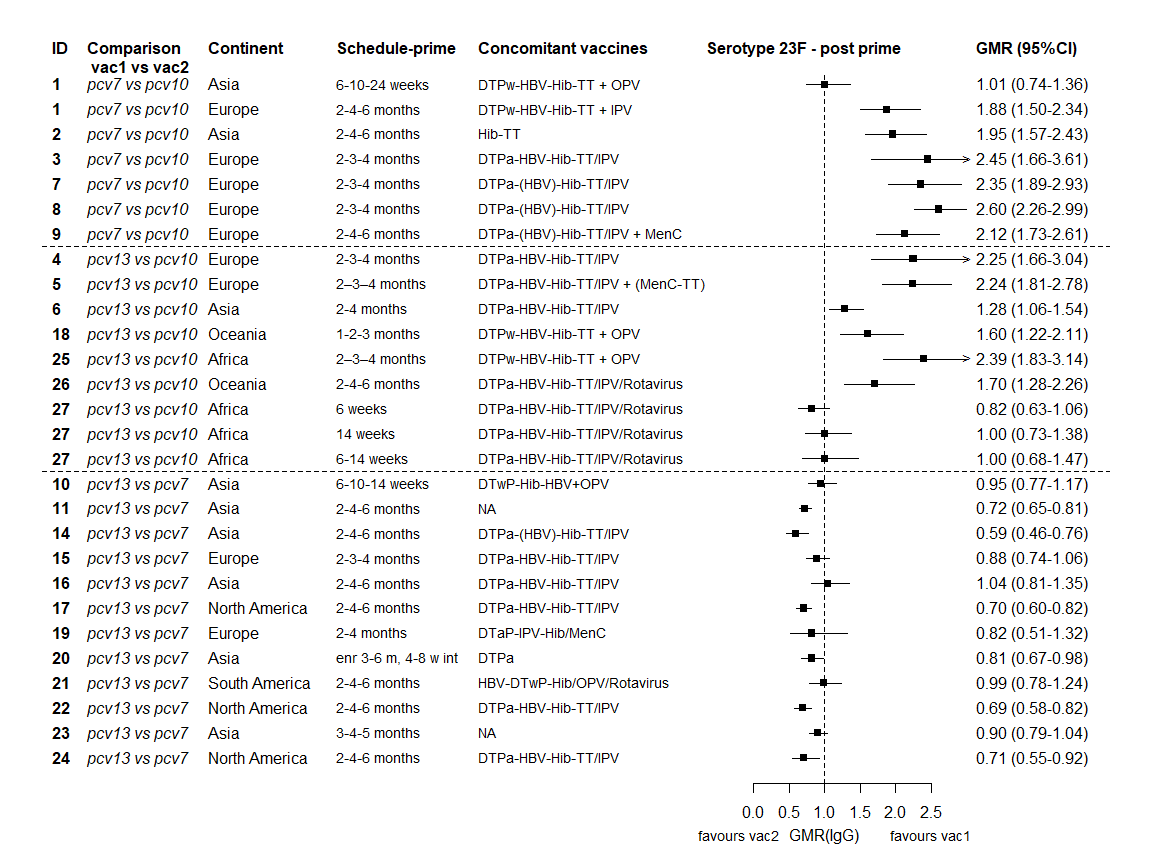


GMR: Geometric mean ratio; pcv: Pneumococcal conjugate vaccine; DTaP – diphtheria and tetanus toxoids, and acellular pertussis vaccine; DTwP – diphtheria and tetanus toxoids, and whole-cell pertussis vaccine; Hib-TT – Haemophilus influenzae type b vaccine (tetanus toxoid conjugate); HB – Hepatitis B vaccine; IPV – Inactivated polio vaccine; OPV – Oral polio vaccine; MenC – Meningococcal C vaccine; TT – tetanus toxoid conjugate; NA: not applicable; enr 3-6 m, 4-8 w int: enrolment at 3-6 months of age, and at 4-8 weeks interval of a 3 doses primary vaccines in total.

Each solid line in the figure shows the GMR from each trial. Black boxes and lines show the point estimates and confidence intervals for geometric mean ratios comparing vac1 vs vac2. Concomitant vaccines are vaccines co-administered with PCV primary vaccine series. Information on co-administered vaccine is not always available (e.g. study ID 11 and 23). Concomitant vaccines in the bracket are those administered in some but not all of the study sites.

Supplementary Figure 11. Trial level geometric mean ratios for serotype 1 post-primary vaccination series.


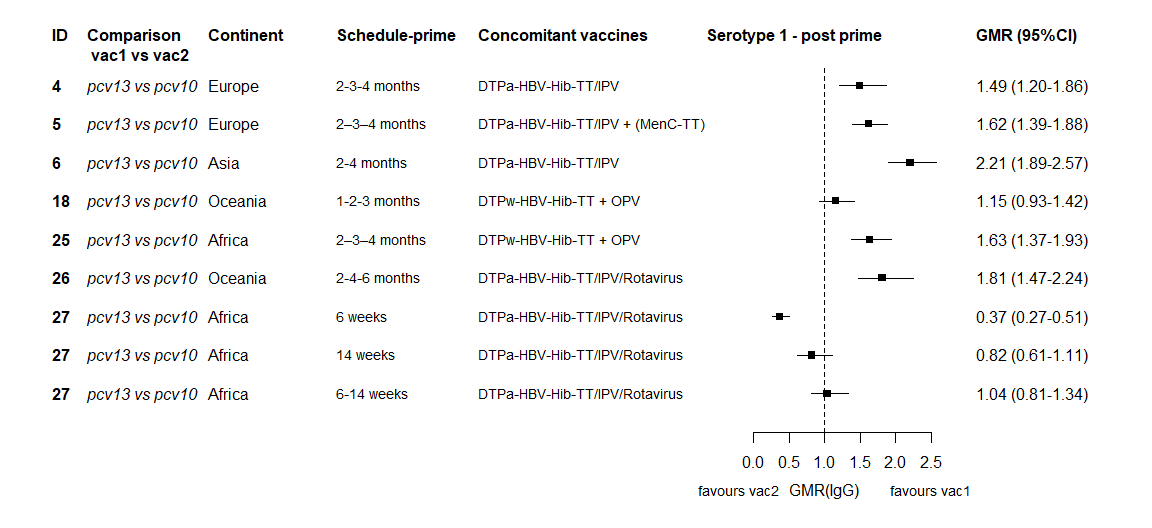
GMR: Geometric mean ratio; pcv: Pneumococcal conjugate vaccine; DTaP – diphtheria and tetanus toxoids, and acellular pertussis vaccine; DTwP – diphtheria and tetanus toxoids, and whole-cell pertussis vaccine; Hib-TT – Haemophilus influenzae type b vaccine (tetanus toxoid conjugate); HB – Hepatitis B vaccine; IPV – Inactivated polio vaccine; OPV – Oral polio vaccine; MenC – Meningococcal C vaccine; TT – tetanus toxoid conjugate; NA: not applicable; enr 3-6 m, 4-8 w int: enrolment at 3-6 months of age, and at 4-8 weeks interval of a 3 doses primary vaccines in total.

Each solid line in the figure shows the GMR from each trial. Black boxes and lines show the point estimates and confidence intervals for geometric mean ratios comparing vac1 vs vac2. Concomitant vaccines are vaccines co-administered with PCV primary vaccine series. Information on co-administered vaccine is not always available. Concomitant vaccines in the bracket are those administered in some but not all of the study sites.

Supplementary Figure 12. Trial level geometric mean ratios for serotype 5 post-primary vaccination series.


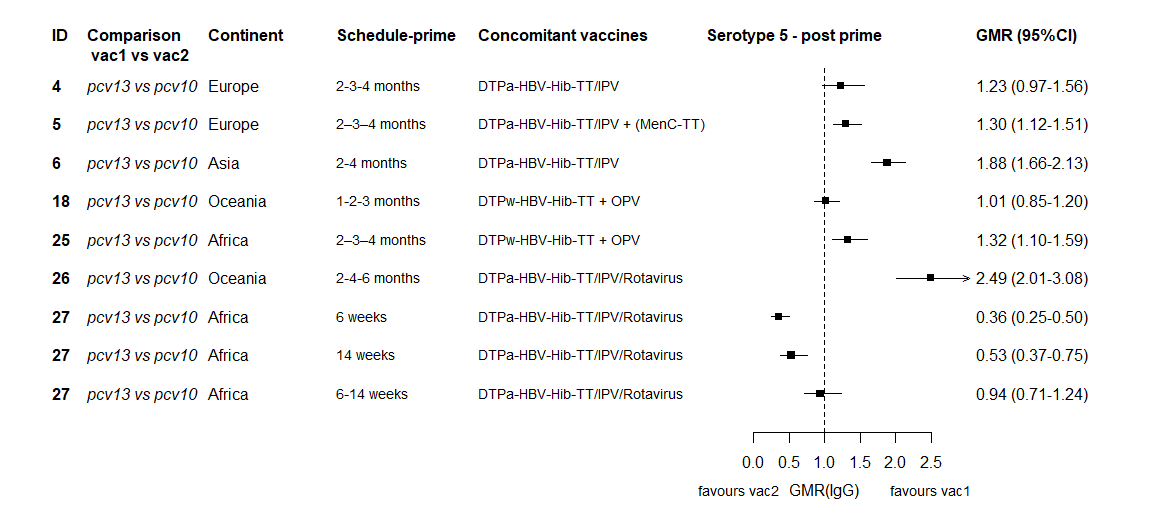


GMR: Geometric mean ratio; pcv: Pneumococcal conjugate vaccine; DTaP – diphtheria and tetanus toxoids, and acellular pertussis vaccine; DTwP – diphtheria and tetanus toxoids, and whole-cell pertussis vaccine; Hib-TT – Haemophilus influenzae type b vaccine (tetanus toxoid conjugate); HB – Hepatitis B vaccine; IPV – Inactivated polio vaccine; OPV – Oral polio vaccine; MenC – Meningococcal C vaccine; TT – tetanus toxoid conjugate; NA: not applicable; enr 3-6 m, 4-8 w int: enrolment at 3-6 months of age, and at 4-8 weeks interval of a 3 doses primary vaccines in total.

Each solid line in the figure shows the GMR from each trial. Black boxes and lines show the point estimates and confidence intervals for geometric mean ratios comparing vac1 vs vac2. Concomitant vaccines are vaccines co-administered with PCV primary vaccine series. Information on co-administered vaccine is not always available. Concomitant vaccines in the bracket are those administered in some but not all of the study sites.

Supplementary Figure 13. Trial level geometric mean ratios for serotype 7F post-primary vaccination series.


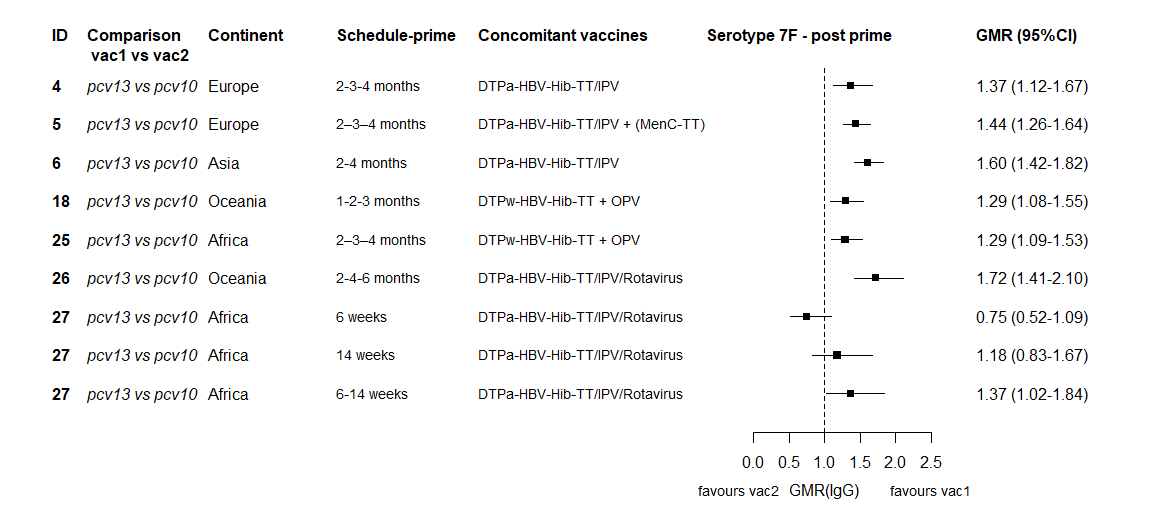


GMR: Geometric mean ratio; pcv: Pneumococcal conjugate vaccine; DTaP – diphtheria and tetanus toxoids, and acellular pertussis vaccine; DTwP – diphtheria and tetanus toxoids, and whole-cell pertussis vaccine; Hib-TT – Haemophilus influenzae type b vaccine (tetanus toxoid conjugate); HB – Hepatitis B vaccine; IPV – Inactivated polio vaccine; OPV – Oral polio vaccine; MenC – Meningococcal C vaccine; TT – tetanus toxoid conjugate; NA: not applicable; enr 3-6 m, 4-8 w int: enrolment at 3-6 months of age, and at 4-8 weeks interval of a 3 doses primary vaccines in total.

Each solid line in the figure shows the GMR from each trial. Black boxes and lines show the point estimates and confidence intervals for geometric mean ratios comparing vac1 vs vac2. Concomitant vaccines are vaccines co-administered with PCV primary vaccine series. Information on co-administered vaccine is not always available. Concomitant vaccines in the bracket are those administered in some but not all of the study sites.

Supplementary Figure 14. Trial level geometric mean ratios for serotype 3 post-primary vaccination series.


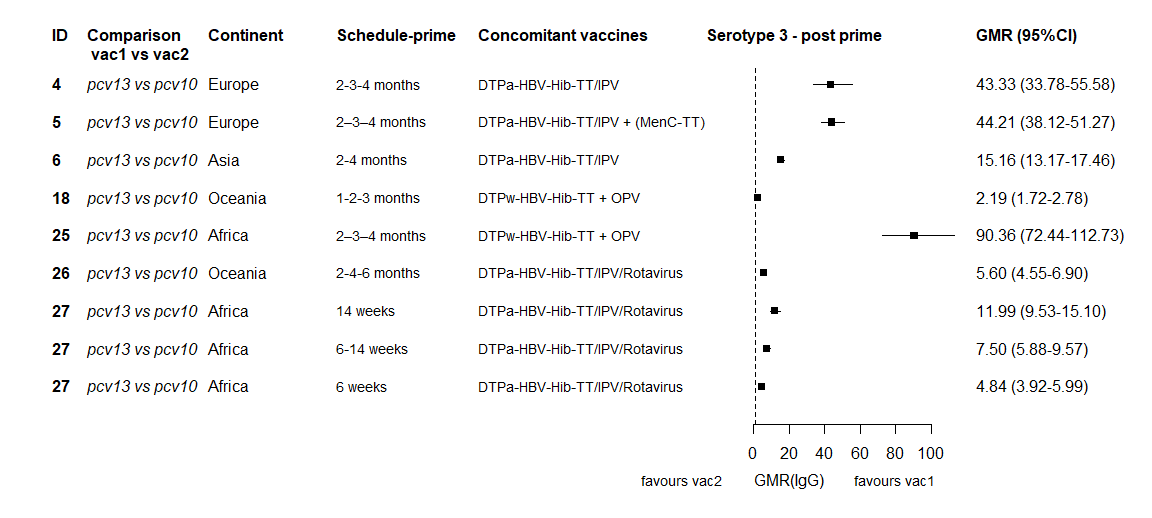


GMR: Geometric mean ratio; pcv: Pneumococcal conjugate vaccine; DTaP – diphtheria and tetanus toxoids, and acellular pertussis vaccine; DTwP – diphtheria and tetanus toxoids, and whole-cell pertussis vaccine; Hib-TT – Haemophilus influenzae type b vaccine (tetanus toxoid conjugate); HB – Hepatitis B vaccine; IPV – Inactivated polio vaccine; OPV – Oral polio vaccine; MenC – Meningococcal C vaccine; TT – tetanus toxoid conjugate; NA: not applicable; enr 3-6 m, 4-8 w int: enrolment at 3-6 months of age, and at 4-8 weeks interval of a 3 doses primary vaccines in total.

Each solid line in the figure shows the GMR from each trial. Black boxes and lines show the point estimates and confidence intervals for geometric mean ratios comparing vac1 vs vac2. Concomitant vaccines are vaccines co-administered with PCV primary vaccine series. Information on co-administered vaccine is not always available. Concomitant vaccines in the bracket are those administered in some but not all of the study sites.

Supplementary Figure 15. Trial level geometric mean ratios for serotype 6A post-primary vaccination series.


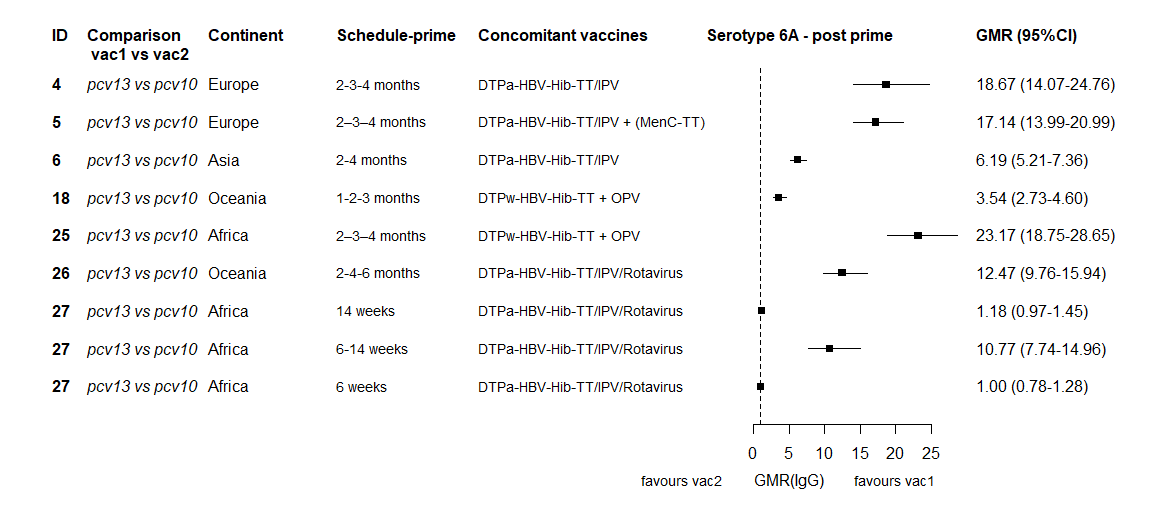


GMR: Geometric mean ratio; pcv: Pneumococcal conjugate vaccine; DTaP – diphtheria and tetanus toxoids, and acellular pertussis vaccine; DTwP – diphtheria and tetanus toxoids, and whole-cell pertussis vaccine; Hib-TT – Haemophilus influenzae type b vaccine (tetanus toxoid conjugate); HB – Hepatitis B vaccine; IPV – Inactivated polio vaccine; OPV – Oral polio vaccine; MenC – Meningococcal C vaccine; TT – tetanus toxoid conjugate; NA: not applicable; enr 3-6 m, 4-8 w int: enrolment at 3-6 months of age, and at 4-8 weeks interval of a 3 doses primary vaccines in total.

Each solid line in the figure shows the GMR from each trial. Black boxes and lines show the point estimates and confidence intervals for geometric mean ratios comparing vac1 vs vac2. Concomitant vaccines are vaccines co-administered with PCV primary vaccine series. Information on co-administered vaccine is not always available. Concomitant vaccines in the bracket are those administered in some but not all of the study sites.

Supplementary Figure 16. Trial level geometric mean ratios for serotype 19A post-primary vaccination series.


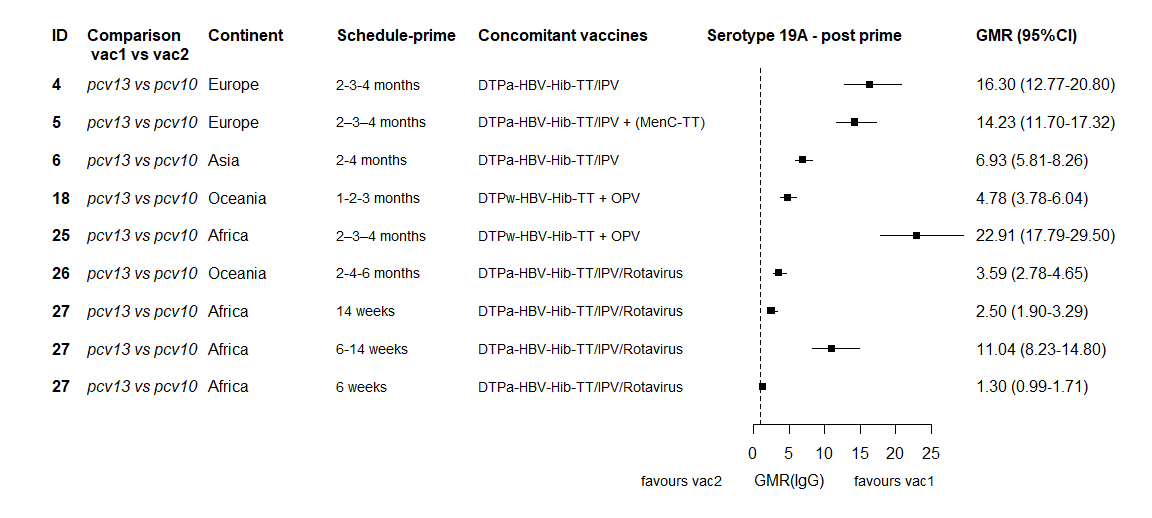


GMR: Geometric mean ratio; pcv: Pneumococcal conjugate vaccine; DTaP – diphtheria and tetanus toxoids, and acellular pertussis vaccine; DTwP – diphtheria and tetanus toxoids, and whole-cell pertussis vaccine; Hib-TT – Haemophilus influenzae type b vaccine (tetanus toxoid conjugate); HB – Hepatitis B vaccine; IPV – Inactivated polio vaccine; OPV – Oral polio vaccine; MenC – Meningococcal C vaccine; TT – tetanus toxoid conjugate; NA: not applicable; enr 3-6 m, 4-8 w int: enrolment at 3-6 months of age, and at 4-8 weeks interval of a 3 doses primary vaccines in total.

Each solid line in the figure shows the GMR from each trial. Black boxes and lines show the point estimates and confidence intervals for geometric mean ratios comparing vac1 vs vac2. Concomitant vaccines are vaccines co-administered with PCV primary vaccine series. Information on co-administered vaccine is not always available. Concomitant vaccines in the bracket are those administered in some but not all of the study sites.

Supplementary Figure 17. Trial level geometric mean ratios for serotype 4 pre-booster.


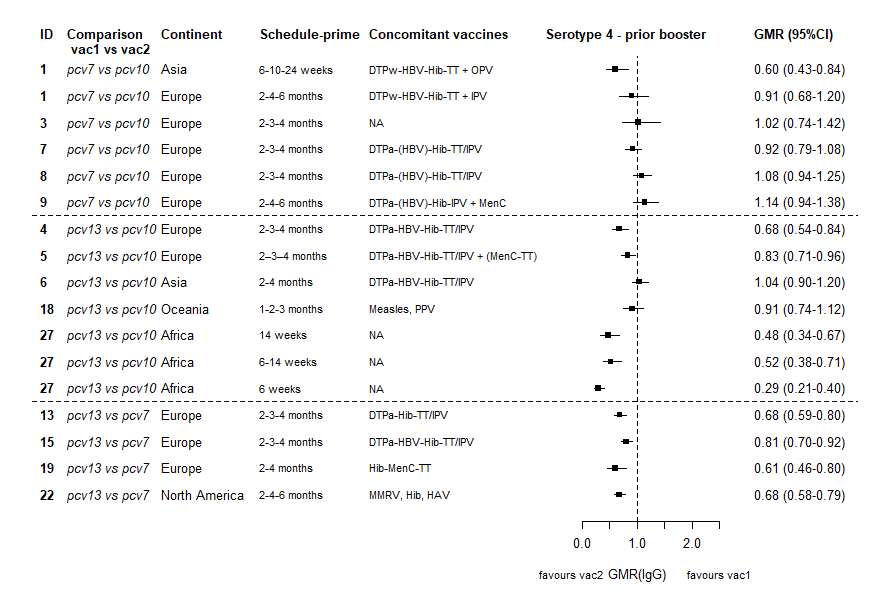


GMR: Geometric mean ratio; pcv: Pneumococcal conjugate vaccine; DTaP – diphtheria and tetanus toxoids, and acellular pertussis vaccine; DTwP – diphtheria and tetanus toxoids, and whole-cell pertussis vaccine; Hib-TT – Haemophilus influenzae type b vaccine (tetanus toxoid conjugate); HB – Hepatitis B vaccine; IPV – Inactivated polio vaccine; OPV – Oral polio vaccine; MenC – Meningococcal C vaccine; TT – tetanus toxoid conjugate; NA: not applicable; enr 3-6 m, 4-8 w int: enrolment at 3-6 months of age, and at 4-8 weeks interval of a 3 doses primary vaccines in total.

Each solid line in the figure shows the GMR from each trial. Black boxes and lines show the point estimates and confidence intervals for geometric mean ratios comparing vac1 vs vac2. Concomitant vaccines are vaccines co-administered with PCV primary vaccine series. Information on co-administered vaccine is not always available. Concomitant vaccines in the bracket are those administered in some but not all of the study sites.

Supplementary Figure 18. Trial level geometric mean ratios for serotype 6B pre-booster.


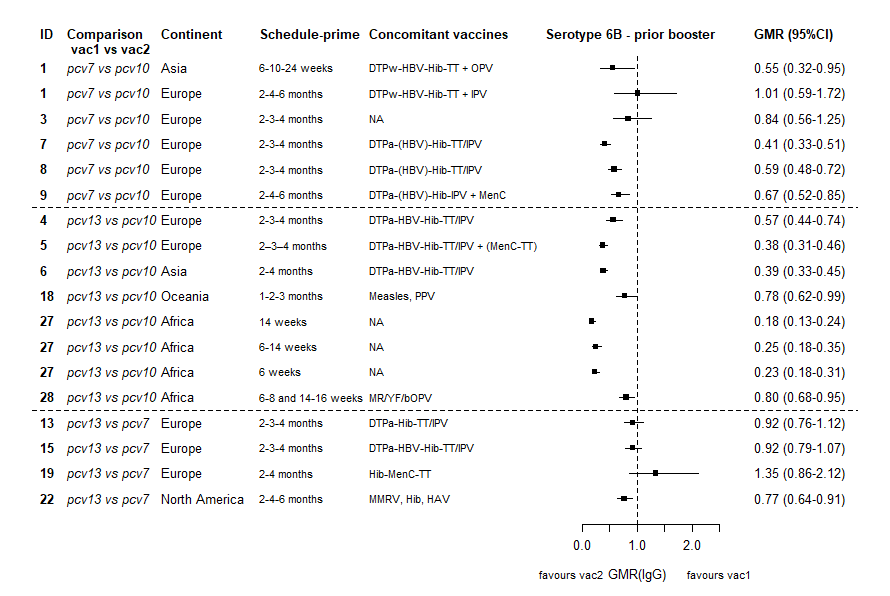


GMR: Geometric mean ratio; pcv: Pneumococcal conjugate vaccine; DTaP – diphtheria and tetanus toxoids, and acellular pertussis vaccine; DTwP – diphtheria and tetanus toxoids, and whole-cell pertussis vaccine; Hib-TT – Haemophilus influenzae type b vaccine (tetanus toxoid conjugate); HB – Hepatitis B vaccine; IPV – Inactivated polio vaccine; OPV – Oral polio vaccine; MenC – Meningococcal C vaccine; TT – tetanus toxoid conjugate; NA: not applicable; enr 3-6 m, 4-8 w int: enrolment at 3-6 months of age, and at 4-8 weeks interval of a 3 doses primary vaccines in total. MR: Measles and rubella combined vaccine; YF: Yellow fever vaccine; bOPV: Bivalent oral poliovirus vaccine

Each solid line in the figure shows the GMR from each trial. Black boxes and lines show the point estimates and confidence intervals for geometric mean ratios comparing vac1 vs vac2. Concomitant vaccines are vaccines co-administered with PCV primary vaccine series. Information on co-administered vaccine is not always available. Concomitant vaccines in the bracket are those administered in some but not all of the study sites.

Supplementary Figure 19. Trial level geometric mean ratios for serotype 9V pre-booster.


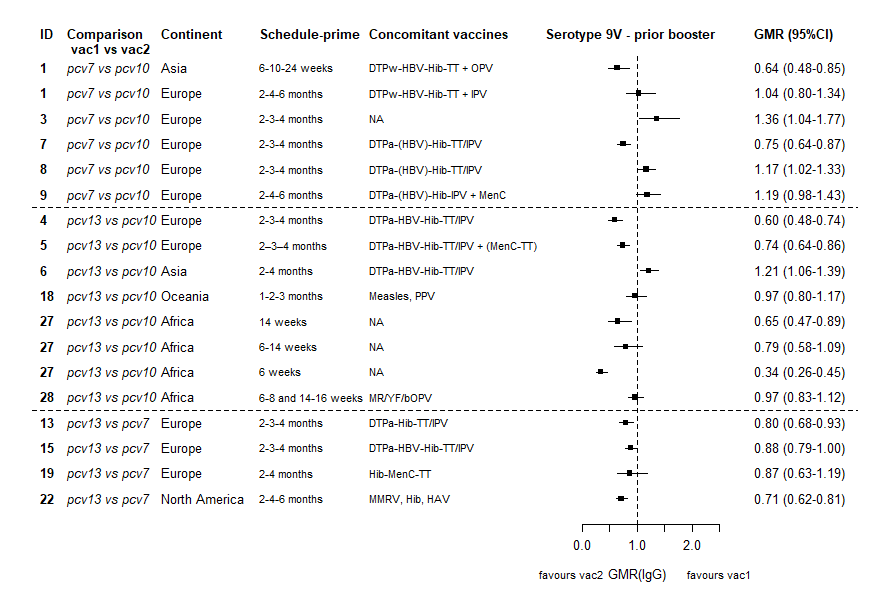


GMR: Geometric mean ratio; pcv: Pneumococcal conjugate vaccine; DTaP – diphtheria and tetanus toxoids, and acellular pertussis vaccine; DTwP – diphtheria and tetanus toxoids, and whole-cell pertussis vaccine; Hib-TT – Haemophilus influenzae type b vaccine (tetanus toxoid conjugate); HB – Hepatitis B vaccine; IPV – Inactivated polio vaccine; OPV – Oral polio vaccine; MenC – Meningococcal C vaccine; TT – tetanus toxoid conjugate; NA: not applicable; enr 3-6 m, 4-8 w int: enrolment at 3-6 months of age, and at 4-8 weeks interval of a 3 doses primary vaccines in total. MR: Measles and rubella combined vaccine; YF: Yellow fever vaccine; bOPV: Bivalent oral poliovirus vaccine

Each solid line in the figure shows the GMR from each trial. Black boxes and lines show the point estimates and confidence intervals for geometric mean ratios comparing vac1 vs vac2. Concomitant vaccines are vaccines co-administered with PCV primary vaccine series. Information on co-administered vaccine is not always available. Concomitant vaccines in the bracket are those administered in some but not all of the study sites.

Supplementary Figure 20. Trial level geometric mean ratios for serotype 14 pre-booster.


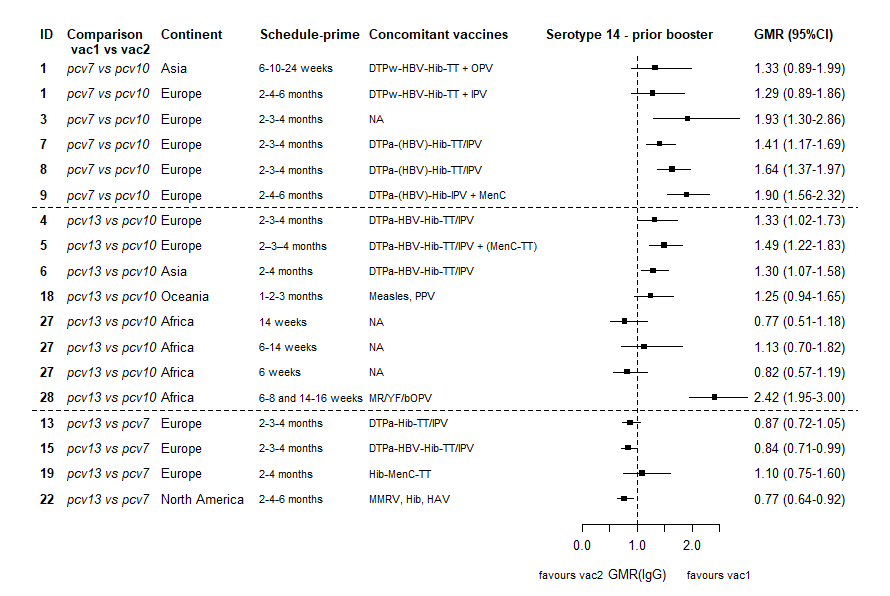


GMR: Geometric mean ratio; pcv: Pneumococcal conjugate vaccine; DTaP – diphtheria and tetanus toxoids, and acellular pertussis vaccine; DTwP – diphtheria and tetanus toxoids, and whole-cell pertussis vaccine; Hib-TT – Haemophilus influenzae type b vaccine (tetanus toxoid conjugate); HB – Hepatitis B vaccine; IPV – Inactivated polio vaccine; OPV – Oral polio vaccine; MenC – Meningococcal C vaccine; TT – tetanus toxoid conjugate; NA: not applicable; enr 3-6 m, 4-8 w int: enrolment at 3-6 months of age, and at 4-8 weeks interval of a 3 doses primary vaccines in total. MR: Measles and rubella combined vaccine; YF: Yellow fever vaccine; bOPV: Bivalent oral poliovirus vaccine

Each solid line in the figure shows the GMR from each trial. Black boxes and lines show the point estimates and confidence intervals for geometric mean ratios comparing vac1 vs vac2. Concomitant vaccines are vaccines co-administered with PCV primary vaccine series. Information on co-administered vaccine is not always available. Concomitant vaccines in the bracket are those administered in some but not all of the study sites.

Supplementary Figure 21. Trial level geometric mean ratios for serotype 18C pre-booster.


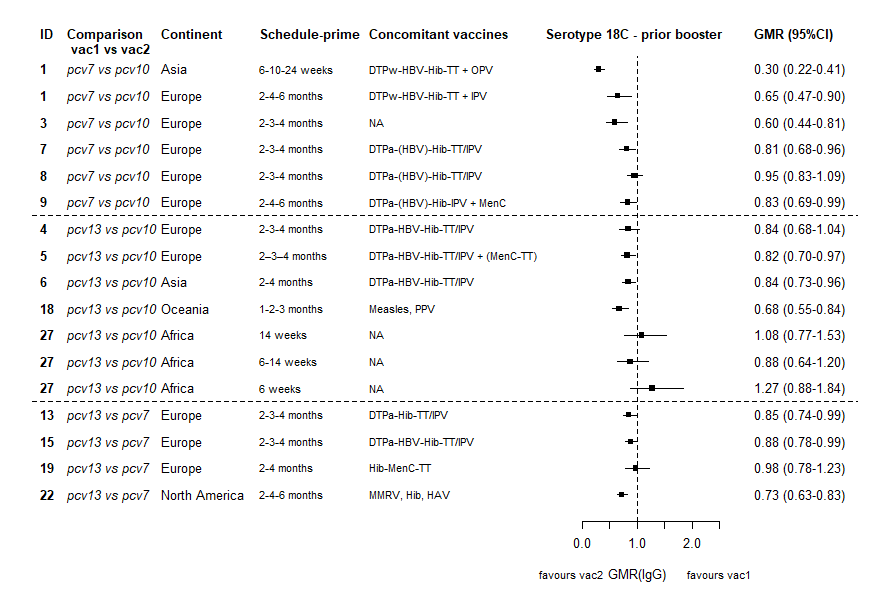


GMR: Geometric mean ratio; pcv: Pneumococcal conjugate vaccine; DTaP – diphtheria and tetanus toxoids, and acellular pertussis vaccine; DTwP – diphtheria and tetanus toxoids, and whole-cell pertussis vaccine; Hib-TT – Haemophilus influenzae type b vaccine (tetanus toxoid conjugate); HB – Hepatitis B vaccine; IPV – Inactivated polio vaccine; OPV – Oral polio vaccine; MenC – Meningococcal C vaccine; TT – tetanus toxoid conjugate; NA: not applicable; enr 3-6 m, 4-8 w int: enrolment at 3-6 months of age, and at 4-8 weeks interval of a 3 doses primary vaccines in total.

Each solid line in the figure shows the GMR from each trial. Black boxes and lines show the point estimates and confidence intervals for geometric mean ratios comparing vac1 vs vac2. Concomitant vaccines are vaccines co-administered with PCV primary vaccine series. Information on co-administered vaccine is not always available. Concomitant vaccines in the bracket are those administered in some but not all of the study sites.

Supplementary Figure 22. Trial level geometric mean ratios for serotype 19F pre-booster.


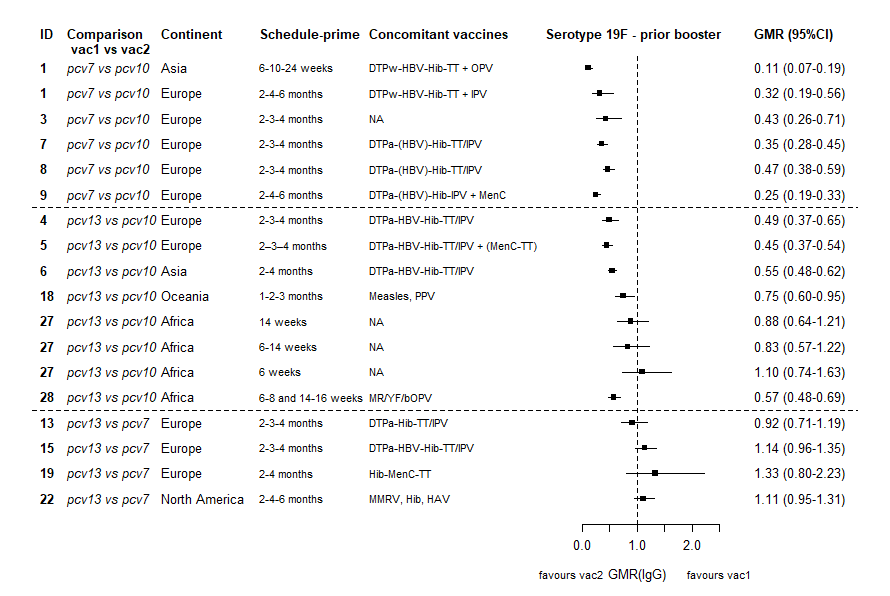


GMR: Geometric mean ratio; pcv: Pneumococcal conjugate vaccine; DTaP – diphtheria and tetanus toxoids, and acellular pertussis vaccine; DTwP – diphtheria and tetanus toxoids, and whole-cell pertussis vaccine; Hib-TT – Haemophilus influenzae type b vaccine (tetanus toxoid conjugate); HB – Hepatitis B vaccine; IPV – Inactivated polio vaccine; OPV – Oral polio vaccine; MenC – Meningococcal C vaccine; TT – tetanus toxoid conjugate; NA: not applicable; enr 3-6 m, 4-8 w int: enrolment at 3-6 months of age, and at 4-8 weeks interval of a 3 doses primary vaccines in total. MR: Measles and rubella combined vaccine; YF: Yellow fever vaccine; bOPV: Bivalent oral poliovirus vaccine

Each solid line in the figure shows the GMR from each trial. Black boxes and lines show the point estimates and confidence intervals for geometric mean ratios comparing vac1 vs vac2. Concomitant vaccines are vaccines co-administered with PCV primary vaccine series. Information on co-administered vaccine is not always available. Concomitant vaccines in the bracket are those administered in some but not all of the study sites.

Supplementary Figure 23. Trial level geometric mean ratios for serotype 23F pre-booster.


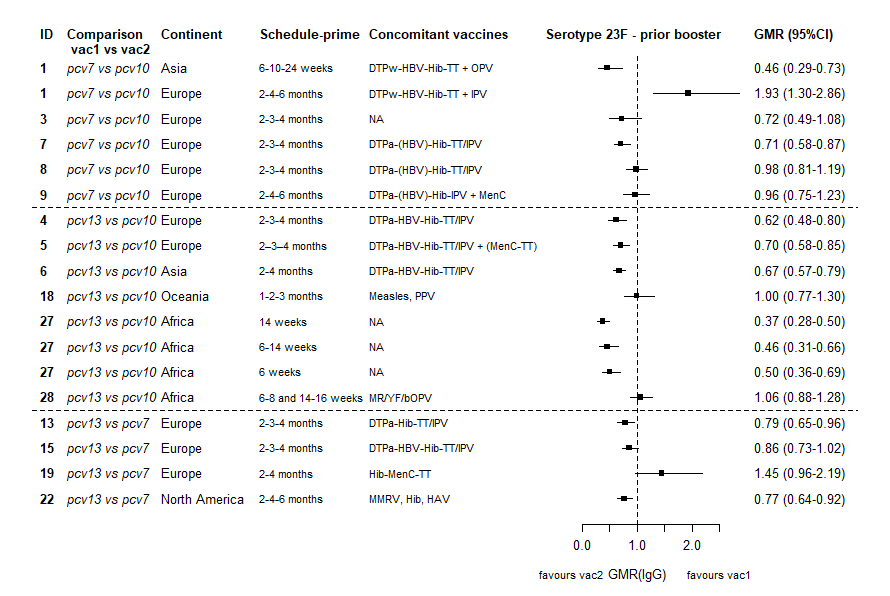


GMR: Geometric mean ratio; pcv: Pneumococcal conjugate vaccine; DTaP – diphtheria and tetanus toxoids, and acellular pertussis vaccine; DTwP – diphtheria and tetanus toxoids, and whole-cell pertussis vaccine; Hib-TT – Haemophilus influenzae type b vaccine (tetanus toxoid conjugate); HB – Hepatitis B vaccine; IPV – Inactivated polio vaccine; OPV – Oral polio vaccine; MenC – Meningococcal C vaccine; TT – tetanus toxoid conjugate; NA: not applicable; enr 3-6 m, 4-8 w int: enrolment at 3-6 months of age, and at 4-8 weeks interval of a 3 doses primary vaccines in total. MR: Measles and rubella combined vaccine; YF: Yellow fever vaccine; bOPV: Bivalent oral poliovirus vaccine

Each solid line in the figure shows the GMR from each trial. Black boxes and lines show the point estimates and confidence intervals for geometric mean ratios comparing vac1 vs vac2. Concomitant vaccines are vaccines co-administered with PCV primary vaccine series. Information on co-administered vaccine is not always available. Concomitant vaccines in the bracket are those administered in some but not all of the study sites.

Supplementary Figure 24. Trial level geometric mean ratios for serotype 1 pre-booster.


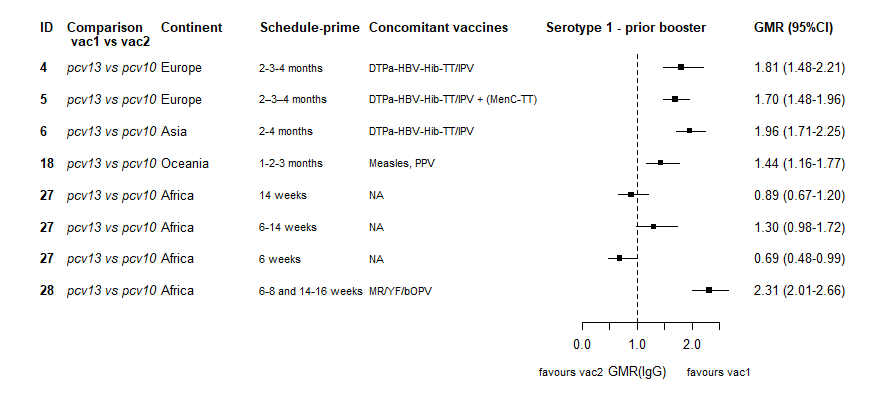


GMR: Geometric mean ratio; pcv: Pneumococcal conjugate vaccine; DTaP – diphtheria and tetanus toxoids, and acellular pertussis vaccine; DTwP – diphtheria and tetanus toxoids, and whole-cell pertussis vaccine; Hib-TT – Haemophilus influenzae type b vaccine (tetanus toxoid conjugate); HB – Hepatitis B vaccine; IPV – Inactivated polio vaccine; OPV – Oral polio vaccine; MenC – Meningococcal C vaccine; TT – tetanus toxoid conjugate; NA: not applicable; enr 3-6 m, 4-8 w int: enrolment at 3-6 months of age, and at 4-8 weeks interval of a 3 doses primary vaccines in total. MR: Measles and rubella combined vaccine; YF: Yellow fever vaccine; bOPV: Bivalent oral poliovirus vaccine

Each solid line in the figure shows the GMR from each trial. Black boxes and lines show the point estimates and confidence intervals for geometric mean ratios comparing vac1 vs vac2. Concomitant vaccines are vaccines co-administered with PCV primary vaccine series. Information on co-administered vaccine is not always available. Concomitant vaccines in the bracket are those administered in some but not all of the study sites.

Supplementary Figure 25. Trial level geometric mean ratios for serotype 5 pre-booster.


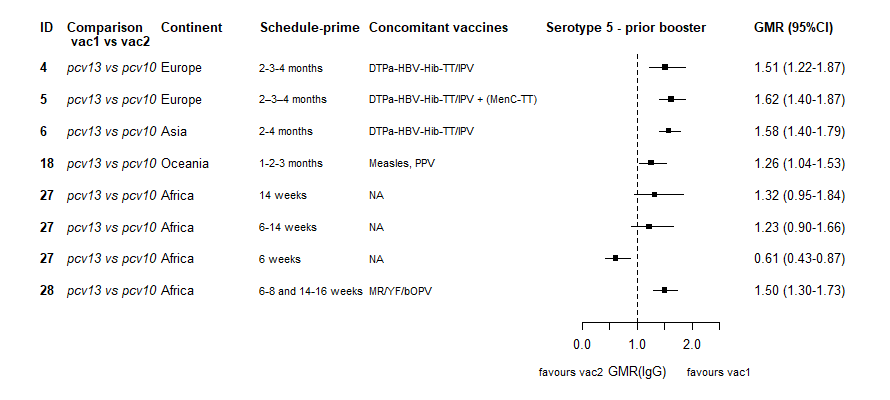


GMR: Geometric mean ratio; pcv: Pneumococcal conjugate vaccine; DTaP – diphtheria and tetanus toxoids, and acellular pertussis vaccine; DTwP – diphtheria and tetanus toxoids, and whole-cell pertussis vaccine; Hib-TT – Haemophilus influenzae type b vaccine (tetanus toxoid conjugate); HB – Hepatitis B vaccine; IPV – Inactivated polio vaccine; OPV – Oral polio vaccine; MenC – Meningococcal C vaccine; TT – tetanus toxoid conjugate; NA: not applicable; enr 3-6 m, 4-8 w int: enrolment at 3-6 months of age, and at 4-8 weeks interval of a 3 doses primary vaccines in total. MR: Measles and rubella combined vaccine; YF: Yellow fever vaccine; bOPV: Bivalent oral poliovirus vaccine

Each solid line in the figure shows the GMR from each trial. Black boxes and lines show the point estimates and confidence intervals for geometric mean ratios comparing vac1 vs vac2. Concomitant vaccines are vaccines co-administered with PCV primary vaccine series. Information on co-administered vaccine is not always available. Concomitant vaccines in the bracket are those administered in some but not all of the study sites.

Supplementary Figure 26. Trial level geometric mean ratios for serotype 7F pre-booster.


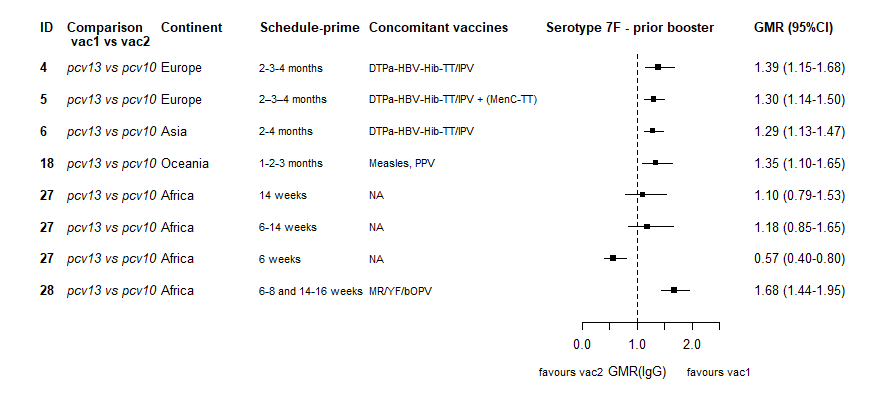


GMR: Geometric mean ratio; pcv: Pneumococcal conjugate vaccine; DTaP – diphtheria and tetanus toxoids, and acellular pertussis vaccine; DTwP – diphtheria and tetanus toxoids, and whole-cell pertussis vaccine; Hib-TT – Haemophilus influenzae type b vaccine (tetanus toxoid conjugate); HB – Hepatitis B vaccine; IPV – Inactivated polio vaccine; OPV – Oral polio vaccine; MenC – Meningococcal C vaccine; TT – tetanus toxoid conjugate; NA: not applicable; enr 3-6 m, 4-8 w int: enrolment at 3-6 months of age, and at 4-8 weeks interval of a 3 doses primary vaccines in total. MR: Measles and rubella combined vaccine; YF: Yellow fever vaccine; bOPV: Bivalent oral poliovirus vaccine

Each solid line in the figure shows the GMR from each trial. Black boxes and lines show the point estimates and confidence intervals for geometric mean ratios comparing vac1 vs vac2. Concomitant vaccines are vaccines co-administered with PCV primary vaccine series. Information on co-administered vaccine is not always available. Concomitant vaccines in the bracket are those administered in some but not all of the study sites.

Supplementary Figure 27. Trial level geometric mean ratios for serotype 3 pre-booster.


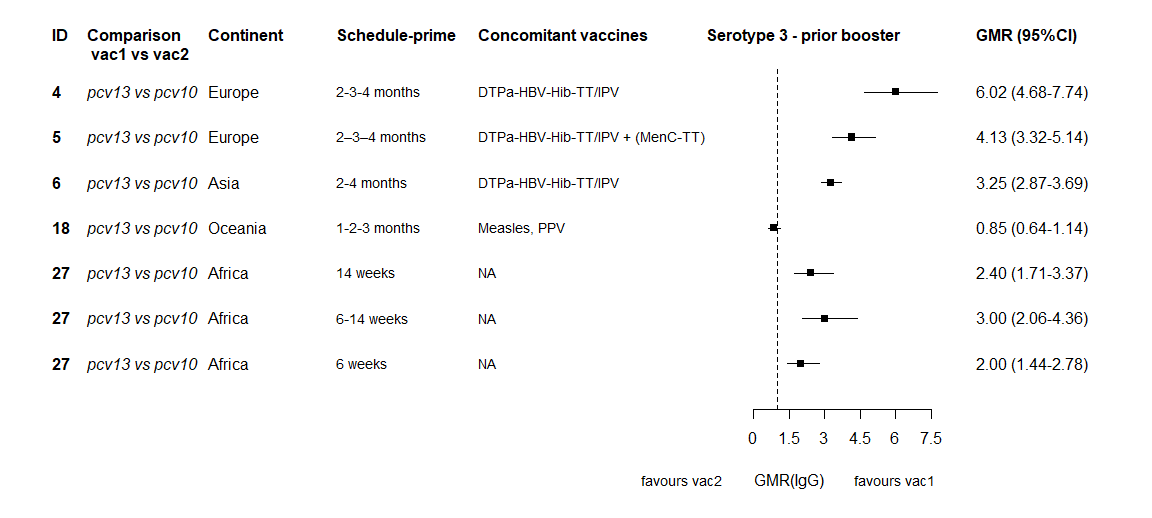


GMR: Geometric mean ratio; pcv: Pneumococcal conjugate vaccine; DTaP – diphtheria and tetanus toxoids, and acellular pertussis vaccine; DTwP – diphtheria and tetanus toxoids, and whole-cell pertussis vaccine; Hib-TT – Haemophilus influenzae type b vaccine (tetanus toxoid conjugate); HB – Hepatitis B vaccine; IPV – Inactivated polio vaccine; OPV – Oral polio vaccine; MenC – Meningococcal C vaccine; TT – tetanus toxoid conjugate; NA: not applicable; enr 3-6 m, 4-8 w int: enrolment at 3-6 months of age, and at 4-8 weeks interval of a 3 doses primary vaccines in total.

Each solid line in the figure shows the GMR from each trial. Black boxes and lines show the point estimates and confidence intervals for geometric mean ratios comparing vac1 vs vac2. Concomitant vaccines are vaccines co-administered with PCV primary vaccine series. Information on co-administered vaccine is not always available. Concomitant vaccines in the bracket are those administered in some but not all of the study sites.

Supplementary Figure 28. Trial level geometric mean ratios for serotype 6A pre-booster.


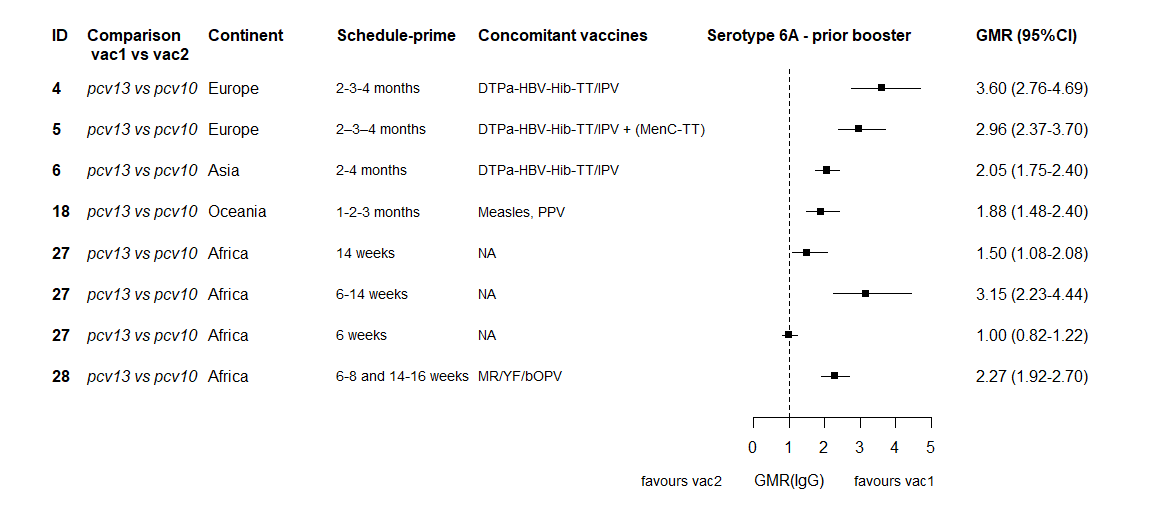


GMR: Geometric mean ratio; pcv: Pneumococcal conjugate vaccine; DTaP – diphtheria and tetanus toxoids, and acellular pertussis vaccine; DTwP – diphtheria and tetanus toxoids, and whole-cell pertussis vaccine; Hib-TT – Haemophilus influenzae type b vaccine (tetanus toxoid conjugate); HB – Hepatitis B vaccine; IPV – Inactivated polio vaccine; OPV – Oral polio vaccine; MenC – Meningococcal C vaccine; TT – tetanus toxoid conjugate; NA: not applicable; enr 3-6 m, 4-8 w int: enrolment at 3-6 months of age, and at 4-8 weeks interval of a 3 doses primary vaccines in total. MR: Measles and rubella combined vaccine; YF: Yellow fever vaccine; bOPV: Bivalent oral poliovirus vaccine

Each solid line in the figure shows the GMR from each trial. Black boxes and lines show the point estimates and confidence intervals for geometric mean ratios comparing vac1 vs vac2. Concomitant vaccines are vaccines co-administered with PCV primary vaccine series. Information on co-administered vaccine is not always available. Concomitant vaccines in the bracket are those administered in some but not all of the study sites.

Supplementary Figure 29. Trial level geometric mean ratios for serotype 19A pre-booster.


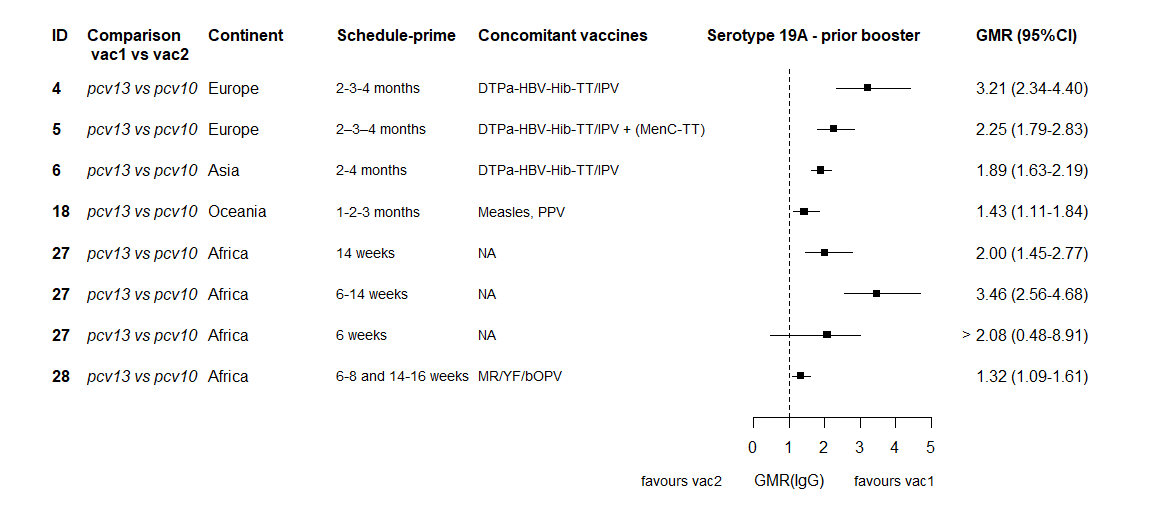


GMR: Geometric mean ratio; pcv: Pneumococcal conjugate vaccine; DTaP – diphtheria and tetanus toxoids, and acellular pertussis vaccine; DTwP – diphtheria and tetanus toxoids, and whole-cell pertussis vaccine; Hib-TT – Haemophilus influenzae type b vaccine (tetanus toxoid conjugate); HB – Hepatitis B vaccine; IPV – Inactivated polio vaccine; OPV – Oral polio vaccine; MenC – Meningococcal C vaccine; TT – tetanus toxoid conjugate; NA: not applicable; enr 3-6 m, 4-8 w int: enrolment at 3-6 months of age, and at 4-8 weeks interval of a 3 doses primary vaccines in total. MR: Measles and rubella combined vaccine; YF: Yellow fever vaccine; bOPV: Bivalent oral poliovirus vaccine

Each solid line in the figure shows the GMR from each trial. Black boxes and lines show the point estimates and confidence intervals for geometric mean ratios comparing vac1 vs vac2. Concomitant vaccines are vaccines co-administered with PCV primary vaccine series. Information on co-administered vaccine is not always available. Concomitant vaccines in the bracket are those administered in some but not all of the study sites.

Supplementary Figure 30. Trial level geometric mean ratios for serotype 4 post-booster.


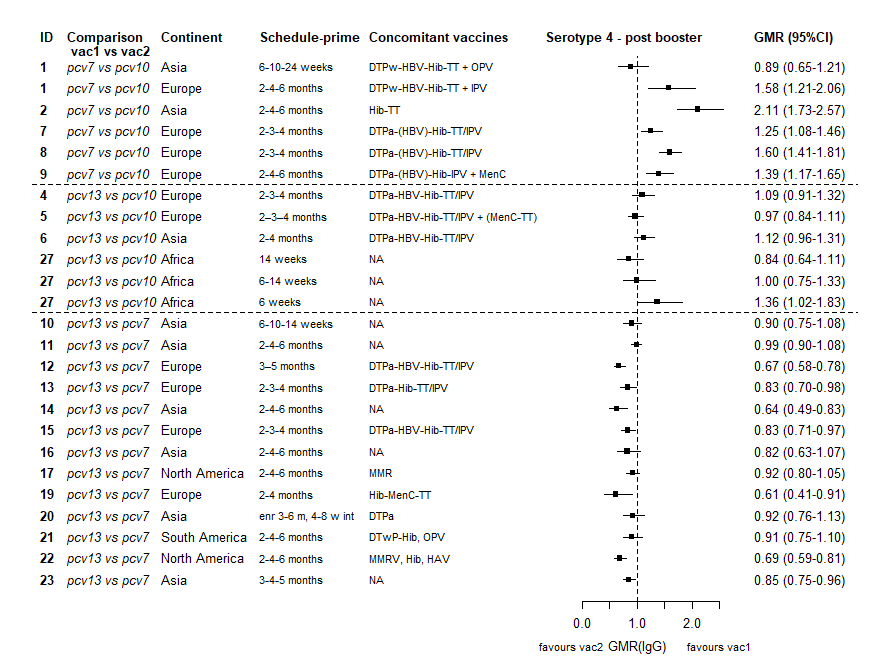


GMR: Geometric mean ratio; pcv: Pneumococcal conjugate vaccine; DTaP – diphtheria and tetanus toxoids, and acellular pertussis vaccine; DTwP – diphtheria and tetanus toxoids, and whole-cell pertussis vaccine; Hib-TT – Haemophilus influenzae type b vaccine (tetanus toxoid conjugate); HB – Hepatitis B vaccine; IPV – Inactivated polio vaccine; OPV – Oral polio vaccine; MenC – Meningococcal C vaccine; TT – tetanus toxoid conjugate; NA: not applicable; enr 3-6 m, 4-8 w int: enrolment at 3-6 months of age, and at 4-8 weeks interval of a 3 doses primary vaccines in total.

Each solid line in the figure shows the GMR from each trial. Black boxes and lines show the point estimates and confidence intervals for geometric mean ratios comparing vac1 vs vac2. Concomitant vaccines are vaccines co-administered with PCV primary vaccine series. Information on co-administered vaccine is not always available. Concomitant vaccines in the bracket are those administered in some but not all of the study sites.

Supplementary Figure 31. Trial level geometric mean ratios for serotype 6B post-booster.


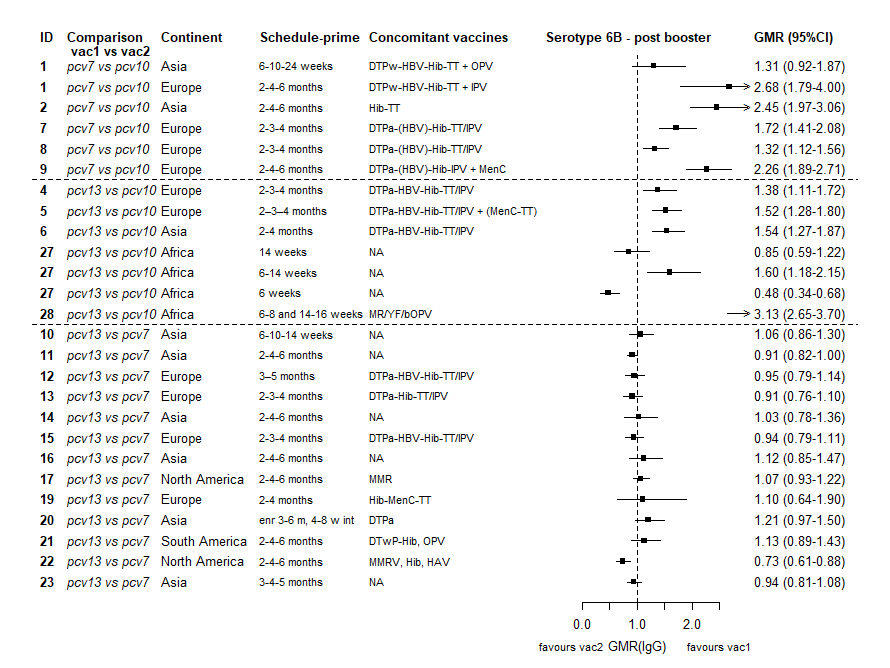


GMR: Geometric mean ratio; pcv: Pneumococcal conjugate vaccine; DTaP – diphtheria and tetanus toxoids, and acellular pertussis vaccine; DTwP – diphtheria and tetanus toxoids, and whole-cell pertussis vaccine; Hib-TT – Haemophilus influenzae type b vaccine (tetanus toxoid conjugate); HB – Hepatitis B vaccine; IPV – Inactivated polio vaccine; OPV – Oral polio vaccine; MenC – Meningococcal C vaccine; TT – tetanus toxoid conjugate; NA: not applicable; enr 3-6 m, 4-8 w int: enrolment at 3-6 months of age, and at 4-8 weeks interval of a 3 doses primary vaccines in total. MR: Measles and rubella combined vaccine; YF: Yellow fever vaccine; bOPV: Bivalent oral poliovirus vaccine

Each solid line in the figure shows the GMR from each trial. Black boxes and lines show the point estimates and confidence intervals for geometric mean ratios comparing vac1 vs vac2. Concomitant vaccines are vaccines co-administered with PCV primary vaccine series. Information on co-administered vaccine is not always available. Concomitant vaccines in the bracket are those administered in some but not all of the study sites.

Supplementary Figure 32. Trial level geometric mean ratios for serotype 9V post-booster.


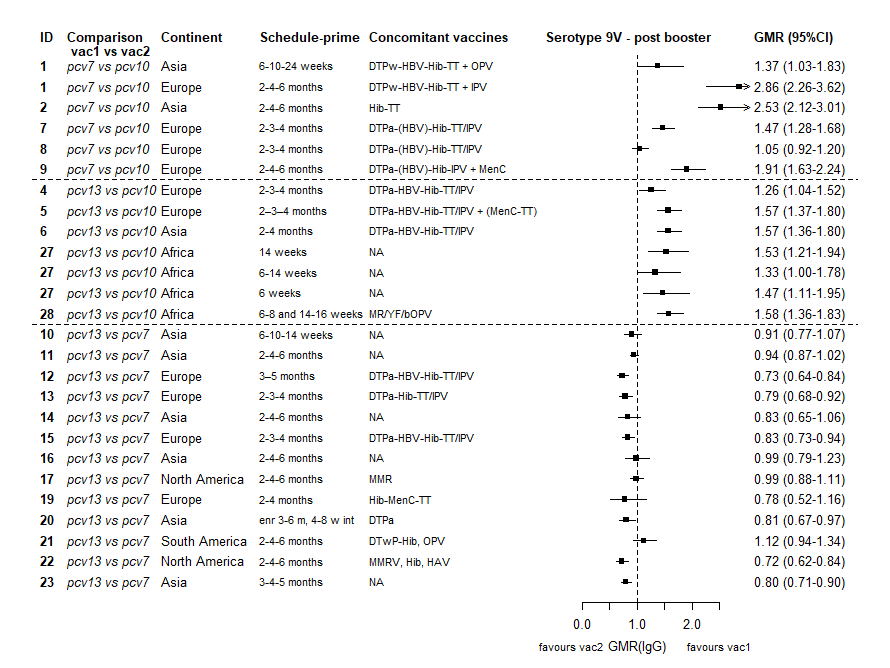


GMR: Geometric mean ratio; pcv: Pneumococcal conjugate vaccine; DTaP – diphtheria and tetanus toxoids, and acellular pertussis vaccine; DTwP – diphtheria and tetanus toxoids, and whole-cell pertussis vaccine; Hib-TT – Haemophilus influenzae type b vaccine (tetanus toxoid conjugate); HB – Hepatitis B vaccine; IPV – Inactivated polio vaccine; OPV – Oral polio vaccine; MenC – Meningococcal C vaccine; TT – tetanus toxoid conjugate; NA: not applicable; enr 3-6 m, 4-8 w int: enrolment at 3-6 months of age, and at 4-8 weeks interval of a 3 doses primary vaccines in total. MR: Measles and rubella combined vaccine; YF: Yellow fever vaccine; bOPV: Bivalent oral poliovirus vaccine

Each solid line in the figure shows the GMR from each trial. Black boxes and lines show the point estimates and confidence intervals for geometric mean ratios comparing vac1 vs vac2. Concomitant vaccines are vaccines co-administered with PCV primary vaccine series. Information on co-administered vaccine is not always available. Concomitant vaccines in the bracket are those administered in some but not all of the study sites.

Supplementary Figure 33. Trial level geometric mean ratios for serotype 14 post-booster.


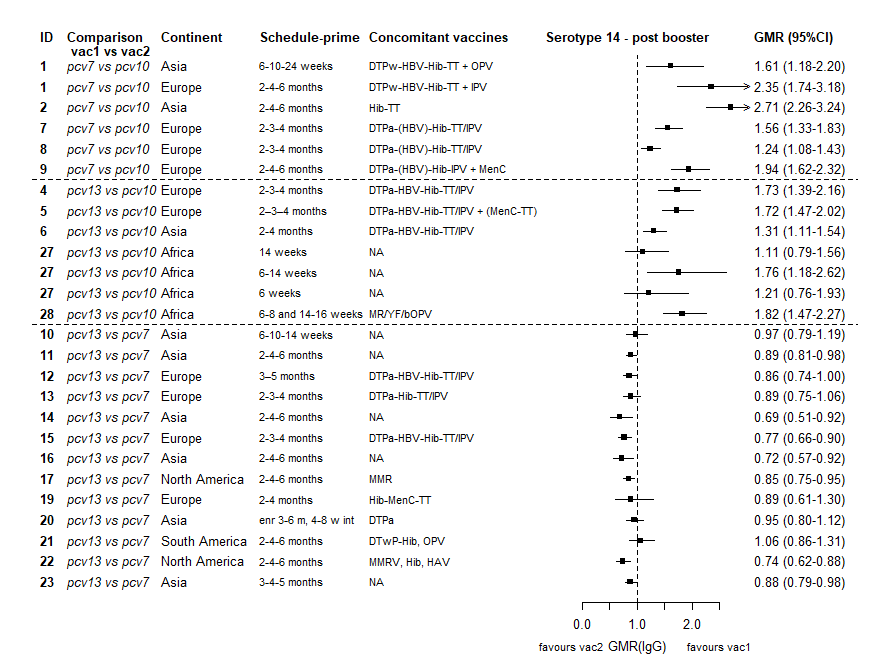


GMR: Geometric mean ratio; pcv: Pneumococcal conjugate vaccine; DTaP – diphtheria and tetanus toxoids, and acellular pertussis vaccine; DTwP – diphtheria and tetanus toxoids, and whole-cell pertussis vaccine; Hib-TT – Haemophilus influenzae type b vaccine (tetanus toxoid conjugate); HB – Hepatitis B vaccine; IPV – Inactivated polio vaccine; OPV – Oral polio vaccine; MenC – Meningococcal C vaccine; TT – tetanus toxoid conjugate; NA: not applicable; enr 3-6 m, 4-8 w int: enrolment at 3-6 months of age, and at 4-8 weeks interval of a 3 doses primary vaccines in total. MR: Measles and rubella combined vaccine; YF: Yellow fever vaccine; bOPV: Bivalent oral poliovirus vaccine

Each solid line in the figure shows the GMR from each trial. Black boxes and lines show the point estimates and confidence intervals for geometric mean ratios comparing vac1 vs vac2. Concomitant vaccines are vaccines co-administered with PCV primary vaccine series. Information on co-administered vaccine is not always available. Concomitant vaccines in the bracket are those administered in some but not all of the study sites.

Supplementary Figure 34. Trial level geometric mean ratios for serotype 18C post-booster.


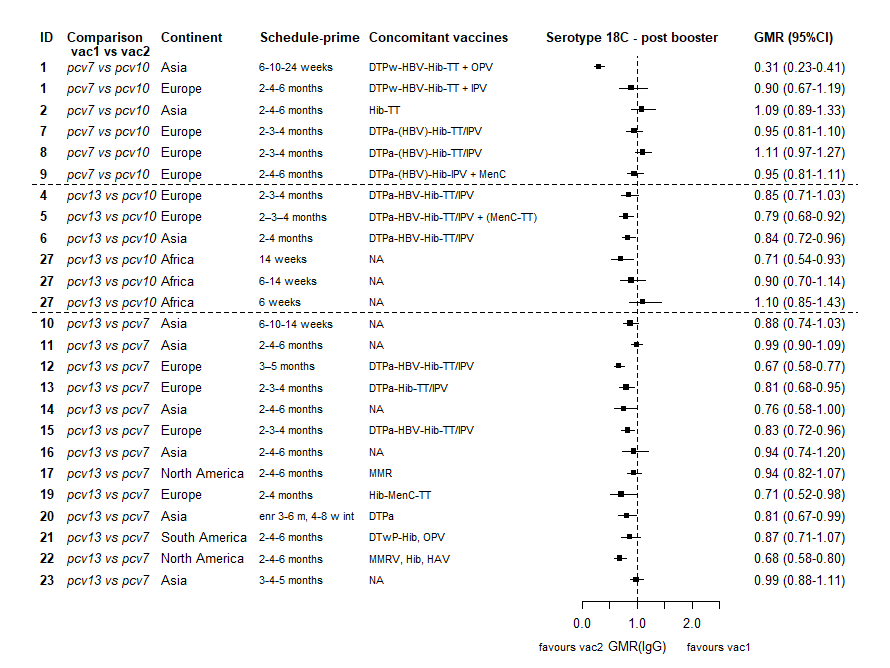


GMR: Geometric mean ratio; pcv: Pneumococcal conjugate vaccine; DTaP – diphtheria and tetanus toxoids, and acellular pertussis vaccine; DTwP – diphtheria and tetanus toxoids, and whole-cell pertussis vaccine; Hib-TT – Haemophilus influenzae type b vaccine (tetanus toxoid conjugate); HB – Hepatitis B vaccine; IPV – Inactivated polio vaccine; OPV – Oral polio vaccine; MenC – Meningococcal C vaccine; TT – tetanus toxoid conjugate; NA: not applicable; enr 3-6 m, 4-8 w int: enrolment at 3-6 months of age, and at 4-8 weeks interval of a 3 doses primary vaccines in total.

Each solid line in the figure shows the GMR from each trial. Black boxes and lines show the point estimates and confidence intervals for geometric mean ratios comparing vac1 vs vac2. Concomitant vaccines are vaccines co-administered with PCV primary vaccine series. Information on co-administered vaccine is not always available. Concomitant vaccines in the bracket are those administered in some but not all of the study sites.

Supplementary Figure 35. Trial level geometric mean ratios for serotype 19F post-booster.


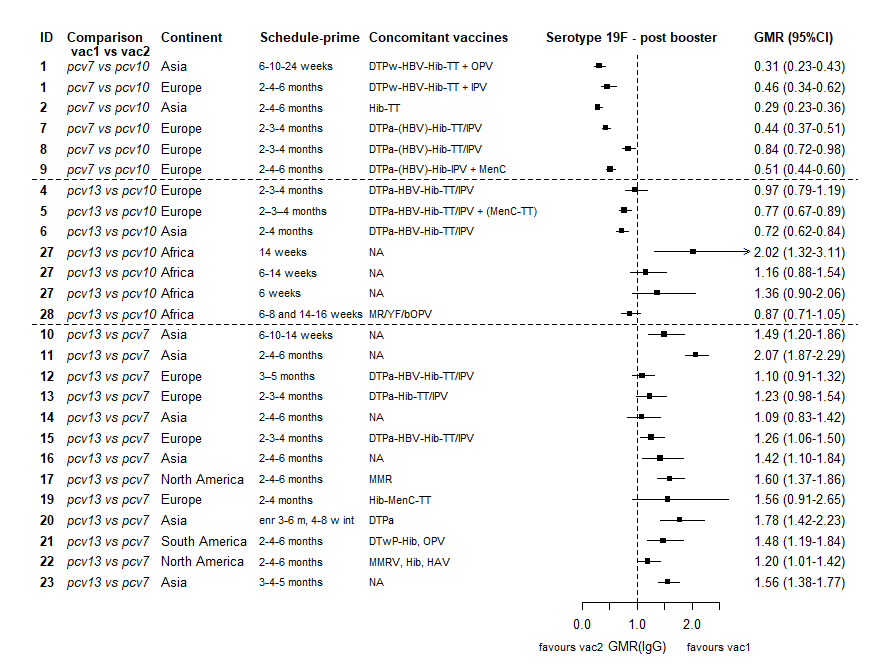


GMR: Geometric mean ratio; pcv: Pneumococcal conjugate vaccine; DTaP – diphtheria and tetanus toxoids, and acellular pertussis vaccine; DTwP – diphtheria and tetanus toxoids, and whole-cell pertussis vaccine; Hib-TT – Haemophilus influenzae type b vaccine (tetanus toxoid conjugate); HB – Hepatitis B vaccine; IPV – Inactivated polio vaccine; OPV – Oral polio vaccine; MenC – Meningococcal C vaccine; TT – tetanus toxoid conjugate; NA: not applicable; enr 3-6 m, 4-8 w int: enrolment at 3-6 months of age, and at 4-8 weeks interval of a 3 doses primary vaccines in total. MR: Measles and rubella combined vaccine; YF: Yellow fever vaccine; bOPV: Bivalent oral poliovirus vaccine

Each solid line in the figure shows the GMR from each trial. Black boxes and lines show the point estimates and confidence intervals for geometric mean ratios comparing vac1 vs vac2. Concomitant vaccines are vaccines co-administered with PCV primary vaccine series. Information on co-administered vaccine is not always available. Concomitant vaccines in the bracket are those administered in some but not all of the study sites.

Supplementary Figure 36. Trial level geometric mean ratios for serotype 23F post-booster.


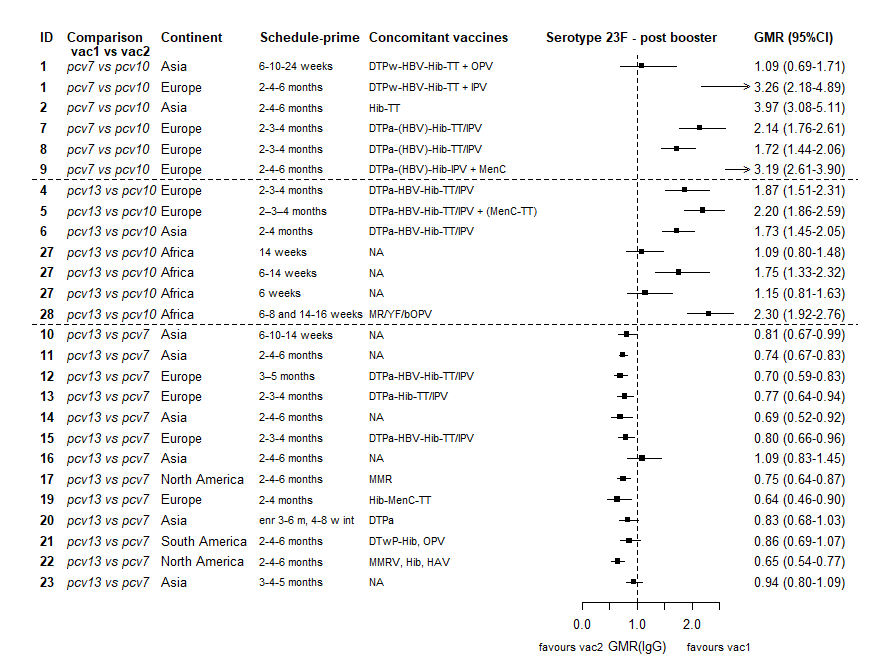


GMR: Geometric mean ratio; pcv: Pneumococcal conjugate vaccine; DTaP – diphtheria and tetanus toxoids, and acellular pertussis vaccine; DTwP – diphtheria and tetanus toxoids, and whole-cell pertussis vaccine; Hib-TT – Haemophilus influenzae type b vaccine (tetanus toxoid conjugate); HB – Hepatitis B vaccine; IPV – Inactivated polio vaccine; OPV – Oral polio vaccine; MenC – Meningococcal C vaccine; TT – tetanus toxoid conjugate; NA: not applicable; enr 3-6 m, 4-8 w int: enrolment at 3-6 months of age, and at 4-8 weeks interval of a 3 doses primary vaccines in total. MR: Measles and rubella combined vaccine; YF: Yellow fever vaccine; bOPV: Bivalent oral poliovirus vaccine

Each solid line in the figure shows the GMR from each trial. Black boxes and lines show the point estimates and confidence intervals for geometric mean ratios comparing vac1 vs vac2. Concomitant vaccines are vaccines co-administered with PCV primary vaccine series. Information on co-administered vaccine is not always available. Concomitant vaccines in the bracket are those administered in some but not all of the study sites.

Supplementary Figure 37. Trial level geometric mean ratios for serotype 1 post-booster.


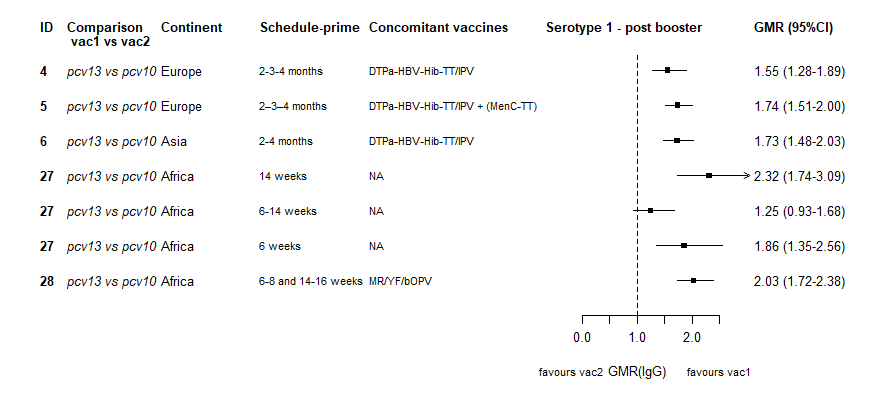


GMR: Geometric mean ratio; pcv: Pneumococcal conjugate vaccine; DTaP – diphtheria and tetanus toxoids, and acellular pertussis vaccine; DTwP – diphtheria and tetanus toxoids, and whole-cell pertussis vaccine; Hib-TT – Haemophilus influenzae type b vaccine (tetanus toxoid conjugate); HB – Hepatitis B vaccine; IPV – Inactivated polio vaccine; OPV – Oral polio vaccine; MenC – Meningococcal C vaccine; TT – tetanus toxoid conjugate; NA: not applicable; enr 3-6 m, 4-8 w int: enrolment at 3-6 months of age, and at 4-8 weeks interval of a 3 doses primary vaccines in total. MR: Measles and rubella combined vaccine; YF: Yellow fever vaccine; bOPV: Bivalent oral poliovirus vaccine

Each solid line in the figure shows the GMR from each trial. Black boxes and lines show the point estimates and confidence intervals for geometric mean ratios comparing vac1 vs vac2. Concomitant vaccines are vaccines co-administered with PCV primary vaccine series. Information on co-administered vaccine is not always available. Concomitant vaccines in the bracket are those administered in some but not all of the study sites.

Supplementary Figure 38. Trial level geometric mean ratios for serotype 5 post-booster.


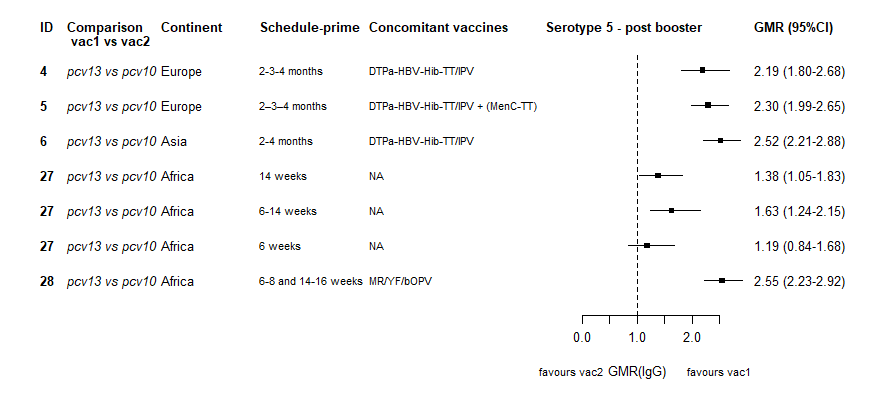


GMR: Geometric mean ratio; pcv: Pneumococcal conjugate vaccine; DTaP – diphtheria and tetanus toxoids, and acellular pertussis vaccine; DTwP – diphtheria and tetanus toxoids, and whole-cell pertussis vaccine; Hib-TT – Haemophilus influenzae type b vaccine (tetanus toxoid conjugate); HB – Hepatitis B vaccine; IPV – Inactivated polio vaccine; OPV – Oral polio vaccine; MenC – Meningococcal C vaccine; TT – tetanus toxoid conjugate; NA: not applicable; enr 3-6 m, 4-8 w int: enrolment at 3-6 months of age, and at 4-8 weeks interval of a 3 doses primary vaccines in total. MR: Measles and rubella combined vaccine; YF: Yellow fever vaccine; bOPV: Bivalent oral poliovirus vaccine

Each solid line in the figure shows the GMR from each trial. Black boxes and lines show the point estimates and confidence intervals for geometric mean ratios comparing vac1 vs vac2. Concomitant vaccines are vaccines co-administered with PCV primary vaccine series. Information on co-administered vaccine is not always available. Concomitant vaccines in the bracket are those administered in some but not all of the study sites.

Supplementary Figure 39. Trial level geometric mean ratios for serotype 7F post-booster.


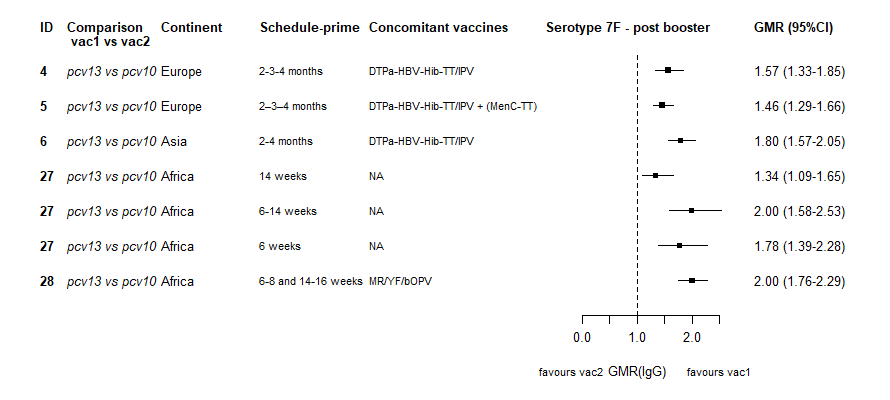


GMR: Geometric mean ratio; pcv: Pneumococcal conjugate vaccine; DTaP – diphtheria and tetanus toxoids, and acellular pertussis vaccine; DTwP – diphtheria and tetanus toxoids, and whole-cell pertussis vaccine; Hib-TT – Haemophilus influenzae type b vaccine (tetanus toxoid conjugate); HB – Hepatitis B vaccine; IPV – Inactivated polio vaccine; OPV – Oral polio vaccine; MenC – Meningococcal C vaccine; TT – tetanus toxoid conjugate; NA: not applicable; enr 3-6 m, 4-8 w int: enrolment at 3-6 months of age, and at 4-8 weeks interval of a 3 doses primary vaccines in total. MR: Measles and rubella combined vaccine; YF: Yellow fever vaccine; bOPV: Bivalent oral poliovirus vaccine

Each solid line in the figure shows the GMR from each trial. Black boxes and lines show the point estimates and confidence intervals for geometric mean ratios comparing vac1 vs vac2. Concomitant vaccines are vaccines co-administered with PCV primary vaccine series. Information on co-administered vaccine is not always available. Concomitant vaccines in the bracket are those administered in some but not all of the study sites.

Supplementary Figure 40. Trial level geometric mean ratios for serotype 3 post-booster.


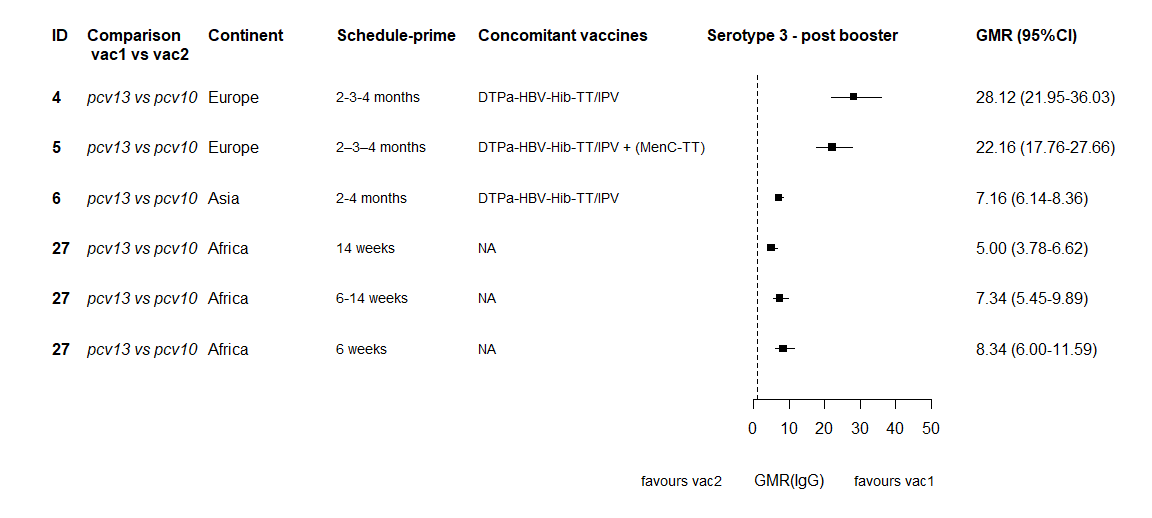


GMR: Geometric mean ratio; pcv: Pneumococcal conjugate vaccine; DTaP – diphtheria and tetanus toxoids, and acellular pertussis vaccine; DTwP – diphtheria and tetanus toxoids, and whole-cell pertussis vaccine; Hib-TT – Haemophilus influenzae type b vaccine (tetanus toxoid conjugate); HB – Hepatitis B vaccine; IPV – Inactivated polio vaccine; OPV – Oral polio vaccine; MenC – Meningococcal C vaccine; TT – tetanus toxoid conjugate; NA: not applicable; enr 3-6 m, 4-8 w int: enrolment at 3-6 months of age, and at 4-8 weeks interval of a 3 doses primary vaccines in total.

Each solid line in the figure shows the GMR from each trial. Black boxes and lines show the point estimates and confidence intervals for geometric mean ratios comparing vac1 vs vac2. Concomitant vaccines are vaccines co-administered with PCV primary vaccine series. Information on co-administered vaccine is not always available. Concomitant vaccines in the bracket are those administered in some but not all of the study sites.

Supplementary Figure 41. Trial level geometric mean ratios for serotype 6A post-booster.


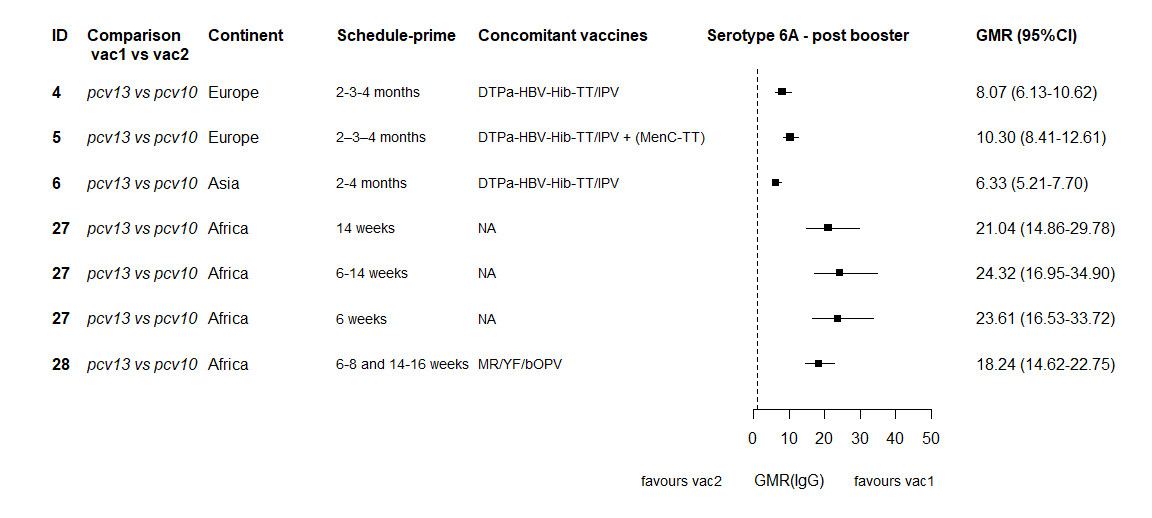


GMR: Geometric mean ratio; pcv: Pneumococcal conjugate vaccine; DTaP – diphtheria and tetanus toxoids, and acellular pertussis vaccine; DTwP – diphtheria and tetanus toxoids, and whole-cell pertussis vaccine; Hib-TT – Haemophilus influenzae type b vaccine (tetanus toxoid conjugate); HB – Hepatitis B vaccine; IPV – Inactivated polio vaccine; OPV – Oral polio vaccine; MenC – Meningococcal C vaccine; TT – tetanus toxoid conjugate; NA: not applicable; enr 3-6 m, 4-8 w int: enrolment at 3-6 months of age, and at 4-8 weeks interval of a 3 doses primary vaccines in total. MR: Measles and rubella combined vaccine; YF: Yellow fever vaccine; bOPV: Bivalent oral poliovirus vaccine

Each solid line in the figure shows the GMR from each trial. Black boxes and lines show the point estimates and confidence intervals for geometric mean ratios comparing vac1 vs vac2. Concomitant vaccines are vaccines co-administered with PCV primary vaccine series. Information on co-administered vaccine is not always available. Concomitant vaccines in the bracket are those administered in some but not all of the study sites.

Supplementary Figure 42. Trial level geometric mean ratios for serotype 19A post-booster.


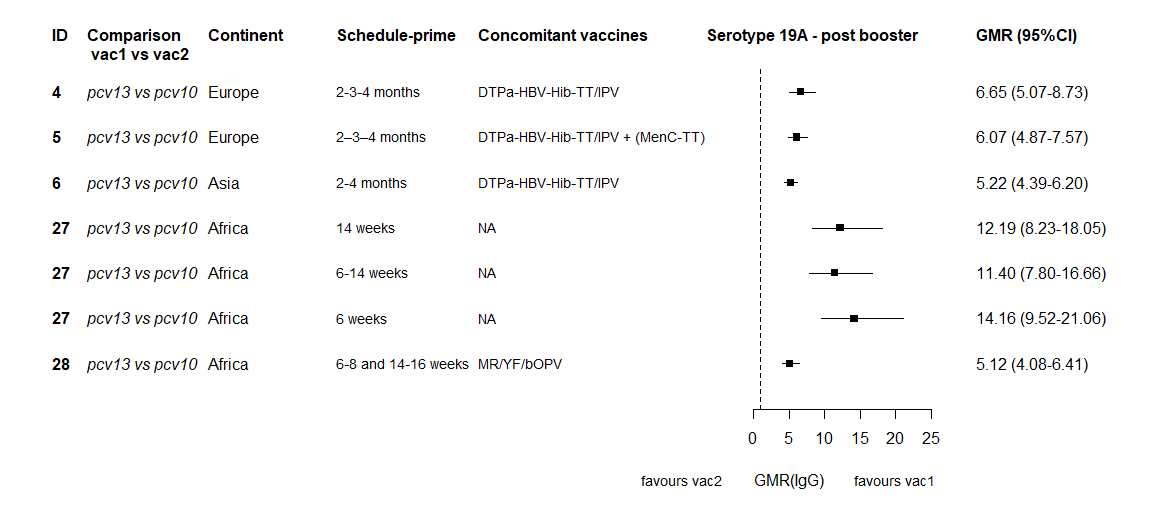


GMR: Geometric mean ratio; pcv: Pneumococcal conjugate vaccine; DTaP – diphtheria and tetanus toxoids, and acellular pertussis vaccine; DTwP – diphtheria and tetanus toxoids, and whole-cell pertussis vaccine; Hib-TT – Haemophilus influenzae type b vaccine (tetanus toxoid conjugate); HB – Hepatitis B vaccine; IPV – Inactivated polio vaccine; OPV – Oral polio vaccine; MenC – Meningococcal C vaccine; TT – tetanus toxoid conjugate; NA: not applicable; enr 3-6 m, 4-8 w int: enrolment at 3-6 months of age, and at 4-8 weeks interval of a 3 doses primary vaccines in total. MR: Measles and rubella combined vaccine; YF: Yellow fever vaccine; bOPV: Bivalent oral poliovirus vaccine

Each solid line in the figure shows the GMR from each trial. Black boxes and lines show the point estimates and confidence intervals for geometric mean ratios comparing vac1 vs vac2. Concomitant vaccines are vaccines co-administered with PCV primary vaccine series. Information on co-administered vaccine is not always available. Concomitant vaccines in the bracket are those administered in some but not all of the study sites.

Supplementary Figure 43. Geometric mean ratios from sensitivity analyses restricted to studies providing data for all three time points a) post-primary vaccination series, b) pre-boost, and c) post-boost

a)
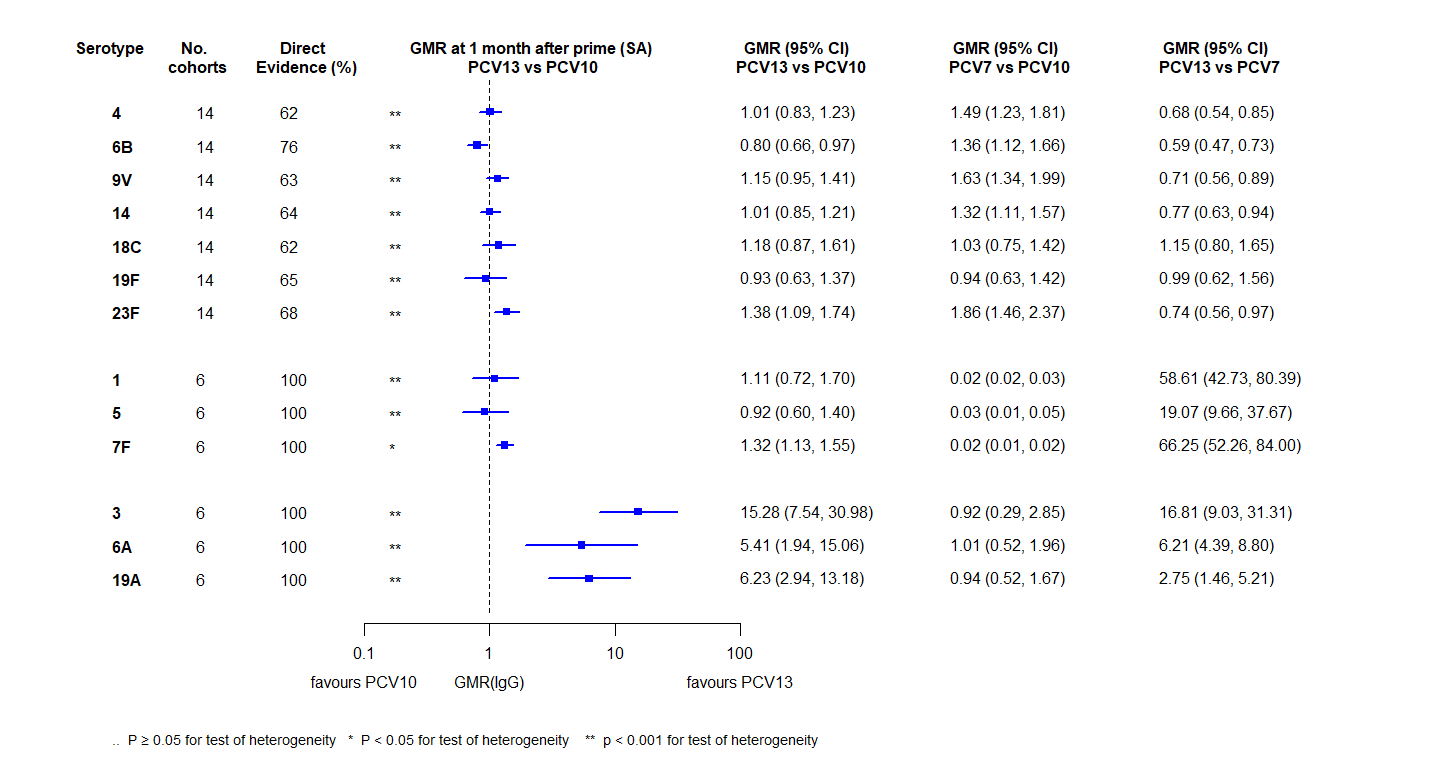


b)
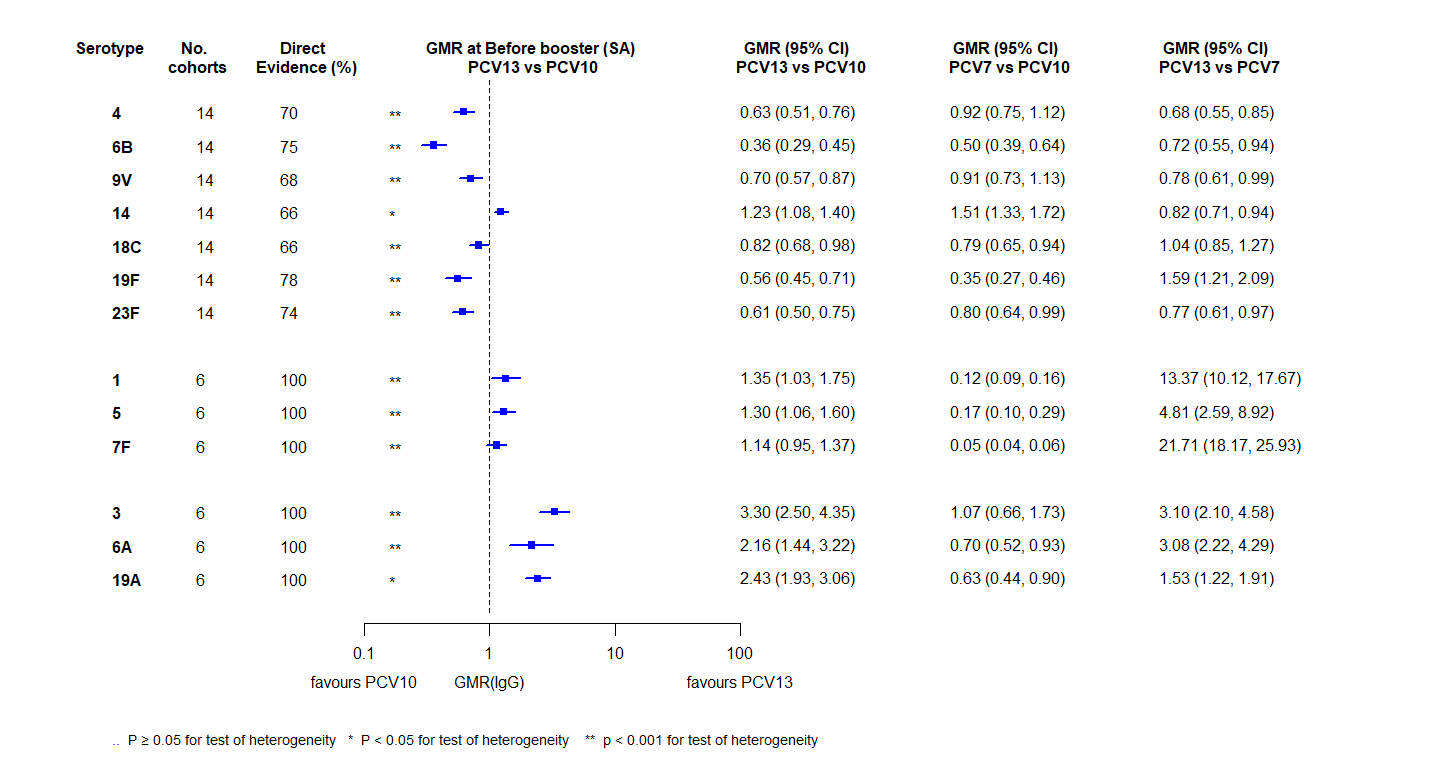


c)
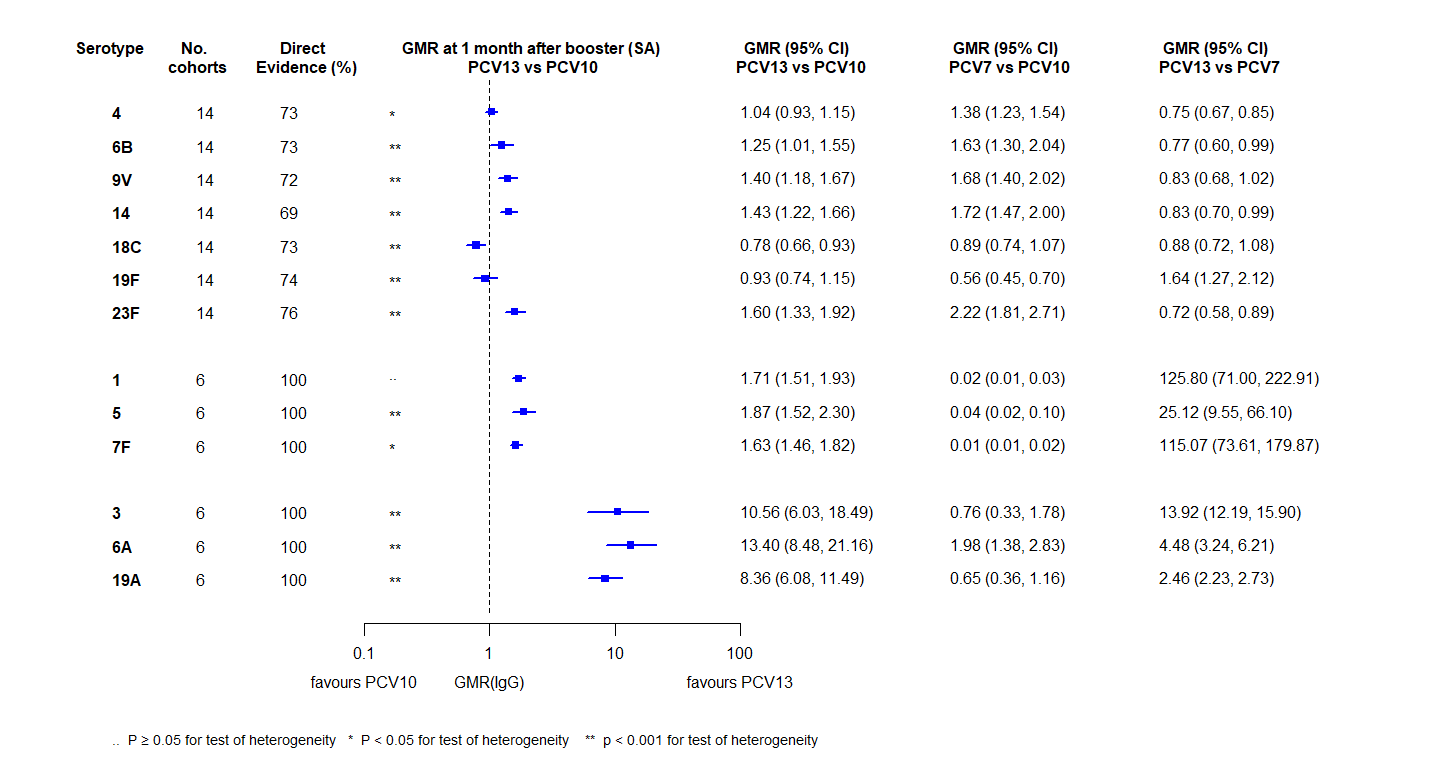


GMR: Geometric mean ratio; PCV: Pneumococcal conjugate vaccine. Each line in the figure shows the output from a network meta-analyses (PCV7 serotypes) or direct meta-analyses (PCV13 but non-PCV7 serotypes). Blue boxes and blue lines show the point estimates and confidence intervals for geometric mean ratios comparing PCV13 vs PCV10. Points to the right of the vertical line are those with higher antibody responses in the PCV13 arm of the study, and points to the left are those with higher antibody responses in the PCV10 arm. The direct evidence column shows the percentage of evidence from studies directly comparing PCV13 vs PCV10 that contributes to the estimates presented in the figure in blue (PCV13 vs PCV10). GMR of PCV13 vs PCV10 for PCV10 and PCV13 serotypes are from a meta-analysis of only head-to-head studies of PCV13 vs PCV10. SA: sensitivity analysis

Supplementary Figure 44. Geometric mean ratios from sensitivity analysis of studies conducted in Europe at a) post-primary vaccination series, b) pre-boost, and c) post-boost

a)
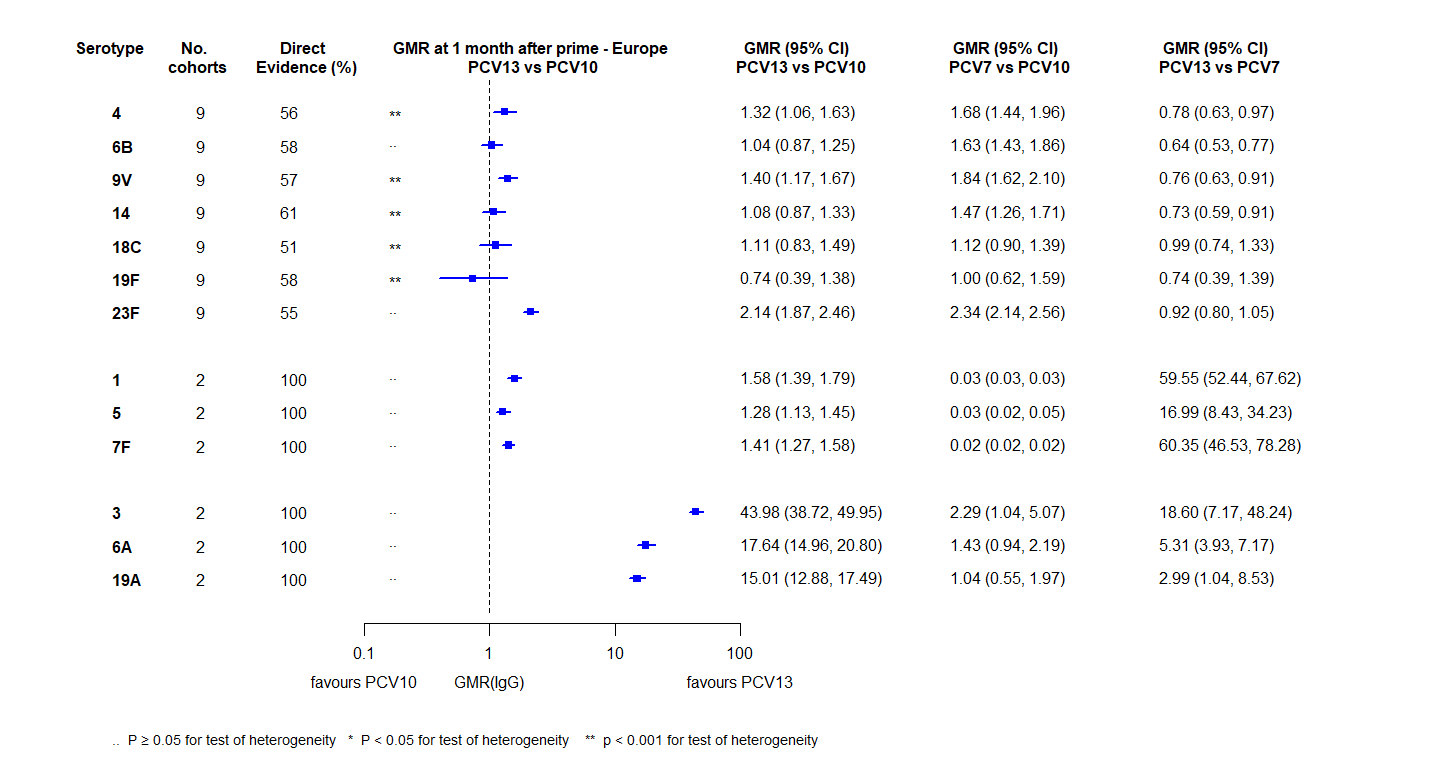


b)
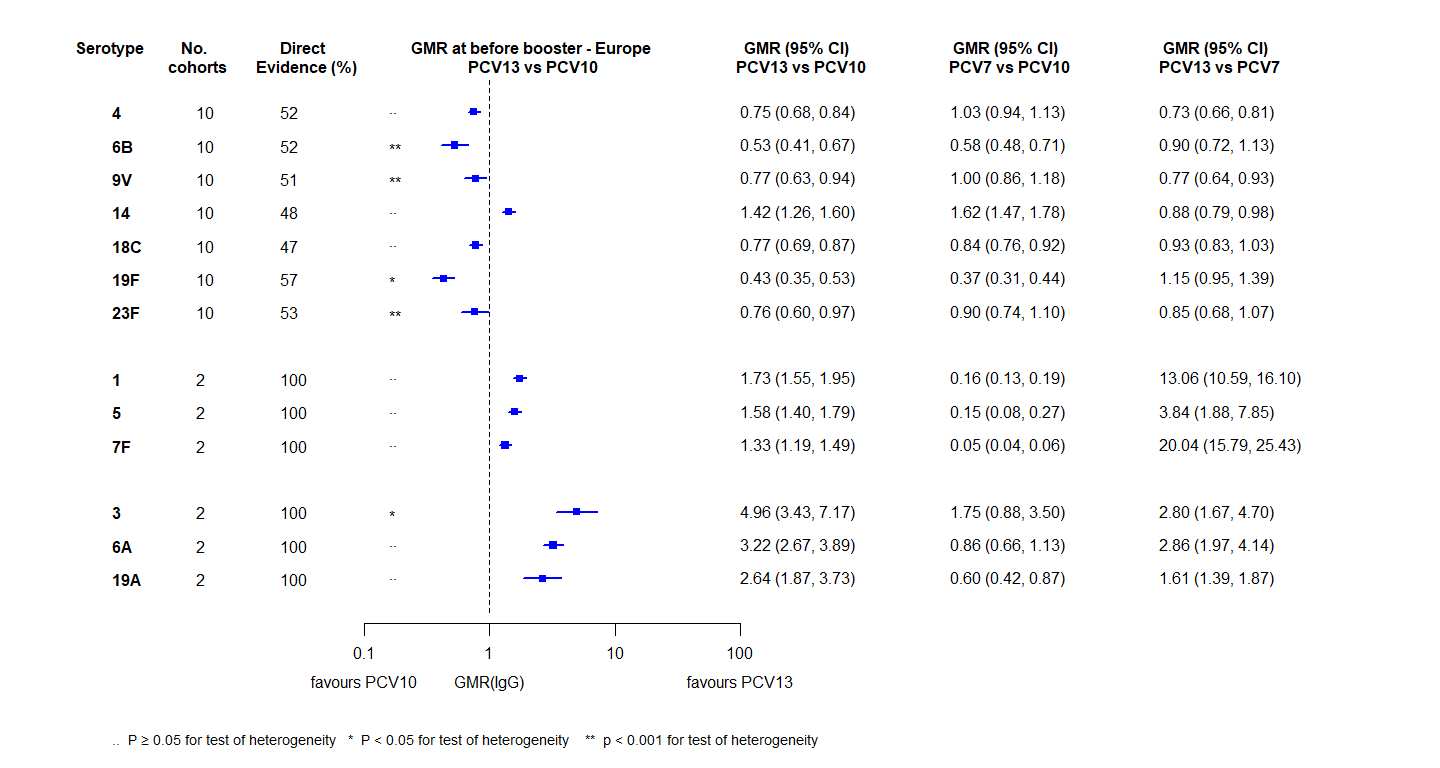


c)
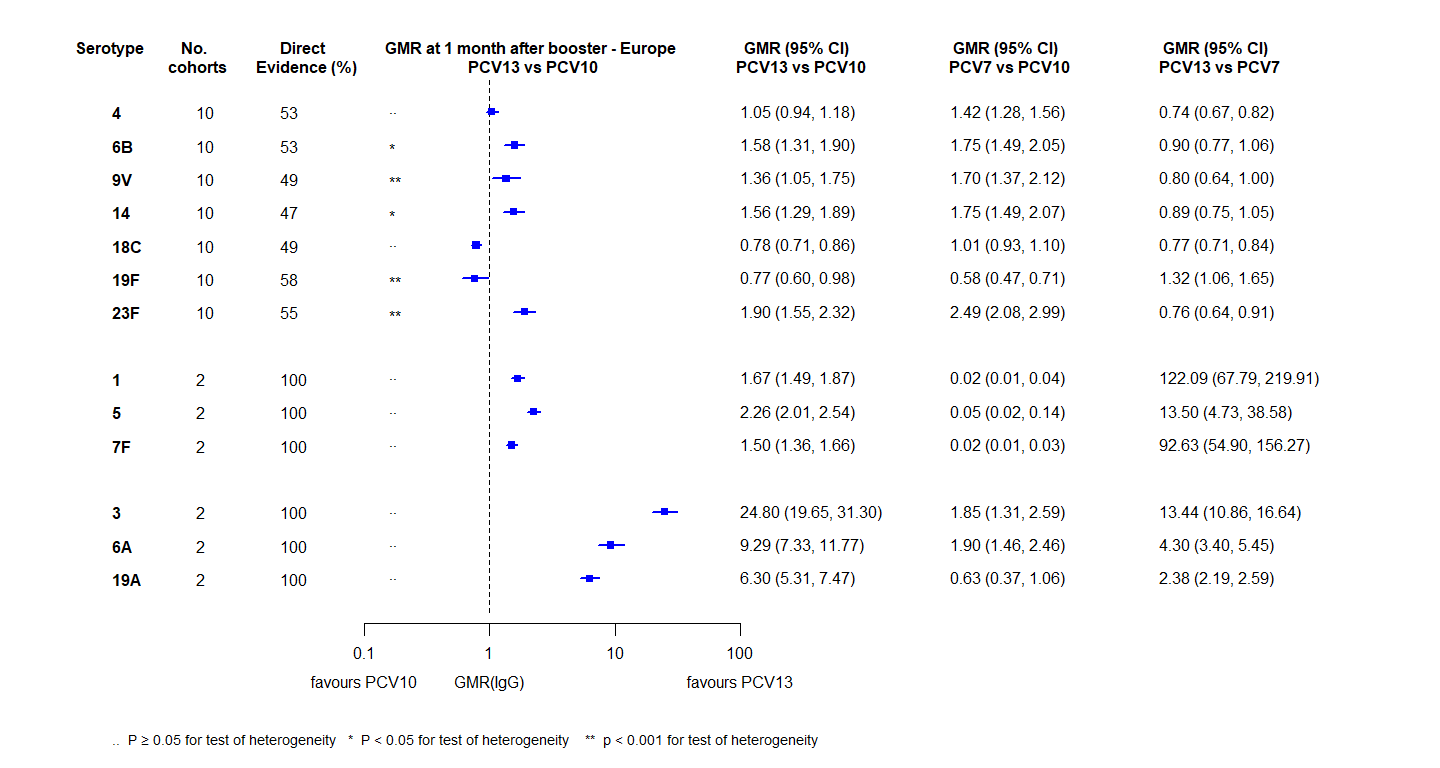


GMR: Geometric mean ratio; PCV: Pneumococcal conjugate vaccine. Each line in the figure shows the output from a network meta-analyses (PCV7 serotypes) or direct meta-analyses (PCV13 but non-PCV7 serotypes). Blue boxes and blue lines show the point estimates and confidence intervals for geometric mean ratios comparing PCV13 vs PCV10. Points to the right of the vertical line are those with higher antibody responses in the PCV13 arm of the study, and points to the left are those with higher antibody responses in the PCV10 arm. The direct evidence column shows the percentage of evidence from studies directly comparing PCV13 vs PCV10 that contributes to the estimates presented in the figure in blue (PCV13 vs PCV10). GMR of PCV13 vs PCV10 for PCV10 and PCV13 serotypes are from a meta-analysis of only head-to-head studies of PCV13 vs PCV10.

Supplementary Figure 45. Geometric mean ratios from sensitivity analyses of studies conducted in Asia: a) post-primary vaccination series and b) post-boost

a)
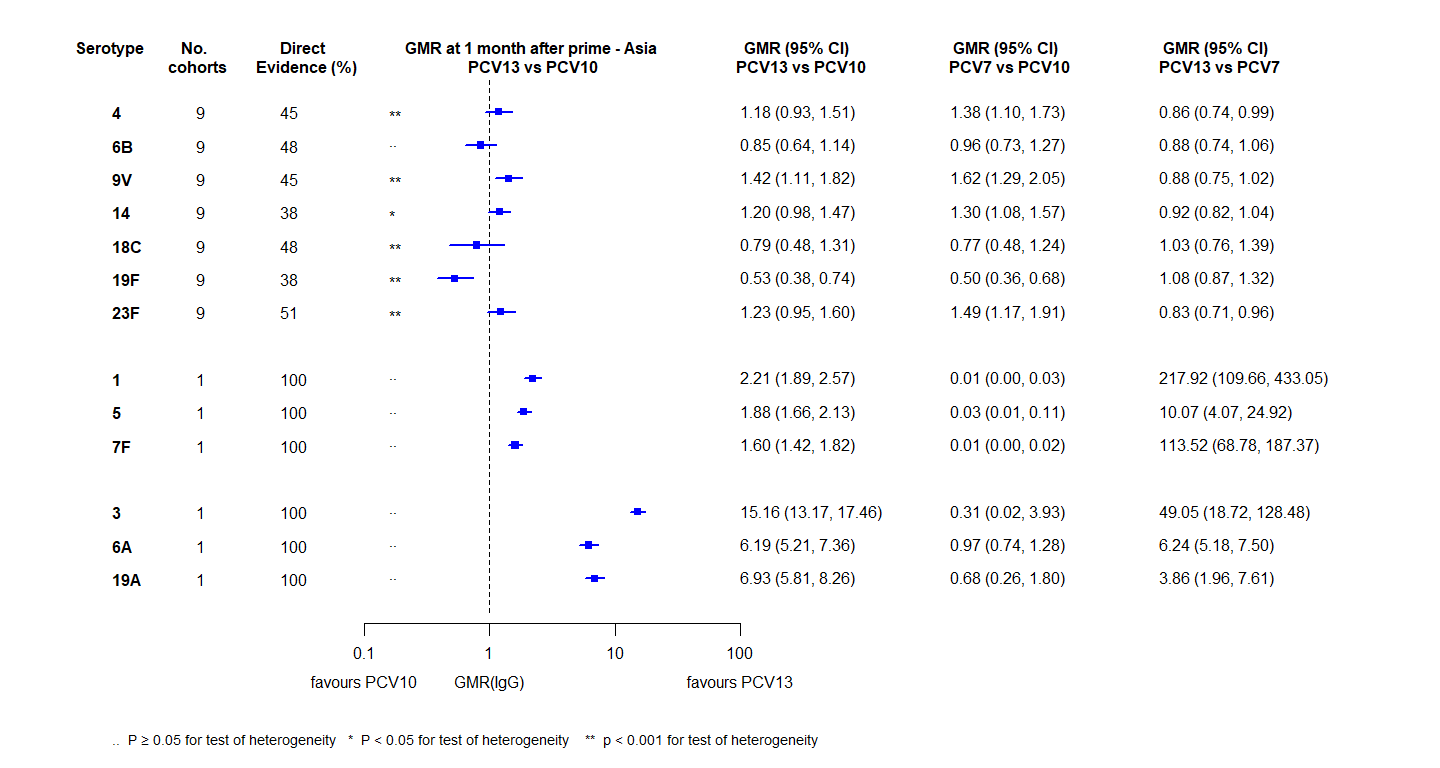


b)
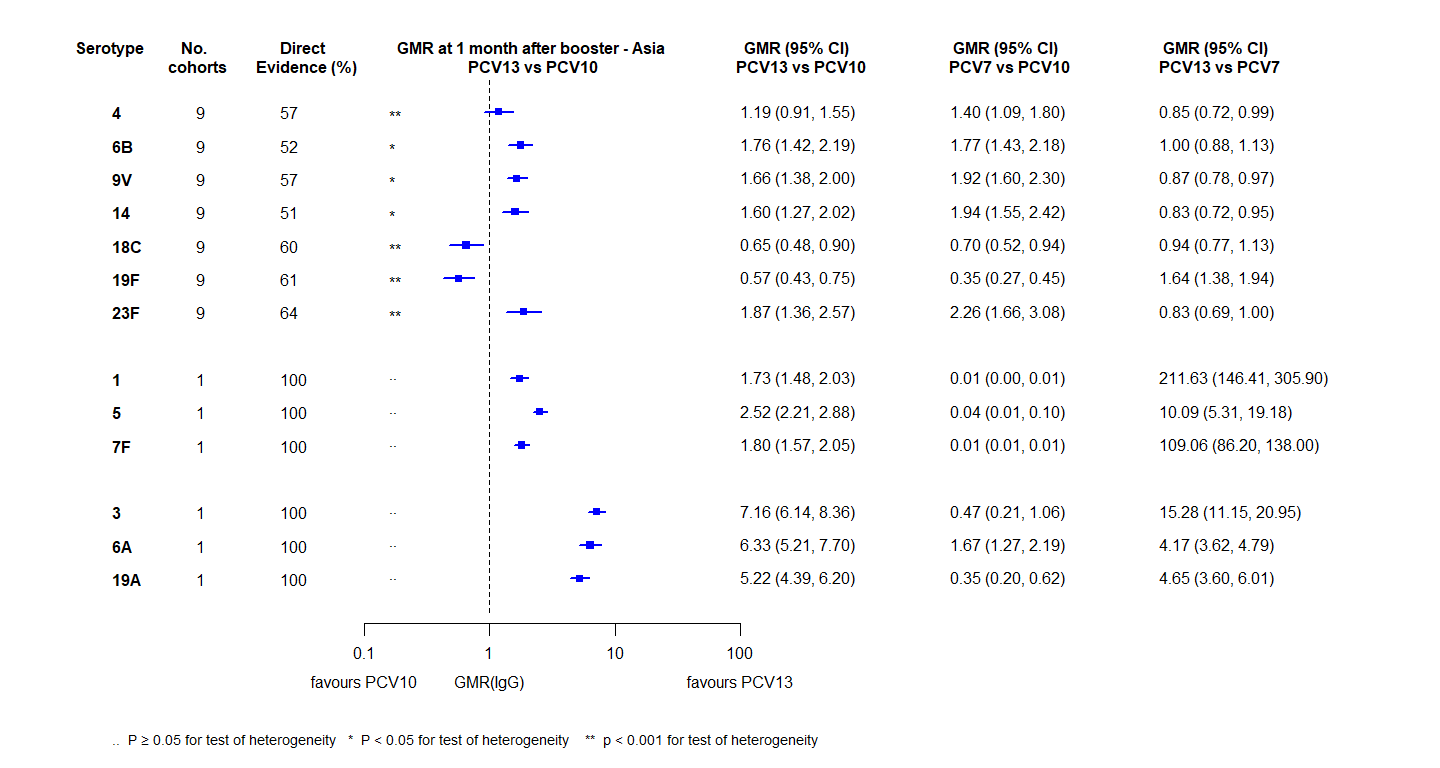


GMR: Geometric mean ratio; PCV: Pneumococcal conjugate vaccine. Each line in the figure shows the output from a network meta-analyses (PCV7 serotypes) or direct meta-analyses (PCV13 but non-PCV7 serotypes). Blue boxes and blue lines show the point estimates and confidence intervals for geometric mean ratios comparing PCV13 vs PCV10. Points to the right of the vertical line are those with higher antibody responses in the PCV13 arm of the study, and points to the left are those with higher antibody responses in the PCV10 arm. The direct evidence column shows the percentage of evidence from studies directly comparing PCV13 vs PCV10 that contributes to the estimates presented in the figure in blue (PCV13 vs PCV10). GMR of PCV13 vs PCV10 for PCV10 and PCV13 serotypes are from a meta-analysis of only head-to-head studies of PCV13 vs PCV10.

Supplementary Figure 46. Geometric mean ratios from sensitivity analyses of studies that used a 3+1 schedule at a) post-primary vaccination series, b) pre-boost, and c) post-boost

a)
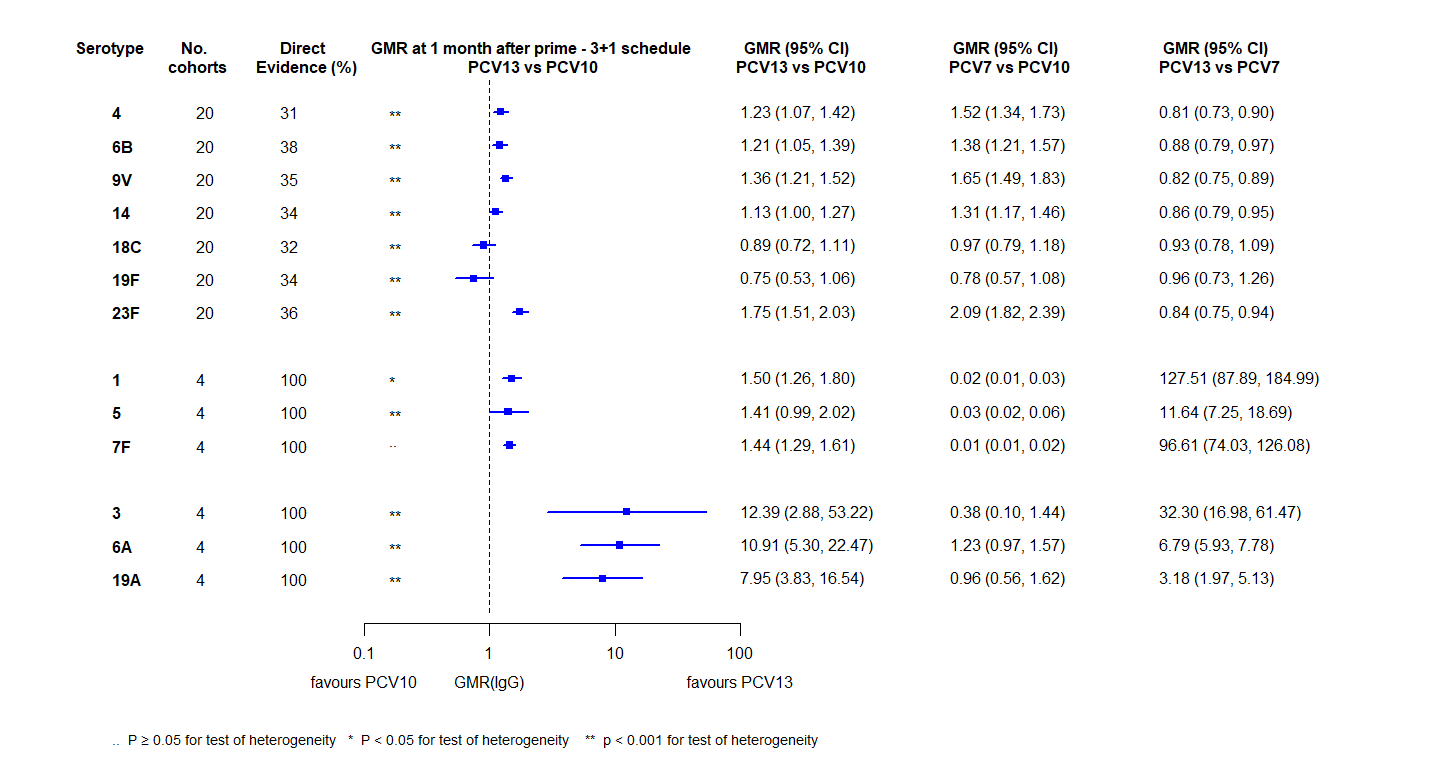


b)
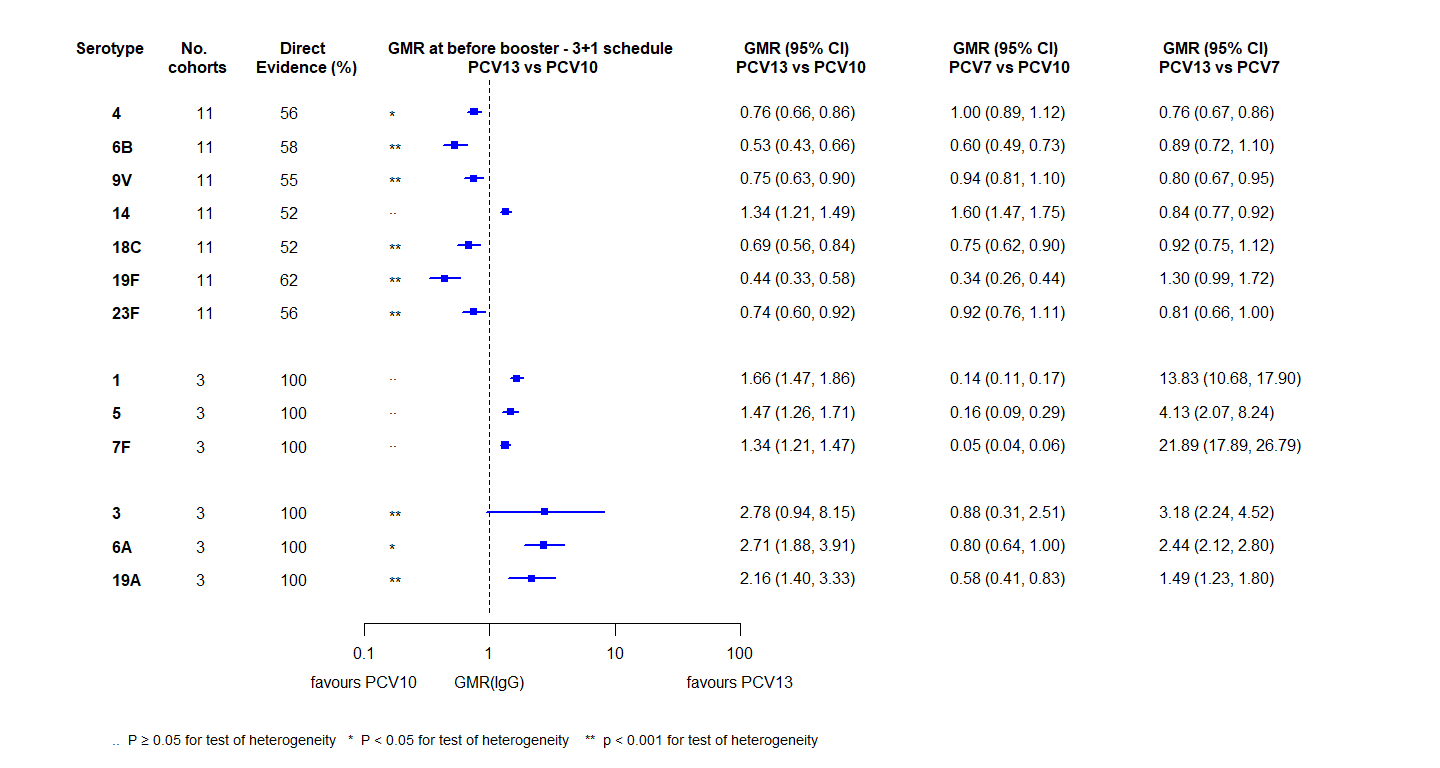


c)
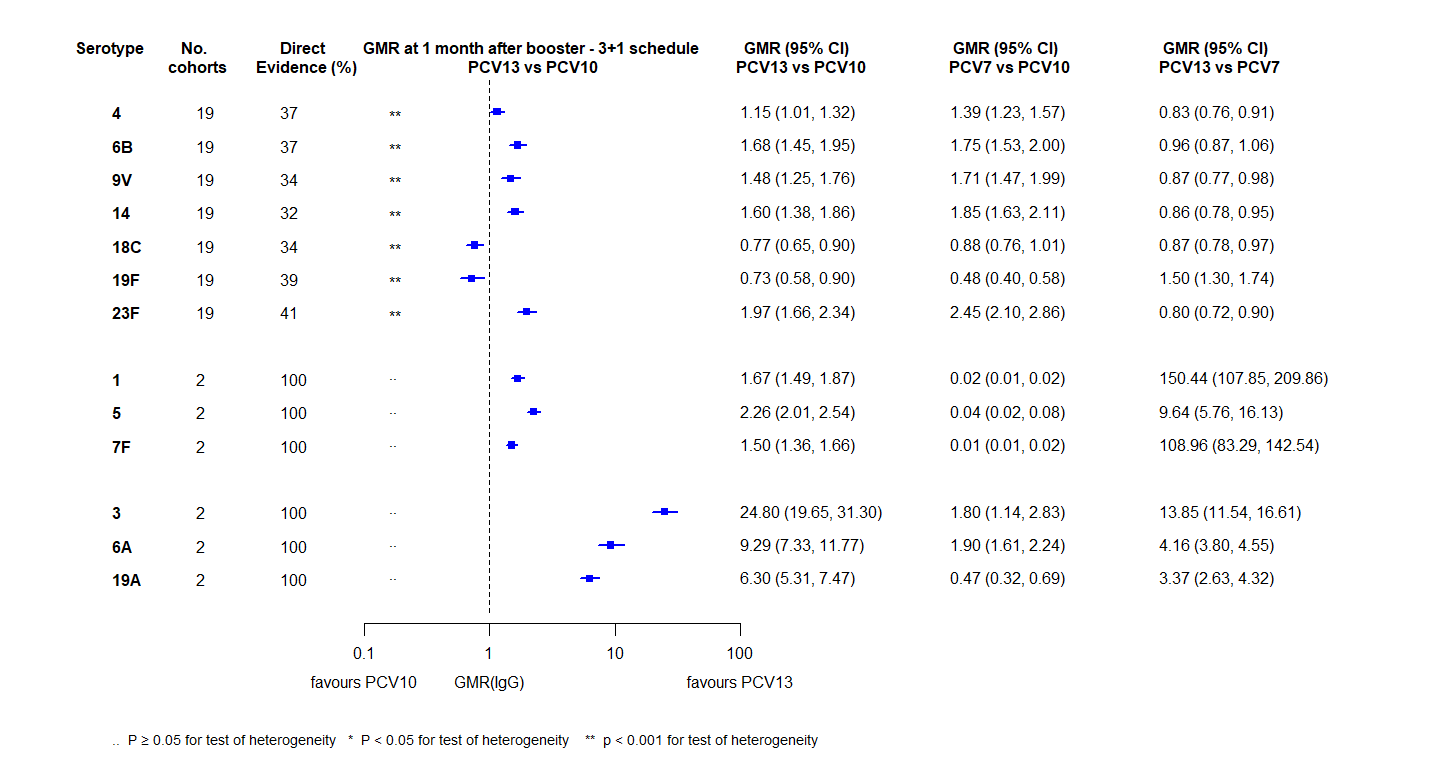


GMR: Geometric mean ratio; PCV: Pneumococcal conjugate vaccine. Each line in the figure shows the output from a network meta-analyses (PCV7 serotypes) or direct meta-analyses (PCV13 but non-PCV7 serotypes). Blue boxes and blue lines show the point estimates and confidence intervals for geometric mean ratios comparing PCV13 vs PCV10. Points to the right of the vertical line are those with higher antibody responses in the PCV13 arm of the study, and points to the left are those with higher antibody responses in the PCV10 arm. The direct evidence column shows the percentage of evidence from studies directly comparing PCV13 vs PCV10 that contributes to the estimates presented in the figure in blue (PCV13 vs PCV10). GMR of PCV13 vs PCV10 for PCV10 and PCV13 serotypes are from a meta-analysis of only head-to-head studies of PCV13 vs PCV10.

Supplementary Figure 47. Direct and indirect evidence on relative risk comparing PCV13 vs PCV10 for serotypes in PCV7 (4, 6B, 9V, 14, 18C, 19F and 23F)


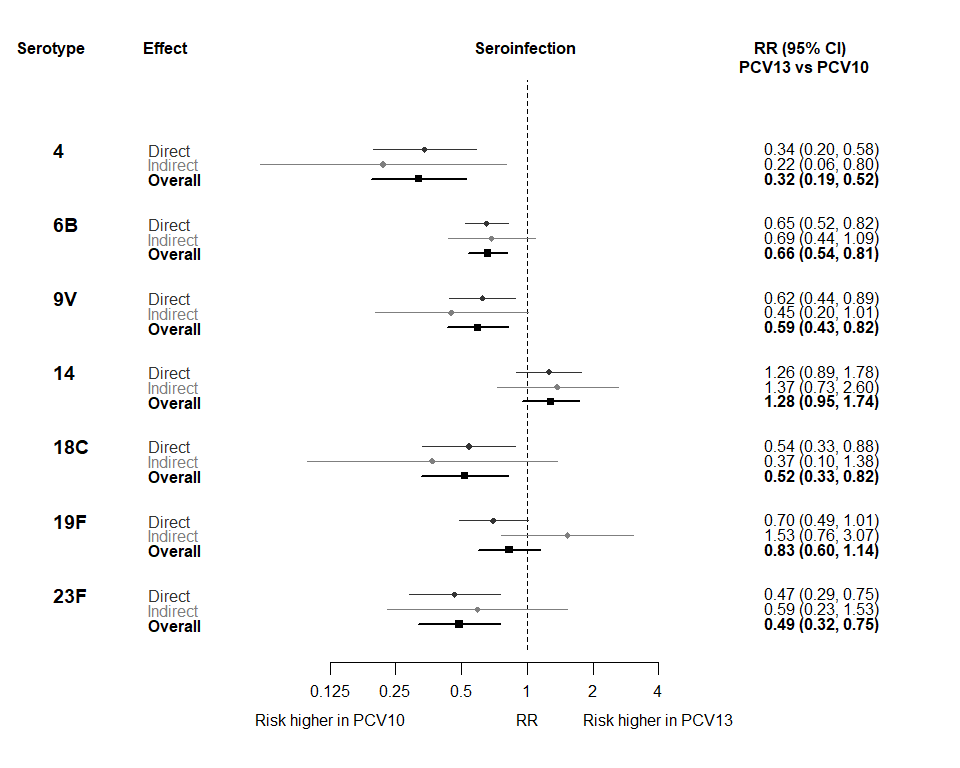


RR: Relative risk; PCV: Pneumococcal conjugate vaccine. Each line in the figure shows the output from a network meta-analyses. Dark grey diamonds and lines show the point estimates and confidence intervals relative risks from studies directly comparing PCV13 vs PCV10. Light grey diamonds and lines show the point estimates and confidence intervals for relative risks from studies comparing PCV13 vs PCV10 through PCV7. Black boxes and lines show the point estimates and confidence intervals incorporating both direct and indirect evidence.

Supplementary Figure 48. Trial level relative risk for serotype 4.


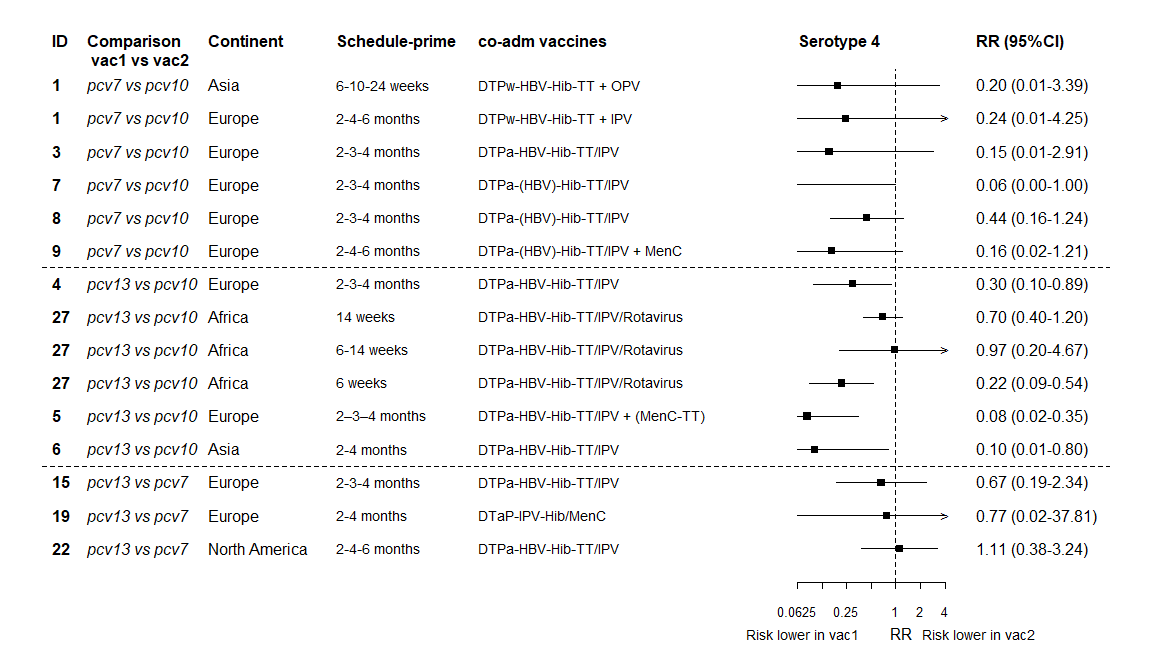


RR: relative risk; pcv: Pneumococcal conjugate vaccine; DTaP – diphtheria and tetanus toxoids, and acellular pertussis vaccine; DTwP – diphtheria and tetanus toxoids, and whole-cell pertussis vaccine; Hib-TT – Haemophilus influenzae type b vaccine (tetanus toxoid conjugate); HB – Hepatitis B vaccine; IPV – Inactivated polio vaccine; OPV – Oral polio vaccine; MenC – Meningococcal C vaccine; TT – tetanus toxoid conjugate; NA: not applicable.

Each solid line in the figure shows the RR from each trial. Black boxes and lines show the point estimates and confidence intervals for relative risks comparing vac1 vs vac2. co-adm vaccines are vaccines co-administered with PCV primary vaccine series. Information on co-administered vaccine is not always available. Concomitant vaccines in the bracket are those administered in some but not all of the study sites.

Supplementary Figure 49. Trial level relative risk for serotype 6B.


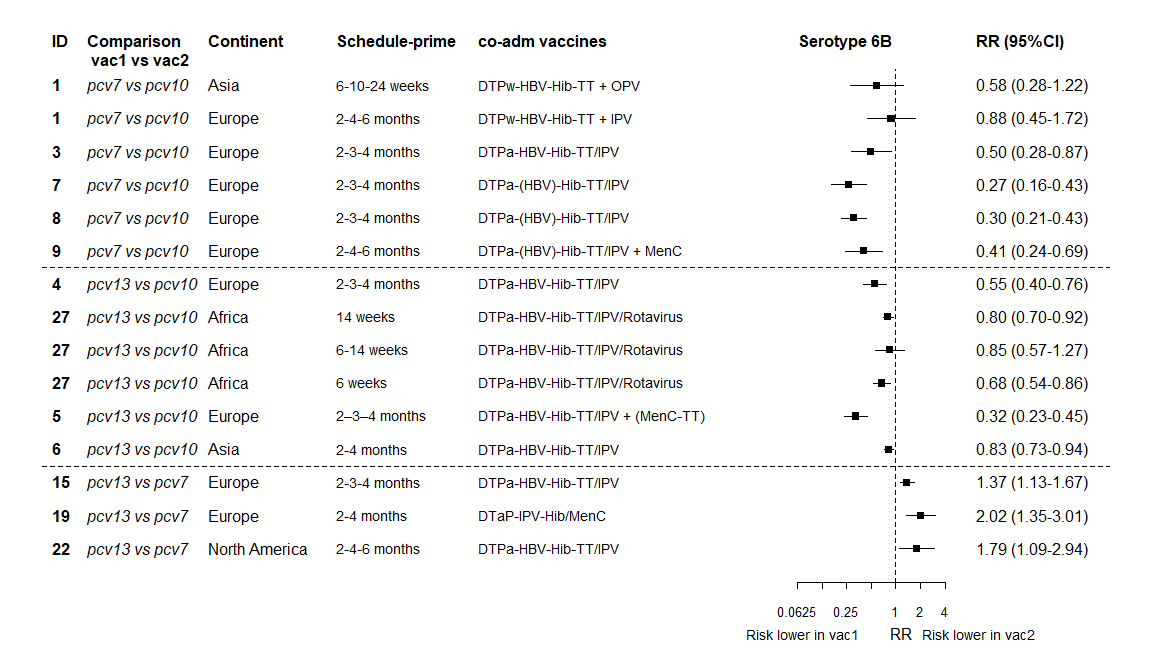


RR: relative risk; pcv: Pneumococcal conjugate vaccine; DTaP – diphtheria and tetanus toxoids, and acellular pertussis vaccine; DTwP – diphtheria and tetanus toxoids, and whole-cell pertussis vaccine; Hib-TT – Haemophilus influenzae type b vaccine (tetanus toxoid conjugate); HB – Hepatitis B vaccine; IPV – Inactivated polio vaccine; OPV – Oral polio vaccine; MenC – Meningococcal C vaccine; TT – tetanus toxoid conjugate; NA: not applicable.

Each solid line in the figure shows the RR from each trial. Black boxes and lines show the point estimates and confidence intervals for relative risks comparing vac1 vs vac2. co-adm vaccines are vaccines co-administered with PCV primary vaccine series. Information on co-administered vaccine is not always available. Concomitant vaccines in the bracket are those administered in some but not all of the study sites.

Supplementary Figure 50. Trial level relative risk for serotype 9V.


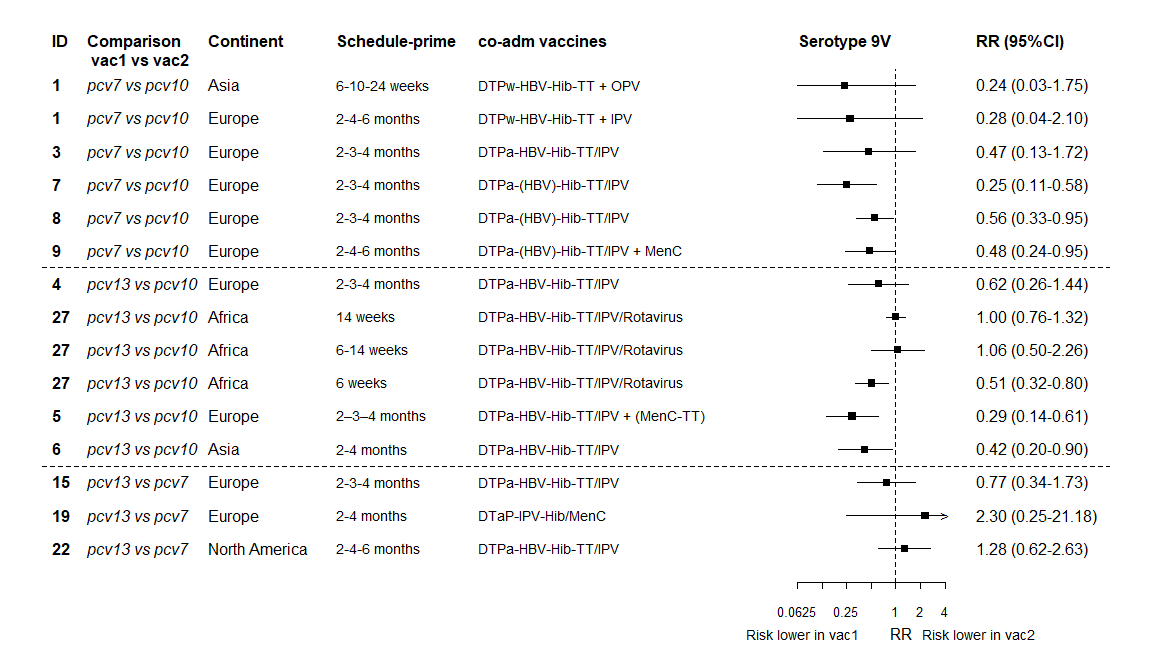


RR: relative risk; pcv: Pneumococcal conjugate vaccine; DTaP – diphtheria and tetanus toxoids, and acellular pertussis vaccine; DTwP – diphtheria and tetanus toxoids, and whole-cell pertussis vaccine; Hib-TT – Haemophilus influenzae type b vaccine (tetanus toxoid conjugate); HB – Hepatitis B vaccine; IPV – Inactivated polio vaccine; OPV – Oral polio vaccine; MenC – Meningococcal C vaccine; TT – tetanus toxoid conjugate; NA: not applicable.

Each solid line in the figure shows the RR from each trial. Black boxes and lines show the point estimates and confidence intervals for relative risks comparing vac1 vs vac2. co-adm vaccines are vaccines co-administered with PCV primary vaccine series. Information on co-administered vaccine is not always available. Concomitant vaccines in the bracket are those administered in some but not all of the study sites.

Supplementary Figure 51. Trial level relative risk for serotype 14.


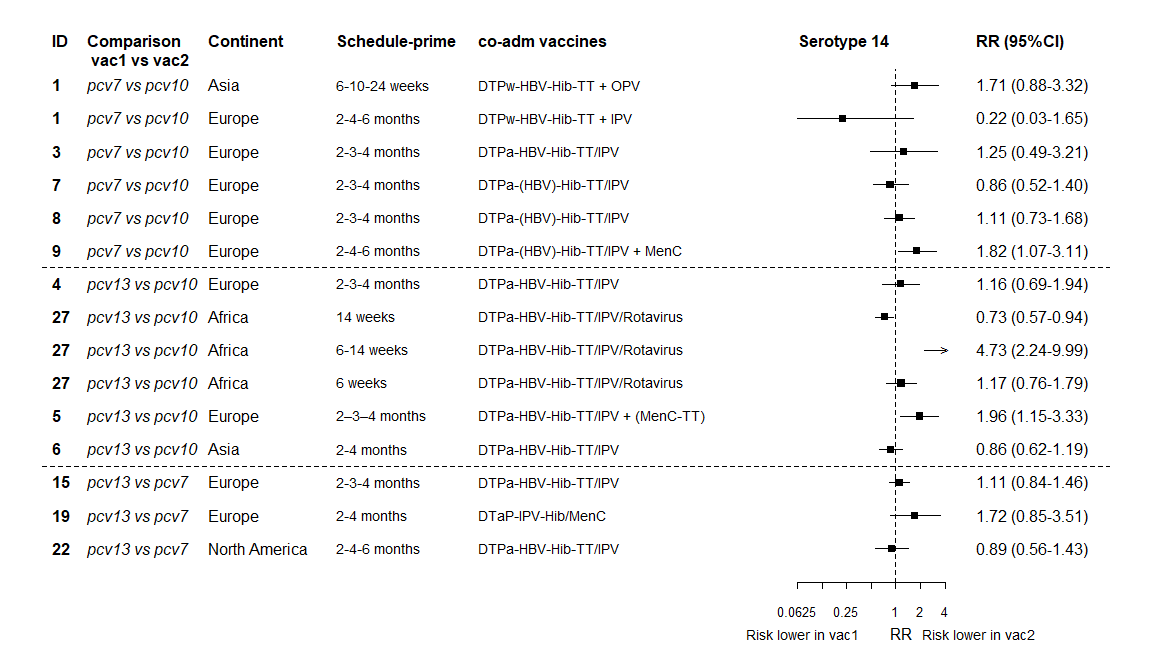


RR: relative risk; pcv: Pneumococcal conjugate vaccine; DTaP – diphtheria and tetanus toxoids, and acellular pertussis vaccine; DTwP – diphtheria and tetanus toxoids, and whole-cell pertussis vaccine; Hib-TT – Haemophilus influenzae type b vaccine (tetanus toxoid conjugate); HB – Hepatitis B vaccine; IPV – Inactivated polio vaccine; OPV – Oral polio vaccine; MenC – Meningococcal C vaccine; TT – tetanus toxoid conjugate; NA: not applicable.

Each solid line in the figure shows the RR from each trial. Black boxes and lines show the point estimates and confidence intervals for relative risks comparing vac1 vs vac2. co-adm vaccines are vaccines co-administered with PCV primary vaccine series. Information on co-administered vaccine is not always available. Concomitant vaccines in the bracket are those administered in some but not all of the study sites.

Supplementary Figure 52. Trial level relative risk for serotype 18C.


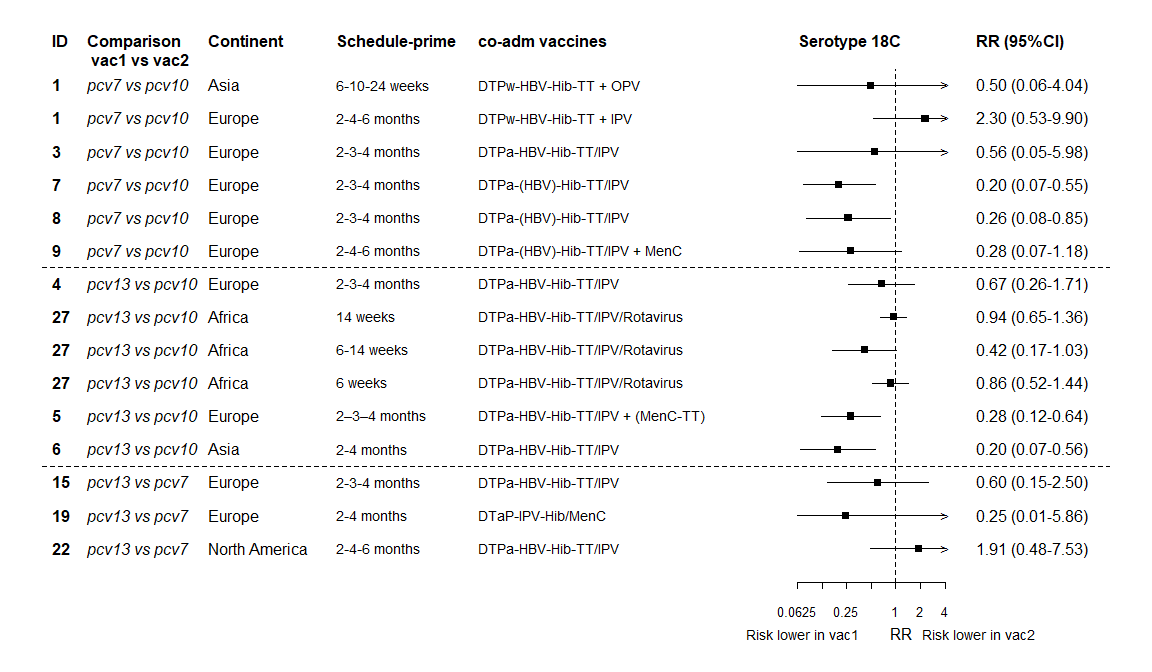


RR: relative risk; pcv: Pneumococcal conjugate vaccine; DTaP – diphtheria and tetanus toxoids, and acellular pertussis vaccine; DTwP – diphtheria and tetanus toxoids, and whole-cell pertussis vaccine; Hib-TT – Haemophilus influenzae type b vaccine (tetanus toxoid conjugate); HB – Hepatitis B vaccine; IPV – Inactivated polio vaccine; OPV – Oral polio vaccine; MenC – Meningococcal C vaccine; TT – tetanus toxoid conjugate; NA: not applicable.

Each solid line in the figure shows the RR from each trial. Black boxes and lines show the point estimates and confidence intervals for relative risks comparing vac1 vs vac2. co-adm vaccines are vaccines co-administered with PCV primary vaccine series. Information on co-administered vaccine is not always available. Concomitant vaccines in the bracket are those administered in some but not all of the study sites.

Supplementary Figure 53. Trial level relative risk for serotype 4.


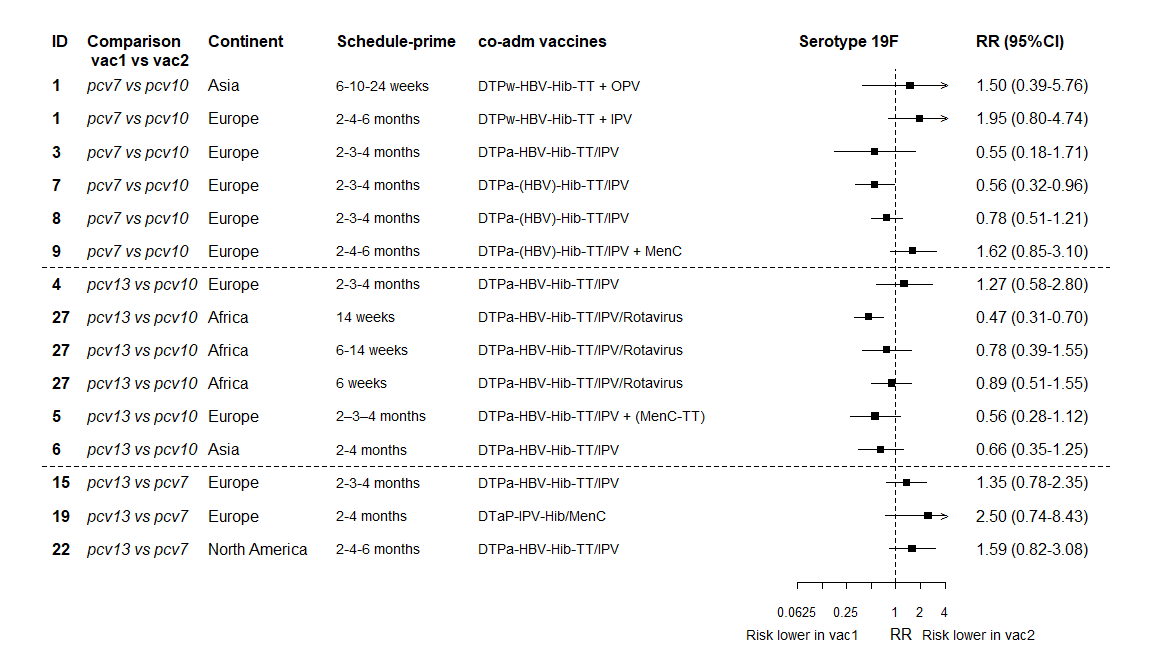


RR: relative risk; pcv: Pneumococcal conjugate vaccine; DTaP – diphtheria and tetanus toxoids, and acellular pertussis vaccine; DTwP – diphtheria and tetanus toxoids, and whole-cell pertussis vaccine; Hib-TT – Haemophilus influenzae type b vaccine (tetanus toxoid conjugate); HB – Hepatitis B vaccine; IPV – Inactivated polio vaccine; OPV – Oral polio vaccine; MenC – Meningococcal C vaccine; TT – tetanus toxoid conjugate; NA: not applicable.

Each solid line in the figure shows the RR from each trial. Black boxes and lines show the point estimates and confidence intervals for relative risks comparing vac1 vs vac2. co-adm vaccines are vaccines co-administered with PCV primary vaccine series. Information on co-administered vaccine is not always available. Concomitant vaccines in the bracket are those administered in some but not all of the study sites.

Supplementary Figure 54. Trial level relative risk for serotype 23F.


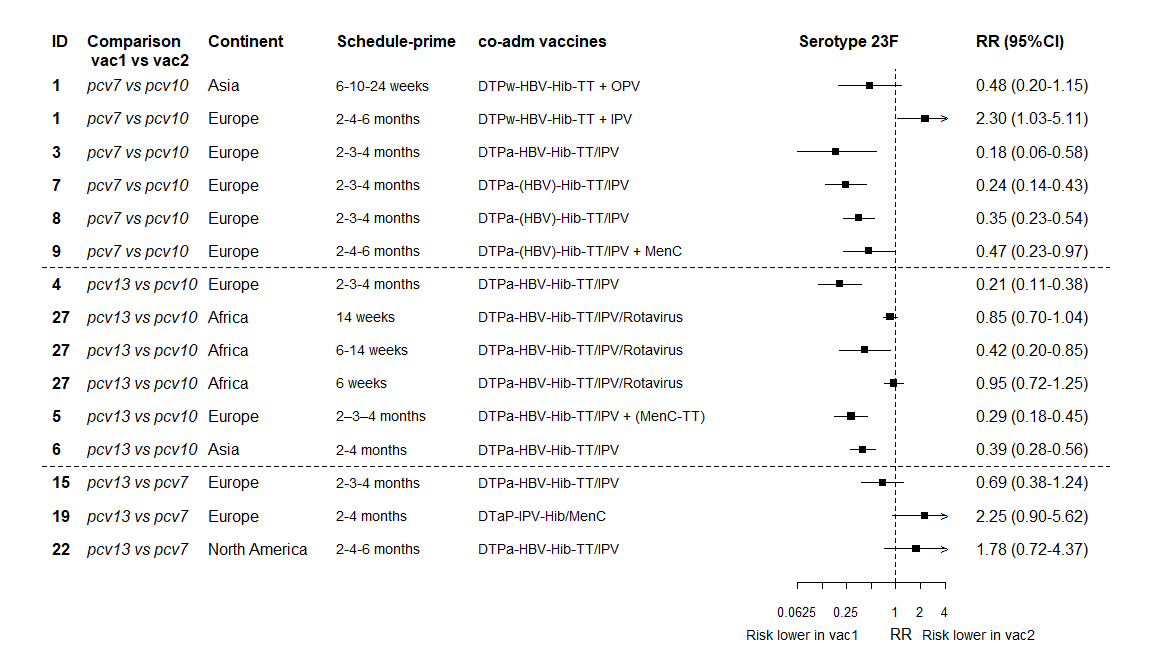


RR: relative risk; pcv: Pneumococcal conjugate vaccine; DTaP – diphtheria and tetanus toxoids, and acellular pertussis vaccine; DTwP – diphtheria and tetanus toxoids, and whole-cell pertussis vaccine; Hib-TT – Haemophilus influenzae type b vaccine (tetanus toxoid conjugate); HB – Hepatitis B vaccine; IPV – Inactivated polio vaccine; OPV – Oral polio vaccine; MenC – Meningococcal C vaccine; TT – tetanus toxoid conjugate; NA: not applicable.

Each solid line in the figure shows the RR from each trial. Black boxes and lines show the point estimates and confidence intervals for relative risks comparing vac1 vs vac2. co-adm vaccines are vaccines co-administered with PCV primary vaccine series. Information on co-administered vaccine is not always available. Concomitant vaccines in the bracket are those administered in some but not all of the study sites.

Supplementary Figure 55. Trial level relative risk for serotype 1.


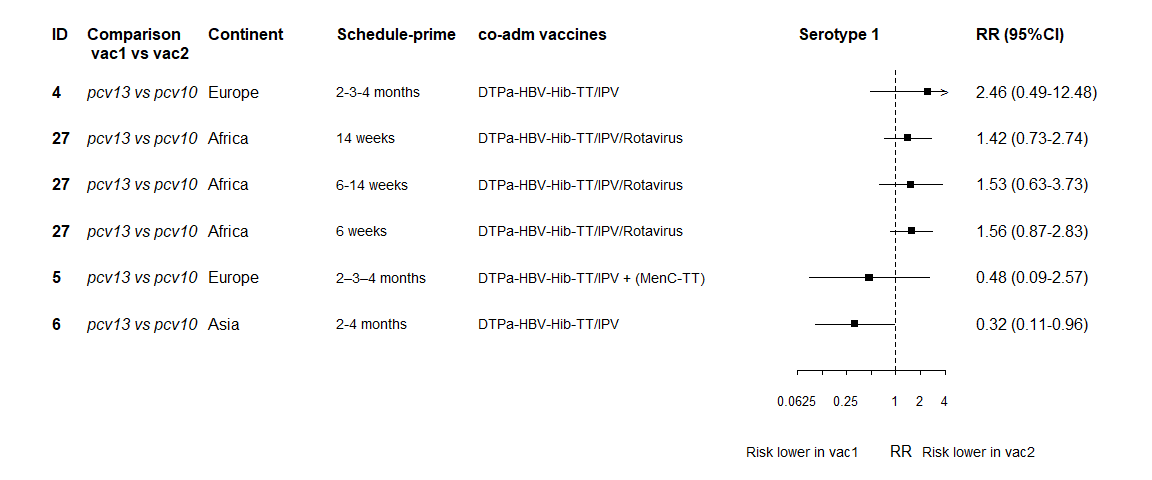


RR: relative risk; pcv: Pneumococcal conjugate vaccine; DTaP – diphtheria and tetanus toxoids, and acellular pertussis vaccine; DTwP – diphtheria and tetanus toxoids, and whole-cell pertussis vaccine; Hib-TT – Haemophilus influenzae type b vaccine (tetanus toxoid conjugate); HB – Hepatitis B vaccine; IPV – Inactivated polio vaccine; OPV – Oral polio vaccine; MenC – Meningococcal C vaccine; TT – tetanus toxoid conjugate; NA: not applicable.

Each solid line in the figure shows the RR from each trial. Black boxes and lines show the point estimates and confidence intervals for relative risks comparing vac1 vs vac2. co-adm vaccines are vaccines co-administered with PCV primary vaccine series. Information on co-administered vaccine is not always available. Concomitant vaccines in the bracket are those administered in some but not all of the study sites.

Supplementary Figure 56. Trial level relative risk for serotype 5.


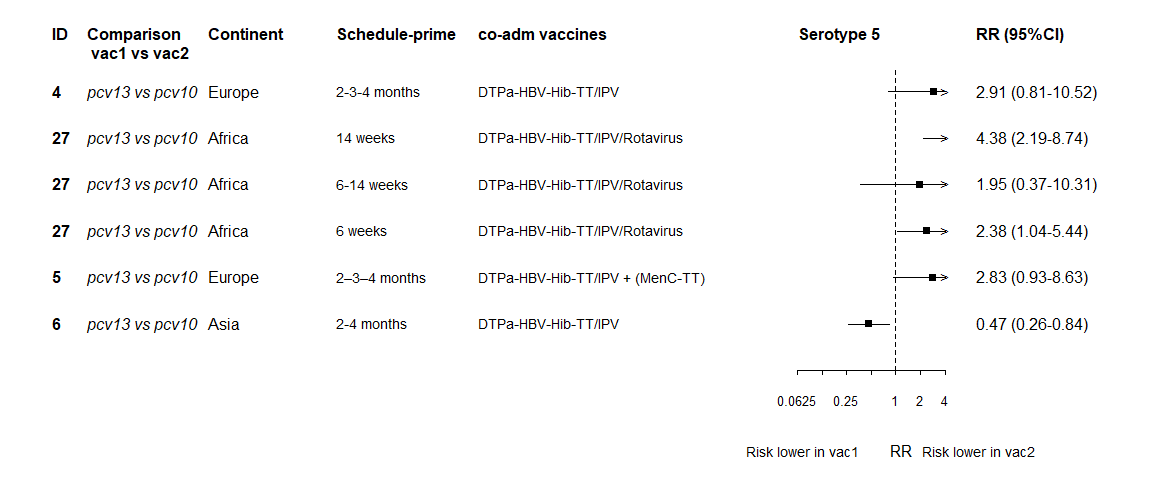


RR: relative risk; pcv: Pneumococcal conjugate vaccine; DTaP – diphtheria and tetanus toxoids, and acellular pertussis vaccine; DTwP – diphtheria and tetanus toxoids, and whole-cell pertussis vaccine; Hib-TT – Haemophilus influenzae type b vaccine (tetanus toxoid conjugate); HB – Hepatitis B vaccine; IPV – Inactivated polio vaccine; OPV – Oral polio vaccine; MenC – Meningococcal C vaccine; TT – tetanus toxoid conjugate; NA: not applicable.

Each solid line in the figure shows the RR from each trial. Black boxes and lines show the point estimates and confidence intervals for relative risks comparing vac1 vs vac2. co-adm vaccines are vaccines co-administered with PCV primary vaccine series. Information on co-administered vaccine is not always available. Concomitant vaccines in the bracket are those administered in some but not all of the study sites.

Supplementary Figure 57. Trial level relative risk for serotype 7F.


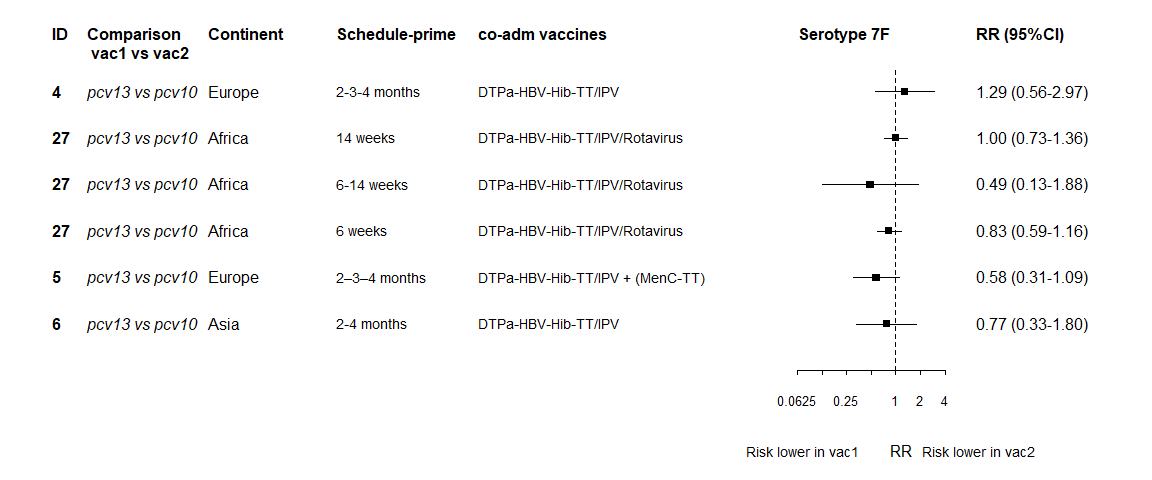


RR: relative risk; pcv: Pneumococcal conjugate vaccine; DTaP – diphtheria and tetanus toxoids, and acellular pertussis vaccine; DTwP – diphtheria and tetanus toxoids, and whole-cell pertussis vaccine; Hib-TT – Haemophilus influenzae type b vaccine (tetanus toxoid conjugate); HB – Hepatitis B vaccine; IPV – Inactivated polio vaccine; OPV – Oral polio vaccine; MenC – Meningococcal C vaccine; TT – tetanus toxoid conjugate; NA: not applicable.

Each solid line in the figure shows the RR from each trial. Black boxes and lines show the point estimates and confidence intervals for relative risks comparing vac1 vs vac2. co-adm vaccines are vaccines co-administered with PCV primary vaccine series. Information on co-administered vaccine is not always available. Concomitant vaccines in the bracket are those administered in some but not all of the study sites.

Supplementary Figure 58. Sensitivity analysis on relative risk of seroinfection for studies conducted in Europe.


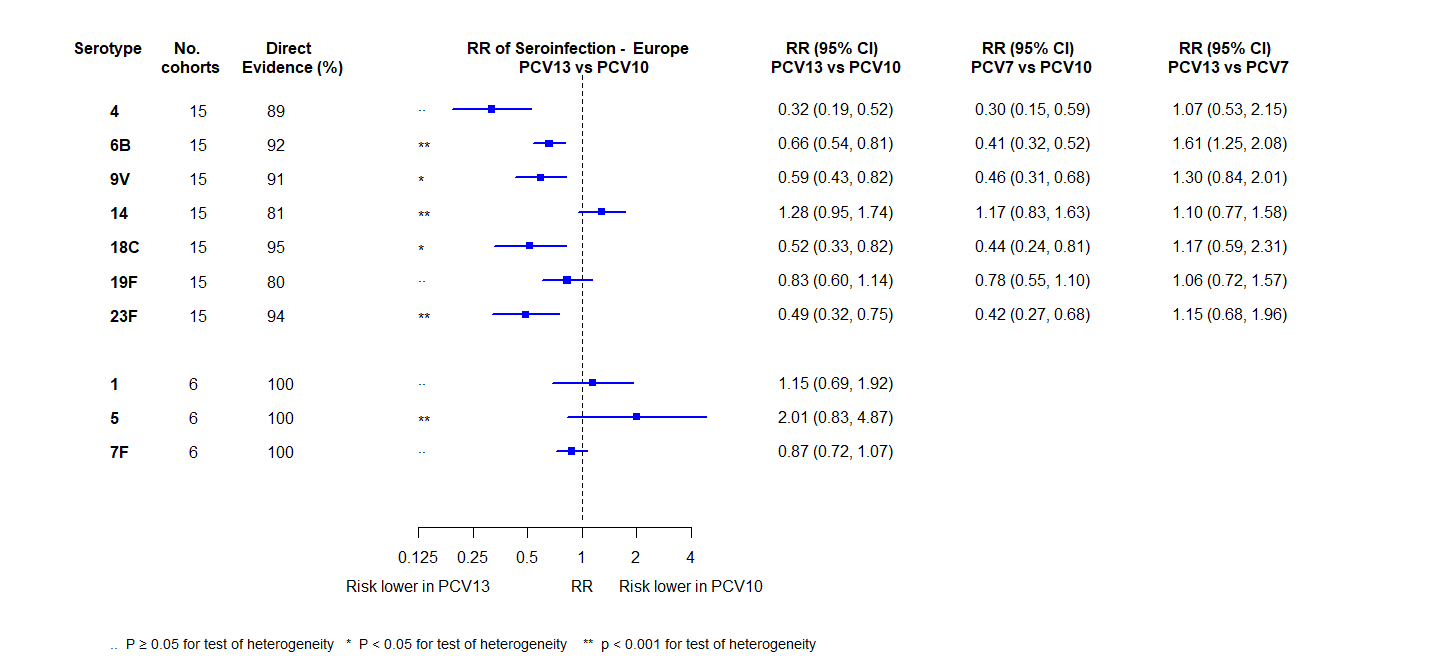


RR: relative risk; PCV: Pneumococcal conjugate vaccine. Each line in the figure shows the output from a network meta-analyses (PCV7 serotypes) or direct meta-analyses (PCV10 serotypes). Blue boxes and blue lines show the point estimates and confidence intervals of relative risk of seroinfection comparing PCV13 vs PCV10. The direct evidence column shows the percentage of evidence from studies directly comparing PCV13 vs PCV10. Results for PCV10 serotypes are from a meta-analysis of only head-to-head studies of PCV13 vs PCV10, therefore estimates of PCV7 vs PCV10 and PCV13 vs PCV7 were not available.

Supplementary Figure 59. Sensitivity analysis of relative risk of seroinfection for studies using a 3+1 schedule.


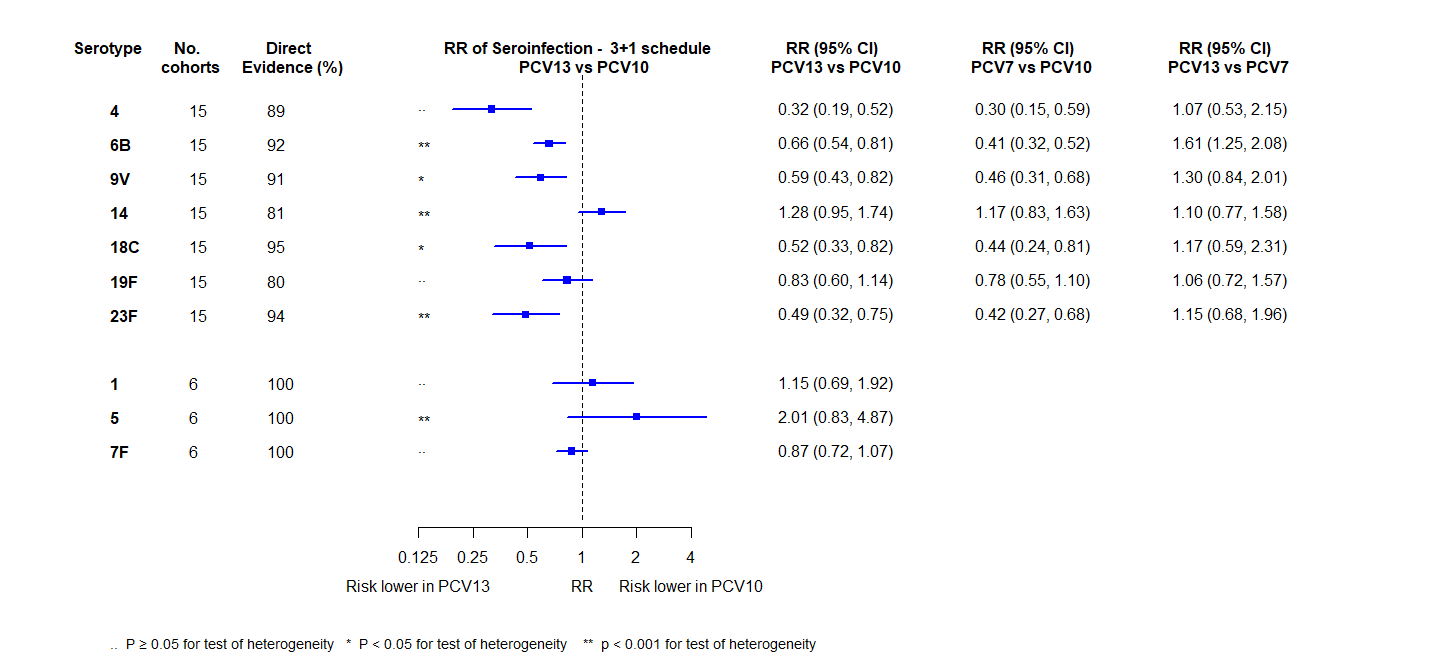


RR: relative risk; PCV: Pneumococcal conjugate vaccine. Each line in the figure shows the output from a network meta-analyses (PCV7 serotypes) or direct meta-analyses (PCV10 serotypes). Blue boxes and blue lines show the point estimates and confidence intervals of relative risk of seroinfection comparing PCV13 vs PCV10. The direct evidence column shows the percentage of evidence from studies directly comparing PCV13 vs PCV10. Results for PCV10 serotypes are from a meta-analysis of only head-to-head studies of PCV13 vs PCV10, therefore estimates of PCV7 vs PCV10 and PCV13 vs PCV7 were not available.

Supplementary Figure 60. Study level association between geometric mean ratio and relative risk for each serotype in PCV7


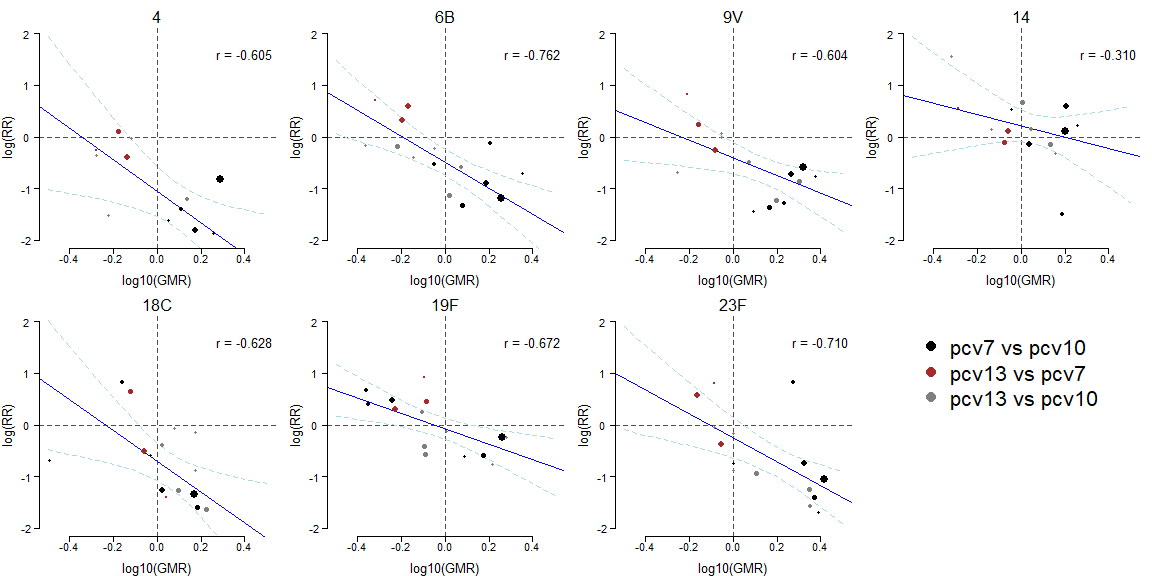


RR: relative risk; GMR: Geometric mean ratio; PCV: Pneumococcal conjugate vaccine. Each point shows results of a serotype specific head-to-head comparison between two vaccines from one study. Solid line shows the relationship between relative risk predicted from the crude model and geometric mean ratio. Dashed line shows the confidence intervals of predicted relative risk. Reference lines show geometric mean ratio equivalent to one (vertical) and relative risk equivalent to one (horizontal) which represent values associated with no difference between vaccines. Points sizes represent sample size of the trial. Each panel shows one PCV7 serotype (4, 6B, 9V, 14, 18C, 19F and 23F).
